# Enhanced protein isoform characterization through long-read proteogenomics

**DOI:** 10.1186/s13059-022-02624-y

**Published:** 2022-03-03

**Authors:** Rachel M. Miller, Ben T. Jordan, Madison M. Mehlferber, Erin D. Jeffery, Christina Chatzipantsiou, Simi Kaur, Robert J. Millikin, Yunxiang Dai, Simone Tiberi, Peter J. Castaldi, Michael R. Shortreed, Chance John Luckey, Ana Conesa, Lloyd M. Smith, Anne Deslattes Mays, Gloria M. Sheynkman

**Affiliations:** 1 Department of Chemistry, University of Wisconsin-Madison, Madison, WI USA; 2 Department of Molecular Physiology and Biological Physics, University of Virginia, Charlottesville, VA USA; 3 Department of Biochemistry and Molecular Genetics, University of Virginia, Charlottesville, VA USA; 4 Lifebit Biotech LTD., London, UK; 5 Department of Molecular Life Sciences, University of Zurich, Zurich, Switzerland; 6 Swiss Institute of Bioinformatics, University of Zurich, Zurich, Switzerland; 7 Channing Division of Network Medicine, Brigham and Women’s Hospital, Boston, MA USA; 8 Division of General Medicine and Primary Care, Brigham and Women’s Hospital, Boston, MA USA; 9 Department of Pathology, University of Virginia, Charlottesville, VA USA; 10 Institute for Integrative Systems Biology, Spanish National Research Council (CSIC), Paterna, Spain; 11 Microbiology and Cell Science Department, Institute for Food and Agricultural Sciences, University of Florida, Gainesville, FL USA; 12 Office of Data Science and Sharing, Eunice Kennedy Shriver National Institute of Child Health and Human Development, National Institutes of Health, Rockville, MD USA; 13 Center for Public Health Genomics, University of Virginia, Charlottesville, VA USA; 14 UVA Cancer Center, University of Virginia, Charlottesville, VA USA

**Keywords:** Long-read RNA-seq, PacBio, Mass spectrometry-based proteomics, Protein inference, Proteogenomics, Nextflow, Lifebit CloudOS, Alternative splicing, SQANTI, Iso-Seq

## Abstract

**Background:**

The detection of physiologically relevant protein isoforms encoded by the human genome is critical to biomedicine. Mass spectrometry (MS)-based proteomics is the preeminent method for protein detection, but isoform-resolved proteomic analysis relies on accurate reference databases that match the sample; neither a subset nor a superset database is ideal. Long-read RNA sequencing (e.g., PacBio or Oxford Nanopore) provides full-length transcripts which can be used to predict full-length protein isoforms.

**Results:**

We describe here a long-read proteogenomics approach for integrating sample-matched long-read RNA-seq and MS-based proteomics data to enhance isoform characterization. We introduce a classification scheme for protein isoforms, discover novel protein isoforms, and present the first protein inference algorithm for the direct incorporation of long-read transcriptome data to enable detection of protein isoforms previously intractable to MS-based detection. We have released an open-source Nextflow pipeline that integrates long-read sequencing in a proteomic workflow for isoform-resolved analysis.

**Conclusions:**

Our work suggests that the incorporation of long-read sequencing and proteomic data can facilitate improved characterization of human protein isoform diversity. Our first-generation pipeline provides a strong foundation for future development of long-read proteogenomics and its adoption for both basic and translational research.

**Supplementary Information:**

The online version contains supplementary material available at 10.1186/s13059-022-02624-y.

## Background

A comprehensive understanding of the proteome in healthy and diseased states is vital for nearly every area of biomedical research [1]. Multiple protein isoforms, containing distinct amino acid (AA) sequences, can arise from the same gene through mechanisms such as alternative promoter usage or splicing [2] and can exhibit different stabilities, molecular binding capabilities, and functional effects [3, 4]. Many protein isoforms have been implicated in diseases from neurodegeneration to cancer [5]. It has been estimated, through transcriptome measurements, that over 300,000 human protein isoforms may exist [6]. However, few experimental approaches readily detect proteins at isoform resolution, leaving open the question of the extent to which transcript isoform complexity propagates to the proteome [7, 8].

**Fig. 1:**
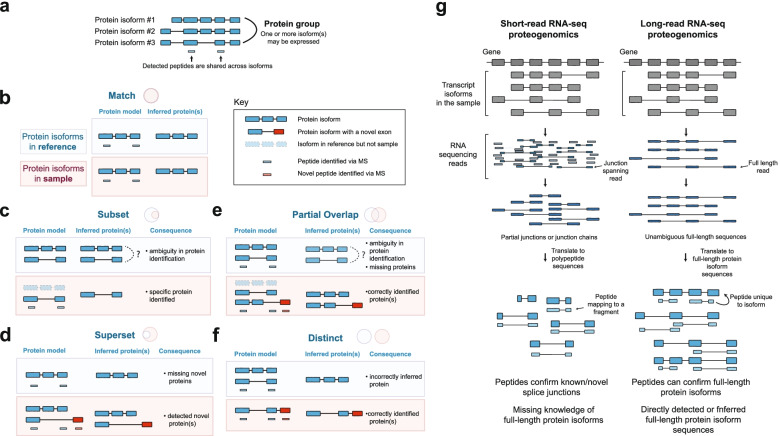
Challenges of protein isoform identification using MS-based proteomics. **a** Many peptides detected in MS-based proteomics map to multiple protein isoforms. Indistinguishable protein isoforms are represented as protein groups. This ambiguity limits the utility of MS-based proteomics for isoform detection. **b** The assumption of MS-based proteomics search algorithms is that the reference and sample isoforms match. Isoforms in the light blue boxes represent those annotated in a reference database. Isoforms in the light pink boxes represent isoforms that are actually expressed in a sample (which is unknowable using current technologies). When the reference and sample isoform are concordant (“Match”), protein identification can be accurate. **c–f** Reference-sample discordances can result in inferred proteins that are ambiguous or incorrect. Schematic of a case in which a sample contains a subset of isoforms in the reference database (“Subset,” **c**), additional isoforms (i.e., novel) not found in the reference database (“Superset,” **d**), a subset of isoforms but also additional novel isoforms (“Partial Overlap,” **e**), or only additional novel isoforms (“Distinct,” **f**). **g** Comparison of short versus long reads for proteogenomics analysis. Short-read RNA-Seq provides fragmented evidence of transcript isoforms, whereas long-read RNA-Seq provides full-length transcript sequences that can be used to predict full-length protein isoforms

Mass spectrometry (MS)-based proteomics has become the preeminent method for the comprehensive and sensitive characterization of the proteome [1]. Typically, the proteome is proteolytically digested into peptides that are analyzed via liquid chromatography (LC) and MS. The mass spectra are compared to theoretical peptides, generated from a protein database, to obtain peptide identifications. These peptide identifications are mapped back to their potential proteins of origin to obtain protein identifications (i.e., protein inference) [9]. Protein inference is complicated by shared peptides, which are peptides that map to two or more protein isoforms in the database. The presence of shared peptides can result in ambiguous protein identifications wherein multiple proteins are indistinguishable based on the peptide evidence. In these cases, a “protein group” (Fig. 1a) is formed, signifying either all or some subset of proteins in the group may be present in the sample.

The peptide identification and protein inference processes are heavily reliant on the composition of the protein database used for analysis. Reference protein databases broadly represent an organism’s proteome, but may fail to capture the proteomic variation across tissues, developmental and disease states, and individuals [10]. Discordances between a database and a sample can have a direct impact on proteomic search results. Ideally, the protein isoform sequences annotated in the reference for a gene would exactly match those expressed in a sample (“Match,” Fig. 1b). In practice, however, perfect matches are rare. The protein isoforms from a sample could differ from those in the reference by either lacking isoforms (“Subset,” Fig. 1c) and/or possessing a surplus of isoforms (“Superset,” “Distinct,” “Partial Overlap,” Fig. 1d–f). Overall, reference-sample discordances lead to (1) ambiguity in identifying protein isoforms; (2) incorrectly identified protein isoforms; or (3) failure to identify known or novel relevant protein isoforms (such as those associated to disease and treatment).

Transcript sequencing can be used to generate a sample-specific candidate protein database, which is more reflective of the isoform diversity in the sample than the reference database, but still has limitations due to the sensitivity and specificity of sequencing technologies. Presently, such efforts to generate sample-specific databases have been dominated by using short-read RNA-seq [11–20] which suffers from the inability to sequence full-length transcripts and can only deliver partial protein models [21, 22] (Fig. 1g). Long-read sequencing technologies, such as those from Pacific Biosciences (PacBio) and Oxford Nanopore Technologies (ONT), can delineate full-length transcriptomes with high fidelity [23]. These technologies can readily reveal thousands of novel isoforms based on full-length transcript reads [24]. Such developments present an opportunity to leverage transcript expression—a prerequisite and correlate of protein expression [25]—to enhance isoform-resolved proteomics.

Here, we present a workflow for long-read proteogenomics that achieves enhanced characterization of protein isoform diversity through paired long-read RNA-seq and MS-based proteomics of the same sample. This approach is enabled by a computational pipeline that generates full-length protein databases constructed de novo from long-read RNA-seq data. Using this database, we demonstrate MS-based discovery of novel protein isoforms arising from mechanisms such as retained introns and skipped exons. With full-length protein predictions, we introduce a new classification system, SQANTI Protein, to characterize novel protein isoforms. Finally, we introduce a new heuristic-based protein inference algorithm, called “Rescue & Resolve,” that incorporates long-read transcript abundance into the protein inference process, which enables detection of protein isoforms typically discarded during protein inference due to insufficient peptide support. The entire pipeline and workflow is freely available as an open-source and extensible computational resource, using the community-based workflow language, Nextflow. This first-generation long-read proteogenomics pipeline provides a strong foundation for the integration of long-read sequencing into proteomic workflows, advancing the characterization of human protein isoform diversity.

## Results

**Fig. 2:**
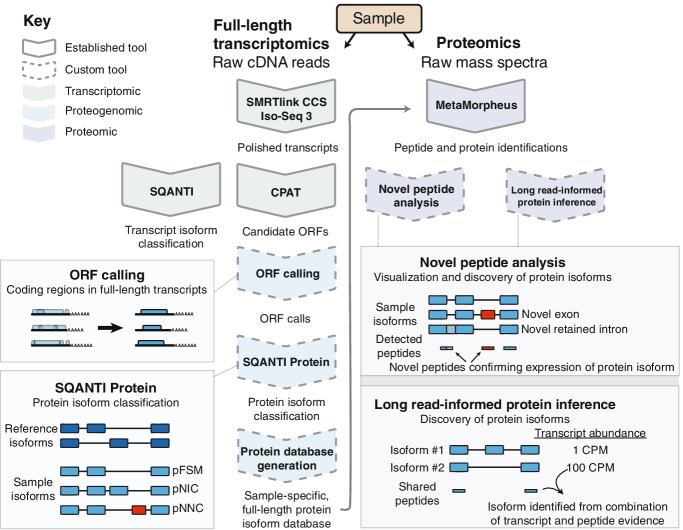
Long-read proteogenomic approach for enhanced sample-specific protein identification. Schematic of the long-read proteogenomics pipeline for improved protein isoform characterization. The pipeline includes approaches for ORF calling from long transcript reads, an automated protein isoform classification (SQANTI Protein), novel protein isoform detection, and a long-read-informed protein inference algorithm. CPM—full-length read counts per million

We developed a long-read proteogenomics pipeline for protein isoform detection through integrated analysis of sample-matched long-read RNA-seq and MS-based proteomics data. A Nextflow pipeline processes PacBio data, converts full-length transcripts into a protein database, and performs proteomics database searching (Fig. 2, Additional file 1: Fig. S1). We demonstrate the utility of our pipeline using transcriptomic and proteomic data from the same cell line, Jurkat T-lymphocyte. Below we describe the following: (1) analysis of PacBio sequencing to reveal high-quality full-length transcript sequences; (2) open reading frame (ORF) prediction; (3) a novel protein isoform classification system called SQANTI Protein; (4) generation of a sample-specific, full-length protein database using both PacBio and GENCODE reference isoform models; and (5) creation of a novel protein inference algorithm that increases the number of protein isoform identifications through the direct incorporation of PacBio transcript abundance values.

### Long-read RNA-seq reveals widespread isoform diversity that differs from the GENCODE reference set

We characterized the landscape of full-length transcripts in a human cell line through long-read RNA sequencing on the PacBio platform (see Additional file 2: Note S1). Transcript isoforms were compared to GENCODE [26] reference transcripts (v35), and their novelty status classified using SQANTI3 (Structural and Quality Annotation of Novel Transcript Isoforms) [27]. Among the transcript isoforms identified, 43,865 contained an exact match to GENCODE (“full splice matches,” FSMs) and 75,491 were novel. Of the novel cases, 43,075 transcripts contained novel combinations of known splice sites and/or junctions (“novel in catalog,” NICs), and 32,416 transcripts contained an entirely new splice site or exon (“novel not in catalog,” NNCs). On average, novel transcripts exhibit lower abundances than their known counterparts, despite exhibiting a broad range of abundances overall (Additional file 1: Fig. S2a). In 13.93% (1274) of genes, the most abundant transcript isoform is novel. To determine the sampling sensitivity of the transcriptome, we generated saturation-discovery curves and confirmed that the number of unique genes and isoforms detected reaches a plateau (Additional file 1: Fig. S2b). Overall, these results illustrate the widespread nature of alternative splicing and the need for empirically driven methods to characterize isoform diversity in human samples.

Note that for this study, transcript nucleotide sequences were derived from the reference genome (genome-corrected mode in SQANTI3); therefore, genetic variations are not captured in the current version of our pipeline (see “Discussion”).

### A sample-specific, full-length protein isoform database derived from long-read RNA-seq data

#### ORF prediction from long-read RNA-seq data

**Fig. 3:**
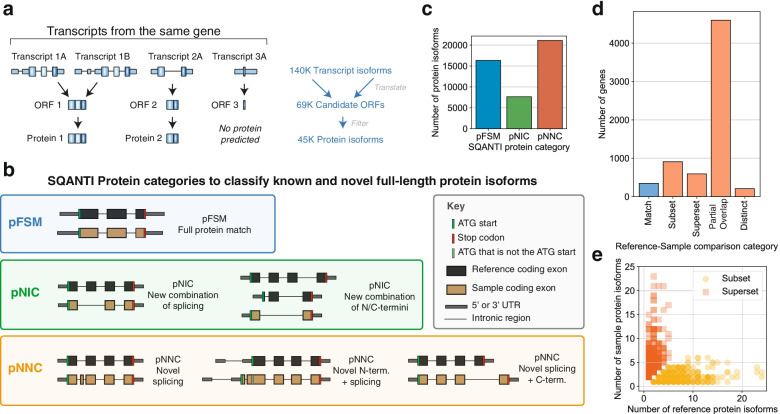
Generation and characterization of a long-read RNA-seq derived protein database. **a** Schematic demonstrating grouping of transcript isoforms to protein isoform database entries. Some distinct transcript isoforms may have identical coding regions, producing the same theoretical protein isoform product. **b** Schematic of the SQANTI Protein classification to compare long-read RNA-seq-derived protein isoforms to those annotated in the reference proteome. **c** Bar chart showing the frequency of protein isoform classifications for the protein database (total ~ 45,000 entries). **d** Number of genes in each category described in Fig. 1b–f, classified by the relationship between reference isoforms and predicted sample protein isoforms (genes in high confidence space). **e** Comparison of the number of sample versus reference isoforms for Subset and Superset isoform comparison scenarios. pFSM, protein full splice match; pNIC, protein novel in catalog; pNNC, protein novel not in catalog

We created a workflow to discern the most biologically plausible open reading frame (ORF) for each full-length transcript isoform. We considered multiple candidate ORFs for each transcript as defined by the Coding-Potential Assessment Tool (CPAT) [28]. For most of the transcripts (91%), one ORF stands out as the most plausible protein-coding product based on its coding score; however, a sizable number of transcripts (12,787 or 9% of all transcripts) have two or more relatively high scoring ORFs (CPAT coding score above 0.9), in which the best ORF is unclear (Additional file 1: Fig. S2c). Therefore, for all ORFs, we incorporated additional metrics in the ORF ranking process, such as the GENCODE annotation status of the ATG start codon and the start codon’s position relative to the 5′ end of the transcript (see “ORF calling” in “Methods” and see Additional file 2: Note S2). After determining the ORF prediction for each transcript, we clustered transcripts containing identical ORF predictions (Fig. 3a). Transcripts that differed only in their noncoding regions were assigned to the same protein entry in the database.

#### SQANTI Protein: new classification scheme for full-length protein isoforms

We derived protein isoform models from long-read RNA sequencing data for each gene and found that many genes may concurrently express multiple protein isoforms (Additional file 1: Fig. S2d). To systematically characterize these full-length protein isoforms, we created a new protein isoform classification scheme, SQANTI Protein, to describe the relationship between the predicted protein isoforms and those annotated in GENCODE. SQANTI Protein extends SQANTI3 transcript-centric classifications to the protein isoform level, considering how three key protein sequence elements—the N-terminus, the identified splice junctions, and the C-terminus—compare to reference protein isoforms (Fig. 3b). SQANTI Protein considers the full-length predicted protein sequence, detectable only by long-read RNA-seq, which differentiates it from previously proposed protein isoform classification schemas that have focused on “local” events, such as splice junctions or novel exons detected by microarrays or short-read RNA-seq [29, 30].

We loosely follow the nomenclature first developed for transcript isoform classification in SQANTI. Major isoform categories for SQANTI Protein include pFSM, pNIC, pNNC, and pISM (Fig. 3b). A “protein full splice match” (pFSM) represents a protein isoform where all elements exactly match at least one protein isoform in the reference. For a “novel in catalog” (pNIC) protein isoform, all protein sequence elements—such as the N-terminus, splice junctions, or C-terminus—are known (i.e., annotated in the reference), but the combination of elements is novel. A “novel not in catalog” (pNNC) protein isoform contains at least one novel element, such as a novel N-terminus or splice junction. Protein isoforms classified as an “incomplete splice match” (pISM) are cases in which the predicted protein isoform is a suspected artifact. For example, the originating transcript isoform could be degraded at the 5′ end, resulting in a translation product missing the true ATG start codon. More detailed protein isoform sub-classifications are provided in the “sqanti_protein” and “protein_classification” modules of the Nextflow pipeline.

Among the ORFs predicted from the long-read data, 16,331 (24%) have an exact GENCODE match and are deemed pFSMs (Fig. 3c). We found 28,737 (41%) potentially novel protein isoforms, with 7642 (11%) pNICs and 21,095 (30%) pNNCs. A more detailed breakdown of categorizations can be found in Additional file 3: Table S1. The remaining sequences were classified as pISM or were putative translation products of transcripts unlikely to be protein coding, such as intergenic transcripts.

It is notable that transcript-level classification does not always translate directly to the protein-level classification (Additional file 4: Table S2). For example, 371 transcript-level ISMs (ISMs) are actually protein-level FSMs (pFSMs). This occurs when part of the 5′ untranslated region (UTR) of a reference transcript is missing, but the ATG start codon is preserved. As another example, for 4086 known protein isoforms (pFSMs, 25% of total pFSMs), the originating transcript was novel (NIC or NNC) with novel splicing events exclusively occurring in the UTRs.

Predicted protein isoforms that are novel make up a substantial part of the database. For the majority of genes (75%), at least one pNIC or pNNC protein isoform was uncovered (Additional file 1: Fig. S2e). Furthermore, for a third of all genes with observed transcripts, the most abundant protein isoform did not correspond to the “reference” isoform (i.e., GENCODE APPRIS principal reference isoform [31], Additional file 1: Fig. S2f), and 42.5% (1215) of those isoforms were entirely novel.

After annotation with SQANTI Protein, 45,068 protein isoforms (pFSM, pNIC, and pNNC protein isoforms) from 10,348 genes were considered for database generation.

#### Defining a high-confidence PacBio-derived protein database

We generated a high-quality database for proteomic analysis with the following filtering criteria. Within our PacBio dataset, we found that genes producing transcripts with extreme lengths (e.g., less than 1 kb, longer than 4 kb), low abundance (e.g., below ~ 3 CPM, or full-length read counts per million), or without 3′ polyadenylation were not fully covered due to technical limitations (see Additional file 2: Note S3). Therefore, we used these criteria to select genes in which we were confident in the sampling of protein-coding transcripts. By extension, we are confident that the protein isoform models for these genes are reasonably complete. A total of 6653 genes meet our filtering criteria and are within the “high-confidence” space (HC space). For all other genes, we populated the protein database with GENCODE entries, generating a hybrid database to maintain integrity of downstream proteomic analysis. This hybrid database of PacBio-derived and GENCODE entries, called PacBio-Hybrid, is composed of 35,119 PacBio-derived protein entries from 6653 genes, and 48,413 GENCODE protein entries for the remaining 13,276 protein-coding genes (Additional file 1: Fig. S3a).

#### PacBio-derived protein isoform models for most genes differ from the reference

As described in the “Introduction,” differences between what is expressed in the sample and the reference database (see Fig. 1b–f; Match, Subset, Superset, Partial Overlap, Distinct) can have striking consequences on the protein isoforms inferred by MS analysis. Within the HC space, we found less than 5% of genes have PacBio-derived isoform models that exactly match the reference database (Fig. 3d). The most frequent database-sample discordance observed at a rate of 69% is “Partial Overlap,” in which the PacBio-derived database contains one or more reference-matched isoforms, but also contains additional novel isoforms. A total of 19,838 novel isoforms belong to genes in the “Partial Overlap” category. The other database-sample discordance categories which contain novel PacBio isoforms, “Superset” and “Distinct,” account for 8.9% and 3.1% of the genes in the database, respectively. Overall, the number of predicted protein isoforms for a given gene can diverge greatly between the sample-specific and reference database (Fig. 3e).

#### MS-based proteomics analysis with a PacBio-derived protein database

The PacBio-derived proteome differs substantially from the reference proteome. Since the database used for proteomic analysis serves not only as a model for identification but also for protein inference, its isoform composition directly impacts protein identifications. To assess such impacts, MS data from the Jurkat cell line was obtained and used for proteomic analysis with either the PacBio-Hybrid or GENCODE database. The MS spectra for analysis was generated via liquid chromatography-MS (LC-MS)/MS data-dependent analysis (DDA) of 28 fractions from high-pH reverse-phase liquid chromatography (RPLC) of a Jurkat tryptic digest. Acquired spectra were searched using the software tool MetaMorpheus [16] to obtain peptide- and protein-level identifications at a 1% false discovery rate (FDR) (Additional file 5: Table S3, Additional file 6: Table S4).

#### PacBio-derived protein database recovers peptides identified with the reference database

**Fig. 4:**
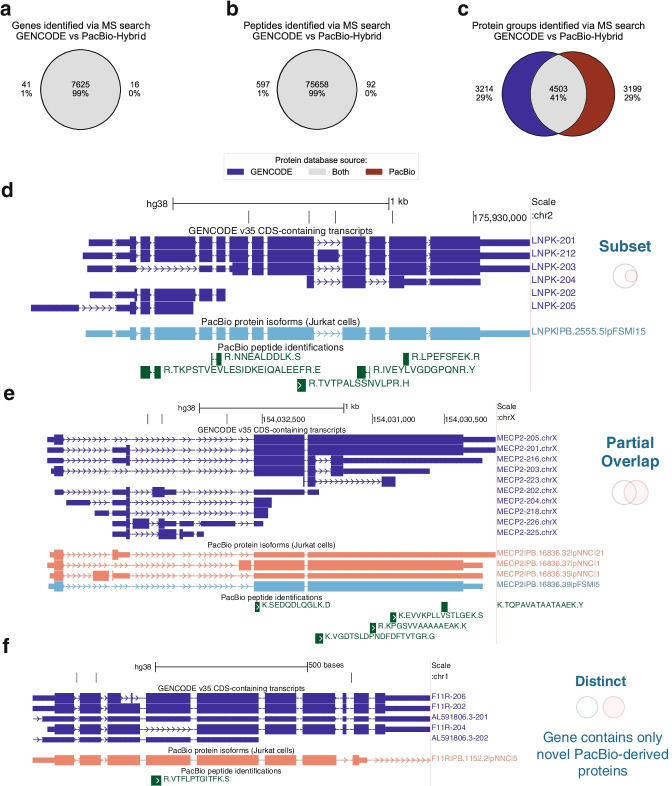
Customized long-read-derived protein database for protein isoform detection. **a–c** Overlap of peptide (**a**), gene (**b**), and protein isoform group (**c**) identifications from GENCODE versus PacBio database searches. **d** Example of a “Subset” case in which the sample is inferred to express fewer isoforms, based on the sample-specific PacBio-Hybrid database, than those inferred from the reference (GENCODE) database search. Based on the peptide evidence, the protein isoform expressed is ambiguous when relying on reference models, but precise (PB.2555.5 identified) when using the long-read database. **e** Example of a “Partial Overlap” case in which the sample expresses fewer isoforms than the reference but, at the same time, expresses additional novel isoforms not accounted for in the reference model. **f** Example of a “Distinct” case in which the sample expresses isoforms that are entirely distinct from those isoform models in the reference. Though the peptide maps to isoforms in the reference and sample, it is most likely arising from the novel protein isoform annotated from the long-read data. In **d–f**, the PacBio-derived isoform label follows this format: <gene>|<PB accession>|<SQANTI Protein class>|<CPM>. The peptide sequences display the flanking AA which is not part of the identified sequence. CDS, protein coding sequences

Notably, the proteomic results using the PacBio-Hybrid database recovered 99% of peptide and 99% of gene identifications found in the GENCODE reference database search results (1% FDR cut-off, Fig. 4a,b). Similar trends of results were observed when considering data from only the HC space, as well as when comparing PacBio-Hybrid results to search results obtained when using the UniProt reference database (Additional file 1: Fig. S3b-g). Additionally, the overlap between identified peptides and genes for the PacBio-Hybrid and reference database search results is comparable with the overlap found between the search results of the two reference databases (GENCODE vs. UniProt, Additional file 1: Fig. S3h-i) demonstrating that the PacBio-derived database is appropriately covering the protein space in the sample.

#### PacBio-derived isoform models lead to dramatically different protein isoform identifications and can resolve ambiguities

MS-based identification of protein isoforms is challenging due to the uncertainty in assigning shared (multi-mapping) peptides to their isoform(s) of origin. The protein database utilized for analysis should represent the protein isoforms in the sample, but differences between isoforms in the database versus the sample can impact the accuracy and precision of the inferred protein groups (see Fig. 1) [9].

We found that although the peptide and gene-level identifications between the PacBio-Hybrid and GENCODE MS search results were nearly 100% concordant (Fig. 4a,b), indicating that the peptide set for protein inference is nearly identical, there were major differences in the protein isoform identifications obtained (Fig. 4c). Only 41% (4503) of the protein isoform groups from both PacBio-Hybrid and GENCODE results were identical. Similar results were observed for comparisons of protein groups in the HC space, against the protein groups from the UniProt reference database search, and between the protein groups obtained from the two reference database searches (Additional file 1: Fig. S3j-m). This low overlap of protein inference results, across all comparisons, indicate that differences in protein identifications are primarily caused by differences in protein isoform composition of the databases.

The PacBio-derived database provides transcript-backed evidence of protein isoform expression that, when combined with peptide evidence, can lead to enhanced protein isoform identification. We found 3199 PacBio-Hybrid protein groups that are different from those protein groups inferred through the GENCODE reference search. Of these protein group differences, 673 cases (21%) result in increased specificity of protein isoform identification when using the sample-derived PacBio-Hybrid database. An illustration of this can be found in Fig. 4d. Based purely on MS peptide evidence, there is ambiguity in terms of whether the isoform LNPK-201 or LNPK-212 is expressed, but the PacBio transcript evidence indicates LNPK-201 is the main isoform likely to be expressed in the cell line. Another common scenario, accounting for 873 cases (27%), is that of partially overlapping protein isoform groups between the PacBio-Hybrid and reference results, as illustrated by isoforms of *MECP2* (Fig. 4e). Using the GENCODE database as reference, MECP2-205 and MECP2-201 form a single protein isoform group and are indistinguishable based on the peptide evidence. However, when using the PacBio-Hybrid database, there was no transcriptional support for MECP2-201. Instead, MECP2-205 forms a protein isoform group with the novel PacBio-derived isoform PB.16836.37. A third scenario, accounting for 382 cases (12%), occurs when all of the protein isoforms for a protein group in the PacBio-Hybrid analysis are absent from any protein groups within the GENCODE reference database analysis. This results in a protein group that is entirely distinct to the PacBio-Hybrid protein inference results. An example of this can be found in Fig. 4f, where the PacBio-derived database lists a single isoform which is not found in the reference database, representing a case of an entirely distinct isoform model.

For many of these cases, peptides were not detected in the isoform-specific regions, leading to a high dependence of protein isoform inference on the isoforms represented in the database. The isoform composition of a database has an outsize impact on the protein inference results obtained, and we believe that sample-specific databases improve the accuracy of protein isoform detection.

#### Characterization novel RUNX1 isoforms relevant to thymocyte biology

Within our data, we uncovered an excellent example of biologically relevant protein isoforms from *RUNX1* using full-length PacBio sequencing. *RUNX1* expresses a key transcription factor that regulates early thymocyte development [32, 33]. Rearrangements or mutations of *RUNX1* are associated with multiple hematopoietic neoplasms [34, 35]. Interestingly, recent evidence indicates germline mutations in *RUNX1* are associated with an increased risk of acute lymphoblastic leukemia (ALL) and that these mutations result in the generation of dominant negative isoforms of *RUNX1* [36]. The Jurkat cell line, analyzed here, is derived from a 14-year-old male patient with ALL [37]. Therefore, understanding the isoform landscape of *RUNX1* in our sample is highly relevant. Overall, we predicted 11 novel full-length protein isoforms of *RUNX1* (Additional file 1: Fig. S4). Eight of these predicted protein isoforms contain the complete DNA binding Runt homology domain (RHD) sequence expressed in-frame with novel downstream sequences (PB.15792.9, PB.15792.10, PB.15792.15, PB.15792.17, PB.15792.18, PB.15792.32, PB.15792.33, PB.15792.40). Additionally, five of these predicted isoforms (PB.15792.17, PB.15792.18, PB.15792.32, PB.15792.33, PB.15792.40) lack the transactivation domain (TAD) found in the longer *RUNX1* protein isoforms. The TAD recruits multiple cofactors (P300, CREBBP, TLE1) to *RUNX1*-binding sites, and thus each novel protein isoform has the potential to represent a functional dominant negative isoform capable of binding *RUNX1* sites but unable to recruit relevant cofactors that mediate gene activation or repression [35, 38]. Since full-length *RUNX1* is known to generally activate T cell differentiation genes and suppress multipotent hematopoietic genes [33], expression of these newly predicted dominant negative isoforms is consistent with supporting leukemogenic potential in Jurkat T-ALL. Peptide identifications provide support for the presence of three protein isoforms in two distinct protein groups. The two isoforms PB.15792.10 and PB.15792.15, containing both the RHD and TAD, are inferred as an indistinguishable protein group. Interestingly, PB.15792.40, one of the predicted dominant negative isoforms, is identified with a uniquely mapping peptide.

#### Long-read, sample-specific database leads to discovery of novel protein isoforms

**Fig. 5:**
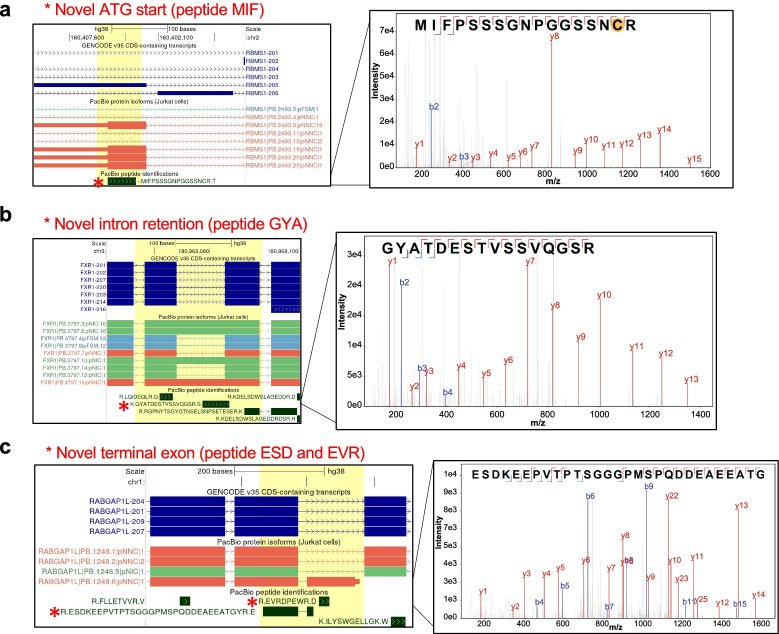
Discovery of novel peptides and full-length protein isoforms. **a** Novel peptide MIF confirms translation of an ATG start for RBMS1. **b** Novel peptide GYA confirms translation of a novel retained intron for FXR1. **c** Novel peptides ESD and EVR confirms the translation of a novel terminal exon for RABGAP1L. In this case, since the novel peptide maps exclusively to PB.1248.6, the corresponding full-length protein isoform is likely translated. Note that only ESD passed strict manual annotation, but EVR, which passed a 1% FDR in the global MS search, supports the expression of the same terminal exon

The MS search with the PacBio-Hybrid database revealed novel peptide sequences which were absent from both the GENCODE and UniProt reference databases. Stringent validation criteria were applied for novel peptide identifications and are described in more depth in Additional file 2: Note S4. We manually examined candidate mass spectra and confidently identified 14 novel peptides, each corresponding to a distinct event (Additional file 6: Table S4). Such events arose from a diversity of mechanisms, including upstream ATG start site usage, translation of a retained intronic region, and novel exons (Fig. 5a–c).

Notably, 6 of the 14 novel detected peptides each map to a single isoform and therefore there is direct evidence for expression of the corresponding full-length protein isoform. Such a direct link from peptide to full-length protein is only available with knowledge of full-length transcripts expressed in the sample [39]. An example of this is illustrated for the peptide, abbreviated as ESD, which confirms the novel terminal exon in *RABGAP1L*, but also unambiguously maps to the full-length PacBio-derived protein isoform PB.1248.6 (Fig. 5c). Only a small fraction of all potential novel protein isoforms are identified directly by a novel peptide. This is unsurprising based on previous reports regarding the detectability of isoform-specific tryptic peptides. The low peptide coverage of alternative isoforms could be technical in origin [40, 41], and the debate is ongoing regarding the extent to which novel transcript isoforms are translated into proteins [7, 8].

#### Long-read RNA-seq-informed protein isoform identification

In order to infer the presence of protein isoforms, most protein inference algorithms employ a probabilistic or parsimonious approach. Probabilistic protein inference algorithms seek to estimate the probability that a given protein isoform is in the sample on the basis of the peptides observed [42–45]. Parsimonious protein inference algorithms are more heuristically driven and follow Occam’s razor, which attempts to define the smallest number of protein isoforms that “covers” the set of identified peptides [9, 45–49].

**Fig. 6:**
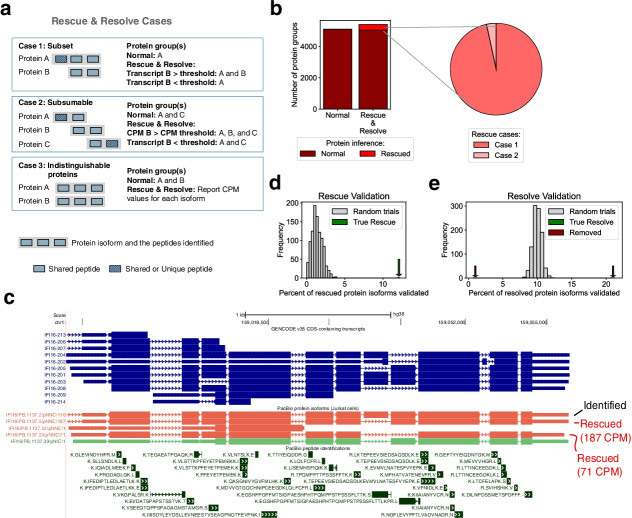
Long-read-informed protein isoform detection. **a** Scenarios in which long-read transcriptomic abundance values can be used to “rescue and resolve” protein group identifications for improved protein isoform detection. Note that peptide locations are not drawn to scale. See Ref. [10] for original cases. **b** The number of protein groups containing PacBio-derived protein isoforms identified in a traditional (normal) search versus one that incorporates long-read RNA-seq data to rescue isoforms (Rescue & Resolve algorithm). **c** Example of two rescued isoforms from the gene *IFI16*. PB.1137.5 and PB.1137.24 both have high transcriptional abundance and contain novel exon skipping events, but both lack unique sequence regions containing an identified peptide. CPM, full-length copies per million reads. **d, e** The percent of rescued (**d**) and resolved or removed (**e**) protein isoforms validated compared to the derived null distribution from randomly rescued isoforms

Parsimonious algorithms are commonly used in the MS proteomics field as part of search software platforms like Andromeda/MaxQuant and MetaMorpheus. However, this approach can lead to elimination of bona fide protein isoforms that lack sufficient peptide support relative to other isoforms (Fig. 6a) [50]. Alternative isoforms are particularly susceptible, because their isoform-specific regions comprise a small fraction of the proteome and suffer from a negative detection bias in traditional MS-based proteomics workflows using tryptic digestion [51].

In our tryptic dataset, the peptides observed at 1% FDR could be the digestion products of up to 26,931 different PacBio-derived protein isoforms in the high-confidence space. When traditional, parsimonious protein inference is applied to this peptide set, the number of PacBio-derived protein isoforms present in inferred protein groups drops to 11,231, eliminating 15,700 potential protein isoforms due to lack of sufficient peptide support. We hypothesize that a fraction of these eliminated protein isoforms may actually exist in the sample, and their elimination reduces the precision and accuracy of the protein inference results obtained.

#### Rescue & Resolve: direct incorporation of long-read data into protein inference

To overcome limitations of incomplete peptide coverage for protein isoform detection, we reasoned that the incorporation of long-read transcript isoform data directly in the protein inference process could help inform on the presence of a protein isoform. For this purpose, we developed a heuristic-based protein inference algorithm called “Rescue & Resolve” (R&R), which is implemented within a custom version of MetaMorpheus (see “Methods”). To our knowledge, this is the first protein inference algorithm that incorporates long-read transcriptional abundance as an orthogonal data source. As previously mentioned, the parsimonious protein inference process makes decisions throughout the algorithm to discard, or eliminate, protein isoforms from consideration for identification, because they lack the same level of peptide evidence that competing isoforms possess. During this process, protein isoforms that are actually present in the sample could be eliminated, generating false negatives. The “rescue” portion of our “R&R” algorithm defines two cases in which a protein isoform could be “rescued” from elimination (Fig. 6a). The first case occurs when a protein isoform’s mapped peptides are a subset of the peptides mapped to another protein isoform (Case 1, Fig. 6a). In this scenario, the parsimonious algorithm would determine that the protein isoform which accounts for the most peptides is the simplest answer, and therefore more likely to be correct by the principle of Occam’s razor. The protein isoform that accounts only for a subset of the peptides observed is eliminated from consideration for identification. The second case occurs when a protein isoform’s mapped peptides are subsumable to (i.e., can be explained by) two or more protein isoforms which have additional peptide evidence (Case 2, Fig. 6a). In this scenario, there is a protein isoform for which all of its peptide evidence can be explained by the existence of multiple protein isoforms that all have more peptide identifications supporting their existence. Again, as in Case 1, the parsimonious approach dictates that it is simpler, and therefore more likely, that the protein isoforms with additional peptide support are the sole contributors to the peptides being identified. The subsumable protein isoform is then eliminated from consideration for identification. In the “rescue” portion of our R&R algorithm, during the parsimonious process, protein isoforms that were eliminated due to scenarios such as Case 1 and Case 2, are identified, and set aside as potential false negatives that can be “rescued” from elimination. To determine whether or not a protein isoform should be “rescued” or eliminated, the long-read transcriptional abundance information obtained for each isoform is leveraged as an additional source of data. Since RNA abundance is at least moderately correlated with protein expression [25, 52] (*R*-squared = 0.65, Additional file 1: Fig. S5a), a high abundance transcript would have a higher probability, than a low abundance transcript, of generating the corresponding protein which was observed in our dataset (Additional file 1: Fig. S5b). In the R&R algorithm, protein isoforms are only rescued from elimination if their transcriptional abundance is greater than a user-specified abundance threshold. We selected a conservative transcript abundance threshold of 25 CPM (see Additional file 2: Note S5 for parameter optimization details). The impact of the “rescue” portion of the “Rescue & Resolve” algorithm on the protein inference results obtained were compared to those obtained with the traditional parsimonious protein inference algorithm within MetaMorpheus (details regarding MetaMorpheus’s inference algorithm can be found at https://github.com/smith-chem-wisc/MetaMorpheus/wiki/Protein-Parsimony-&-Grouping-(Protein-Inference)).

We rescued 355 protein groups, of which 343 (96.6%) are Case 1 and 12 (3.4%) are Case 2 (Fig. 6b). A common example, Case 1, is shown in Fig. 6c for isoforms of *IF116*, in which the dominant isoforms (PB.1137.5 and PB.1137.24) are not the isoform that contains the longest sequence (PB.1137.2). Notably, these isoforms are entirely novel, as compared to isoforms found in GENCODE. Collectively, the “rescued” protein isoforms represented a 6.5% increase in the number of PacBio-derived protein isoforms identified at 1% FDR, compared to what is obtained without the “R&R” algorithm, using MetaMorpheus’ traditional parsimonious approach. Validation of protein inference approaches is exceedingly difficult, in that we do not know the true composition of the sample, and standard protein mixtures lack the complexity necessary to model the human proteome. This is especially true in the case of modeling human isoform diversity where the “Rescue & Resolve” algorithm is most beneficial. To validate the accuracy of the “rescued” protein isoform identifications, we used an independent multi-protease MS dataset to generate a “ground truth” of protein isoform presence, enabling us to calculate the rate of validation of the “rescued” protein isoforms within the high coverage multi-protease dataset, as compared to the validation rate of a random control (see Additional file 2: Note S6). We observed that 12.2% of protein groups that were “rescued” were confirmed to be expressed in the multi-protease data, which is much greater than the average fraction of “rescued” protein isoforms validated from the distribution of the randomized control 1.4% (*N* = 1000 permutations, *p*-value < 0.0001, Fig. 6d). Details on the construction of the randomized control permutations can be found in Additional file 2: Note S6. Therefore, these results indicate that many true protein isoforms are rescued based on the incorporation of long-read sequencing knowledge.

The “resolve” portion of the R&R algorithm addresses a third scenario which can arise during protein inference (Case 3, Fig. 6a), where the parsimonious process generates ambiguity through a protein group which contains two or more indistinguishable protein isoforms (based on equivalent peptide evidence). Ambiguous protein groups can be composed of three different classes of isoforms categorized by their relative transcriptional abundance: (1) dominant (a “resolved” isoform), (2) minor, or (3) co-expressed. The “resolve” portion of the algorithm provides the opportunity to “resolve” these ambiguous protein groups to a single, dominant isoform, or provides support for the co-expression of multiple protein isoforms based on relative transcriptional abundance of each isoform within the group. For instances of Case 3, the relative transcriptional abundances underlying the predicted protein isoforms could indicate likelihood of expression.

We found 2600 cases (Case 3, Fig. 6a) of indistinguishable protein isoform groups in the high-confidence space, in which one or more protein isoforms are indistinguishable by peptide evidence alone. Our algorithm provides the relative transcript abundance measures for protein isoforms within a group, enabling the opportunity to resolve isoform identifications based on underlying transcript support, which is fully at the discretion of the user (Additional file 1: Fig. S5c). We found that in 1434 cases, one isoform comprises more than 90% of the transcript abundance, suggesting that a single dominant isoform could comprise the group. For these dominant isoform-containing protein groups, the ambiguity of which protein isoform is present within the sample was resolved, and a single protein isoform was considered to be identified, increasing the precision of the protein inference results obtained. Notably, not all protein groups can or should be resolved to a single isoform. There are cases where multiple protein isoforms are co-expressed and the peptide evidence is not comprehensive enough to be able to sufficiently distinguish them. It is important to maintain protein group ambiguity when necessary and valid. We discovered 295 protein isoform groups in which multiple protein isoforms may be co-expressed at appreciable levels (2+ isoforms with relative abundance > 30%), indicating that a single representative isoform cannot be assumed for these cases. We validated the accuracy of the “resolved” protein isoform identifications by applying the same multi-protease validation strategy used for “rescued” protein isoforms (see Additional file 2: Note S6). We observed that 21.2% of the “resolved” protein isoforms were confirmed to be expressed in the multi-protease data, which is much greater than the average fraction of “resolved” proteins validated from the distribution of the randomized control, 10.0% (*N* = 1000 permutations, *p*-value < 0.0001, Fig. 6e). Details on the construction of the randomized control permutations can be found in Additional file 2: Note S6. We also investigated the validation rate of the protein isoforms that were removed from the protein groups, to determine if their removal was justified. We observed that only 0.7% of the removed isoforms were confirmed to be expressed in the multi-protease data. This is much less than the average fraction of “resolved” proteins validated from the distribution of the randomized control and the validation rate of the experimentally “resolved” protein isoforms (Fig. 6e). Although the majority of the “resolved” protein isoforms (73%) are incapable of producing a detectable unique peptide (7 to 50 amino acids) in any of the six protease digests (Arg-C, Asp-N, Chym, Glu-C, Tryp, and Lys-C), 86 of the 387 (22%) “resolved” isoforms capable of producing a theoretical unique peptide were confirmed by the identification of a unique peptide identified in the multi-protease dataset. All “rescued” and “resolved” groups may be found in Additional file 7: Table S5.

These results indicate that the incorporation of long-read transcriptional abundance values into the protein inference process reveals protein isoforms that were difficult to identify solely with MS peptide data.

## Discussion

The comprehensive characterization of the cellular proteome is a major goal in proteomics to understand the molecular underpinnings of normal and disease states. One factor impeding progress towards this goal is the lack of experimental approaches that can easily identify proteins at isoform resolution. Current efforts employ short-read RNA-seq approaches which cannot characterize full-length isoforms [22]. Long-read sequencing provides the ability to obtain full-length transcript reads [23], allowing the delineation of transcript isoforms and, therefore, potential full-length protein isoforms for MS analysis [39, 53, 54].

To our knowledge, this is the first long-read based proteogenomics pipeline that integrates full-length transcripts with MS data for full-length protein isoform characterization. We show that the availability of long-read-derived, sample-specific protein isoform models is critical to enhance protein isoform detection. Our pipeline produces sample-specific, full-length protein isoform databases which enables novel peptide discovery, and outputs genome browser tracks for visualization of reference- and sample-derived isoforms as well as peptide identifications. The pipeline also includes the first protein inference algorithm to directly incorporate long-read sequencing data to detect protein isoforms heretofore intractable to MS analysis (“Rescue & Resolve”).

Integrating long-read sequencing and proteomic data presented new challenges, which we addressed through the development of new components in the pipeline. We defined for each full-length transcript the most likely canonical ORF based on a modified output of CPAT. Further, we created a new protein isoform classification system, SQANTI Protein, based on the transcript isoform classification tool SQANTI3. Finally, the “Rescue & Resolve” algorithm, through incorporation of long-read transcript isoform expression data into the protein inference process, enables the “rescue” of protein isoforms that have significant transcriptional support but are nonetheless difficult to identify in MS due to high sequence overlap. The algorithm also enables the user to “resolve” ambiguous protein isoforms that are indistinguishable based on peptide evidence alone, by leveraging the relative transcriptional abundance for such isoforms.

Our workflow identified 45,068 distinct candidate protein isoforms from a human cell line (Jurkat cells), 22,807 of which were novel. These long-read sequencing-derived protein isoforms were filtered, and a sample-specific PacBio-Hybrid database containing 35,119 PacBio-derived protein isoform entries was generated. Proteomic analysis of this database revealed 14 novel peptide identifications and 5100 protein isoform groups within the high-confidence space identifications at 1% FDR. Notably, one of the novel peptides confirmed the translation of a transcript with a retained intron, which highlights the utility of an empirical approach to uncover the translation of transcripts not commonly thought to be translated. The implementation of the heuristic-based Rescue & Resolve protein inference algorithm increased the number of PacBio-derived protein isoform groups identified by 355, and resolved 1434 ambiguous protein isoform groups to a single protein isoform identification. The resolve approach also highlighted the existence of 295 protein isoform groups in which multiple protein isoforms appeared to be co-expressed at appreciable levels (2+ isoforms with relative abundance > 30%), demonstrating it is not always appropriate to assume a single isoform is expressed [14]. Although the Rescue & Resolve algorithm was developed for use with long-read sequencing information, the algorithm could also be applied to proteogenomic databases and transcriptional abundance information derived from short-read sequencing approaches.

The results and concepts described here provide a foundation for future development of long-read proteogenomics. The pipeline’s flexible and modular nature lends itself to adaptation. For example, the proteomic analysis portion of the pipeline could be expanded to include a semisupervised learning post-search program such as percolator [55] or mokapot [56]. In the future, we plan to expand the custom ORF prediction algorithm to include the discovery of noncanonical ORFs, such as those with cognate start sites (e.g., CTG) or short upstream ORFs commonly found in the 5′ UTR [57–59]. Another improvement to the pipeline will be an evolution of the heuristically driven “Rescue & Resolve” approach. We plan to develop a probabilistic protein inference algorithm in which transcriptional abundance values are incorporated into a rigorous statistical framework for the inference of protein isoforms [43, 60]. The applications of our computational pipeline could also include the analysis of novel genes or genetic variation that is detectable in long-read data or separately available from previous genotyping, use of ONT (i.e., nanopore) cDNA or direct RNA sequencing data [54], the analysis of single-cell RNA-seq, use of targeted long-read datasets [61], or the use of top-down proteomics data for the analysis of proteoform diversity [62].

Though long-read proteogenomics and its application hold promise, limitations remain. First, for the “Rescue and Resolve” approach, we assume at least a moderate degree of RNA-protein correlation. Although isoforms from the same gene should not greatly differ in their transcript-protein correlation, several studies have reported isoform-specific mRNA translation [63, 64] suggesting that alternative splicing can generate transcripts with distinct cis-regulatory landscapes. Therefore, caution must be taken for any given protein isoform, including follow-up confirmation of expression in vivo. Second, as with any RNA-Seq-based dataset, even though a majority of the isoform diversity detected from long-read RNA-seq approaches are likely due to co- and post-transcriptional processing mechanisms, it is possible that genetic translocations, deletions, or other mutations may give rise to what is ostensibly transcript isoform variations that are actually genetic in origin. We used Jurkat cells as a model system, which is tetraploid, and may contain some isoform variations due to cancer-related or natural genetic variants [65]. Third, the pipeline results are dependent on the quality of long-read RNA sequencing. Limitations in quality of the extracted RNA or artifacts generated during the sample handling and library preparation process (e.g., PCR artifacts) can detrimentally impact accuracy of predicted protein models. The sampling of full-length transcripts is known to be incomplete—ultra-long transcripts or those transcripts lacking a polyA tail may be under sampled—and can impede the ability to derive the entire proteome from transcript data alone. However, as both ONT and PacBio sequencing improves in both coverage and sensitivity, an entire long-read-derived proteome should be able to be generated de novo from sample-specific transcriptomes. Furthermore, rigorous benchmarking studies, such as those being conducted by The Long-read RNA-seq Genome Annotation Assessment Project (LRGASP) Consortium, will reveal strength and limitations of these methods for the community [66].

Overall, the incorporation of long-read sequencing into proteogenomic workflows represents a tremendous opportunity for isoform-resolved investigations in basic and translational research. As long-read sequencing continues to evolve in throughput, accuracy, and accessibility, long-read proteogenomics will be adopted by researchers and clinicians and become a routine practice in the context of precision medicine.

## Conclusion

We show that sample-specific protein isoform models derived from long-read RNA-seq can lead to enhanced protein isoform detection. Our pipeline enables novel peptide discovery and outputs genome browser tracks for visualization of reference- and sample-derived isoforms as well as peptide identifications. We introduce the first protein inference algorithm that directly incorporates long-read sequencing data to detect protein isoforms heretofore intractable to MS analysis (“Rescue & Resolve”). This work represents a foundation for subsequent studies that integrate long-read RNA-seq with proteomics for protein isoform characterization.

## Methods

### PacBio long-read RNA-seq

PacBio (Iso-Seq) data was collected on the Jurkat T-lymphocyte cell line. Jurkat RNA was procured from Ambion (Thermo, PN AM7858). The RNA was analyzed on a Thermo Nanodrop UV-Vis and an Agilent Bioanalyzer to confirm the nominal concentration and ensure RNA integrity. We observed a RIN value of 9.9. From the RNA, cDNA was synthesized using the NEB Single Cell/Low Input cDNA Synthesis and Amplification Module (New England Biolabs).

Approximately 300 ng of Jurkat cDNA was converted into a SMRTbell library using the Iso-Seq Express Kit SMRT Bell Express Template prep kit 2.0 (Pacific Biosciences). This protocol employs bead-based size selection to remove low mass cDNA, specifically using an 86:100 bead-to-sample ratio (Pronex Beads, Promega). Library preparations were performed in technical duplicate. We sequenced each library on a SMRT cell on the Sequel II system using polymerase v2.1 with a loading concentration of 85pM. A 2-h extension and 30-h movie collection time was used for data collection. The “ccs” command from the PacBio SMRTLink suite (SMRTLink version 9) was used to convert Raw reads (~ 6 million, over 349 Gbps) into Circular Consensus Sequence (CCS) reads. CCS reads with a minimum of three full passes and a 99% minimum predicted accuracy (QV20) were kept for further analysis.

### Jurkat RNA-Seq data download and analysis

Jurkat RNA-Seq data was previously collected on an Illumina HiSeq2000, generating ~ 38.8 million paired-end 150 bp reads [67]. The data was downloaded from GEO (GSE45428).

To obtain estimated gene and isoform-level abundances, Kallisto (version 0.44.0) was used, with raw reads and the GENCODE reference transcriptome (version 35, GTF file of the comprehensive set, protein-coding genes only) as input.

### Mass spectrometry data collection

Bottom-up proteomic data was previously collected for the multi-protease and trypsin-only data sets [48, 68]. Briefly, cells were cultured and processed with aliquots of approximately 10^7^ cells each (6 aliquots for multi-protease digest and 1 aliquot for trypsin digest). Aliquots were lysed in SDT buffer (4% SDS, 500 mM Tris-HCl (pH 7.4) and 180 mM DTT) and approximately 150 μg of lysate was used for filter-aided sample preparation [69]. Each aliquot for the multi-protease data set was digested with a different protease (Arg-C, Asp-N, chymotrypsin, Glu-C, Lys-C, or trypsin), and the trypsin-only aliquot was digested using trypsin. Following digestion, peptides were fractionated off-line by high-pH reverse-phase liquid chromatography (trypsin-only: 28 fractions, multi-protease: 11 fractions–10 fractions for the second trypsin sample) and dried down. Fractions were then reconstituted in 5% acetonitrile and 1% formic acid prior to LC-MS/MS analysis on a nanoACQUITY LC system (Waters, Milford, MA) interfaced with a Thermo Scientific LTQ Orbitrap Velos mass spectrometer. All mass spectrometry raw files are freely available online (multi-protease: https://massive.ucsd.edu/MSV000083304/; 28 fraction trypsin: https://db.systemsbiology.net/sbeams/cgi/PeptideAtlas/PASS_View?datasetPassword=RE4343upo&identifier=PASS00215).

### PacBio Iso-Seq data analysis

Raw reads obtained from PacBio Sequel II sequencing were processed into “High Fidelity” (HiFi/CCS) reads using the “ccs” command in SMRTLink. Following CCS read generation, the “lima” command was run to generate full-length reads containing both the 5′ and 3′ primer. The 5′ primer consists of the NEB cDNA sequence (sequence: GCAATGAAGTCGCAGGGTTGGG). The 3′ primer consists of the Clontech SMARTer cDNA primer (sequence: GTACTCTGCGTTGATACCACTGCTT). Following “isoseq3 refine” processing, polyA tail sequences are removed. Then, “isoseq3 cluster” is run in order to cluster full-length reads that correspond to the same transcript isoform. This process allows for generation of full-length, non-concatamer (FLNC) reads, which are subjected to further downstream processing, as described below.

The high-quality, polished transcript sequences were mapped to hg38 using minimap (pbmm2, version 1.4.0) [70] with the following parameters “--preset ISOSEQ –sort”. Finally, “isoseq3 collapse” was run in order to combine redundant reads which were not properly clustered in the “isoseq3 cluster” step.

We recovered the relative abundance of each of the final isoforms in each sample by extracting the number of full-length reads supporting each polished isoform. Full-length counts per million (CPM) were derived by dividing the number of full-length non-chimeric reads aligning to a transcript isoform (i.e., the read became part of the transcript during the isoform clustering step) by the total number of reads and multiplying by a factor of 1,000,000. Only transcripts above one CPM were subjected to further analysis in this study.

### Transcript isoform classification and filtering

SQANTI is a computational tool for classification and quality assessment of full-length isoforms sequenced on long-read platforms [27]. We used SQANTI3 version 1.3 to annotate the polished transcript isoforms obtained from the Iso-Seq analysis. We used default parameters. Note that this includes the option to use genome-derived sequences for the isoform output; therefore, transcriptional variations (alternative N-termini, alternative splicing, etc.), but not genetic variations, will be captured in the current version of our pipeline.

The inputs for SQANTI3 analysis include the GENCODE version 35 annotations (i.e., GTF file) and the human reference genome (GRCh38, only canonical chromosomes chr1-22, X, Y). The SQANTI3 outputs—isoform and junction “classification” files—were subjected to additional analysis using custom python scripts, which are part of the Nextflow pipeline.

After running SQANTI3, we filtered out any transcript that was (1) classified as a RT-template switching artifact by SQANTI3, (2) had 95% or higher Adenosine (i.e., polyA) content in 20 nt of the genome immediately downstream of the aligned 3′ end of the transcript, indicating a possible dT intra-priming artifact, or (3) did not align to a GENCODE-annotated protein-coding gene (while SQANTI3 does not exclude transcripts based on coding potential, for the purpose of this study, we have excluded them). Finally, we employed a modified version of Cupcake “filter_away_subset.py” (https://github.com/Magdoll/cDNA_Cupcake) to remove 5′ transcript degradation products.

### Generation of a full-length protein isoform database from long-read RNA-seq

#### ORF prediction

After deriving a high-confidence set of full-length transcript isoforms, we developed a pipeline for selection of the most biologically plausible canonical ORF for each Iso-Seq transcript (“orf_calling” module in the Nextflow pipeline).

The Coding-Potential Assessment Tool (CPAT) was used to find all candidate open reading frames (ORFs), allowing up to 50 candidate ORFs of 50 nt or longer. The metrics in the CPAT result output (e.g., coding score, which incorporates a hexamer score, ORF length and other metrics) were used for subsequent derivation of a final score for each candidate ORF. Additional information on ATG start codon status was used to generate this final score. For each candidate ORF, the ATG start codon was determined and compared to the GENCODE-annotated ATG start codon. It is difficult to predict the exact ATG start ab initio due to lack of a strong consensus sequence for translational initiation sites genome-wide, but the identity of at least some of these sites has been manually curated where literature evidence exists (e.g., HAVANA group, GENCODE). Therefore, any ORF containing an ATG start previously annotated by GENCODE was selected in all cases. In the case that there are multiple ORFs corresponding to two or more GENCODE proteins, we selected the upstream-most ORF. Otherwise, the number of ATGs found upstream of the candidate ORF start site was determined for incorporation into the final scoring metric. Note that this final score employed heavy weighting for ORFs with ATG start sites closer to the 5′ end of the PacBio transcript.

#### Protein database compilation

To generate a PacBio-derived protein database for MS searching, we grouped transcripts that produce ORFs (i.e., proteins) of the same sequence (“refine_orf_database” module in the Nextflow pipeline). Within each transcript grouping, a representative or “base” PacBio accession was chosen based on alphanumeric sorting. The total transcript abundance for each grouping is the sum of all CPM values for member transcripts.

A FASTA file was generated containing in the accession line the base Iso-Seq accession and gene name. In addition to the FASTA file, a metadata table (“jurkat_orf_refined.tsv”) was generated containing information on the base Iso-Seq accession, all other accession(s) in the same protein sequence group, the gene to which the isoform mapped, and the aggregated CPM.

#### GENCODE reference protein database

The GENCODE protein database used in this study was created by downloading the protein-coding translation FASTA and grouping entries with the same protein sequence for each gene (see “make_gencode_database” module in the Nextflow pipeline). There are many cases in which one or more GENCODE transcripts from the same gene lead to the same protein sequence. We grouped such cases and defined a representative protein accession as the first alphanumeric GENCODE protein accession, by transcript name (e.g., GAPDH-201).

#### Cross-mapping of protein isoforms across databases

To compare protein isoform entries across the sample-specific (PacBio-derived) and reference (GENCODE, UniProt) databases, we performed a standard sequence-alignment-based mapping (see “accession_mapping” module in the Nextflow pipeline). Specifically, a pairwise alignment of all proteins between databases is conducted, tolerating up to two AA mismatches. Up to two AA differences are tolerated since the three databases originate from different sources of genomic or transcript nucleotide sequence. For example, GENCODE protein sequences are derived from the human reference genome, while many UniProt sequences were derived from cDNA sequences. The mapping was done in an iterative manner, in which perfect alignments (i.e., end-to-end match, no AA differences) were first sought and any remaining unmapped entries were compared to the other databases allow for first a single AA and then (if still unmapped) two AA mismatches. Any entries with differing protein lengths or with more than two AA mismatches were considered distinct entries.

#### Mass spectrometry searching against the PacBio-derived and GENCODE database

Standard proteomic analysis of the tryptic and multi-protease datasets was performed using the free and open-source search software program MetaMorpheus [71]. A custom branch and docker image of MetaMorpheus was created (GitHub: https://github.com/smith-chem-wisc/MetaMorpheus/tree/LongReadProteogenomics, Docker: https://hub.docker.com/r/smithchemwisc/metamorpheus/tags?page=1&ordering=last_updated tag: lrproteogenomics) based on MetaMorpheus version 0.0.316 which includes a novel protein inference algorithm termed “Rescue & Resolve.” Analysis was performed using either the sample-specific hybrid (PacBio+GENCODE, called “PacBio-Hybrid”) database (83,532 protein entries from 19,929 genes; in which the subset of PacBio-derived entries are 35,119 protein entries from 6653 genes), the GENCODE human database (version 35; 87,729 protein entries from 19,929 genes), or the UniProt reviewed human database with isoforms (downloaded November 1st, 2020; 42,358 protein entries from 20,292 genes). All searches were conducted with a contaminants database, included in MetaMorpheus, which contains 264 common contaminant proteins frequently found in MS samples.

All RAW spectra files were first converted to MzML format with MSConvert (centroid mode) prior to analysis with MetaMorpheus (see “mass_spec_raw_convert” module in the Nextflow pipeline). For the MetaMorpheus MS search, the settings used for all search tasks can be found in Additional file 8: Table S6. MetaMorpheus produces peptide spectral match (PSM), peptide and protein group result files, which we analyzed in downstream custom modules. Peptide identifications constitute not only the base amino acid sequence but also any post-translational modifications. Two separate peptide identifications may be present for the same base sequence, but exist as the modified and unmodified form. All peptide and protein results reported employ a 1% false discovery rate (FDR) threshold after target-decoy searching [72].

Computational pipeline with NextflowWe implemented the long-read proteogenomic pipeline in Nextflow, a domain-specific language allowing for the highly flexible development of bioinformatic pipelines capable of being deployed on local machines, servers, or cloud environments [73]. The ability to create distinct modules for different analyses through containerization (e.g., Docker) is a key benefit of this framework, enabling both the seamless integration of long-read RNA-seq and mass spectrometry analysis workflows and the flexibility to collaborate across research groups. These processes are automatically parallelized for optimal efficiency of compute resources.

We developed a Nextflow pipeline to process PacBio data, convert resulting transcripts into a protein database, and perform proteomics database searching. The workflow, including all source code, is publicly available in GitHub at https://github.com/sheynkman-lab/Long-Read-Proteogenomics [74]. All docker images may be found in the Docker Hub (https://hub.docker.com/) under the repository gsheynkmanlab.

The analyses were performed on the Lifebit CloudOS platform (link: https://lifebit.ai/), and the analysis page is available with the shareable link https://cloudos.lifebit.ai/public/jobs/60bcb29b303ee601a69d8c74. The pipeline structure, including details for each module, is included in Additional file 1: Fig. S2. Modules can represent a previously established program, a modified program, or a customized script for either processing or analysis. The full details may be found in the Long-Read-Proteogenomics GitHub Wiki page https://github.com/sheynkman-lab/Long-Read-Proteogenomics/wiki.

### Data analysis and plot generation

All downstream data analyses were performed through custom Python and/or C# scripts. Data analysis scripts used for figure generation may be found in the following GitHub repository: https://github.com/sheynkman-lab/Long-Read-Proteogenomics-Analysis [75].

## Supplementary Information

**Additional file 1: Figure S1. **Detailed schematic of the Nextflow computational pipeline forlong-read proteogenomics. **Figure S2. **Generation and characterization of candidate protein isoform sequencesfrom long-read RNA-seq data. **Figure S3. **Comparison of MS-based proteomic coverage when using differentprotein databases for MS searching. **Figure S4. **Novel isoforms detected for genes key to thymocyte tumor biology. **Figure S5. **Relationship between RNA and protein estimated abundances. 

**Additional file 2: Notes S1-S5.** Supplementary notes for the manuscript [31, 48, 71, 80–93].

**Additional file 3: Table S1.** Detailed breakdown of protein classifications based on SQANTI Protein. Provides information on the key protein elements which differ from the GENCODE reference.

**Additional file 4: Table S2.** Number of isoforms for each transcript and protein isoform classifications between SQANTI and SQANTI Protein (FSM = full-splice_match; ISM = incomplete-splice_match; NIC = novel_in_catalog; NNC = novel_not_in_catalog). Note that protein-centric classifications are prepended with a “p” (e.g., pFSM).

**Additional file 5: Table S3.** Summary of MetaMorpheus search results on the gene, peptide and protein levels at 1% FDR for the PacBio-Hybrid, GENCODE and UniProt databases.

**Additional file 6: Table S4.** Detailed information for all high confidence novel peptide identifications such as: their annotated spectra, novel peptide classification and isoform track image.

**Additional file 7: Table S5.** Protein groups (list of the PB accessions) which contain a rescued or resolved isoform.

**Additional file 8: Table S6.** Search parameters for all MetaMorpheus proteomics searches.

**Additional file 9.** Review history.

## Data Availability

All materials, including data used, workflows and analysis notebooks are available in full accordance with the NIH Grants Policy Statement and the Principles and Guidelines for Recipients of NIH Research Grants and Contracts (https://grants.nih.gov/policy/sharing.htm). Third-party datasets used in this manuscript include short-read Jurkat RNA-seq data (Gene Expression Omnibus GSE45428) and bottom-up mass spectrometry data for Jurkat cells (PeptideAtlas: PASS00215, ProteomeExchange: PXD012272). Raw long-read RNA-seq data collected on the PacBio platform are available from the Sequence Read Archive (PRJNA783347, corresponding to accessions SRX13222302 and SRX13222303) [76]. Data generated by both mass spectrometry and long-read RNA sequencing used in the execution of results for this work are available on Zenodo (10.5281/zenodo.5703754) [77].  The long-read proteogenomics workflow results generated using the mass spectrometry and long-read RNA-sequencing data are available on Zenodo (10.5281/zenodo.5987905) [78]. The open-source software produced in the making of this work is freely available under the MIT license found in the GitHub repository (https://github.com/sheynkman-lab/Long-Read-Proteogenomics) [74]. The workflow language used in the generation of the results was Nextflow (http://nextflow.io) and the long-read proteogenomics workflow  m ay be found in the repository (https://github.com/sheynkman-lab/Long-Read-Proteogenomics/main.nf). A README (https://github.com/sheynkman-lab/Long-Read-Proteogenomics/blob/main/README.md) is located in the repository, guiding the user to the Wiki (https://github.com/sheynkman-lab/Long-Read-Proteogenomics/wiki) describing each of the pipeline processes (https://github.com/sheynkman-lab/Long-Read-Proteogenomics/wiki/Pipeline-Processes) and provides for pipeline vignette (https://github.com/sheynkman-lab/Long-Read-Proteogenomics/wiki/Pipeline-Vignette). Test data used in the pipeline vignette and with the GitHub actions run to ensure workflow integrity through continuous testing are available on Zenodo (10.5281/zenodo.5234651) [79]. Code used to generate the main figures and tables in this manuscript can be found in the GitHub repository (https://github.com/sheynkman-lab/Long-Read-Proteogenomics-Analysis) [80]. All containers used in the workflow are located in Dockerhub (https://hub.docker.com/r/sheynkmanlab/long-read-proteogenomics).
