# Supplementary material for: Enhanced protein isoform characterization through long-read proteogenomics
Source: Genome Biol. 2022 Mar 3;23:69. doi: 10.1186/s13059-022-02624-y (PMC8892804; doi:10.1186/s13059-022-02624-y)
Supplement: Supplementary file 1 — Additional file 1: Figure S1. Detailed schematic of the Nextflow computational pipeline forlong-read proteogenomics. Figure S2. Generation and characterization of candidate protein isoform sequencesfrom long-read RNA-seq data. Figure S3. Comparison of MS-based proteomic coverage when using differentprotein databases for MS searching. Figure S4. Novel isoforms detected for genes key to thymocyte tumor biology. Figure S5. Relationship between RNA and protein estimated abundances. [file 13059_2022_2624_MOESM1_ESM.docx]

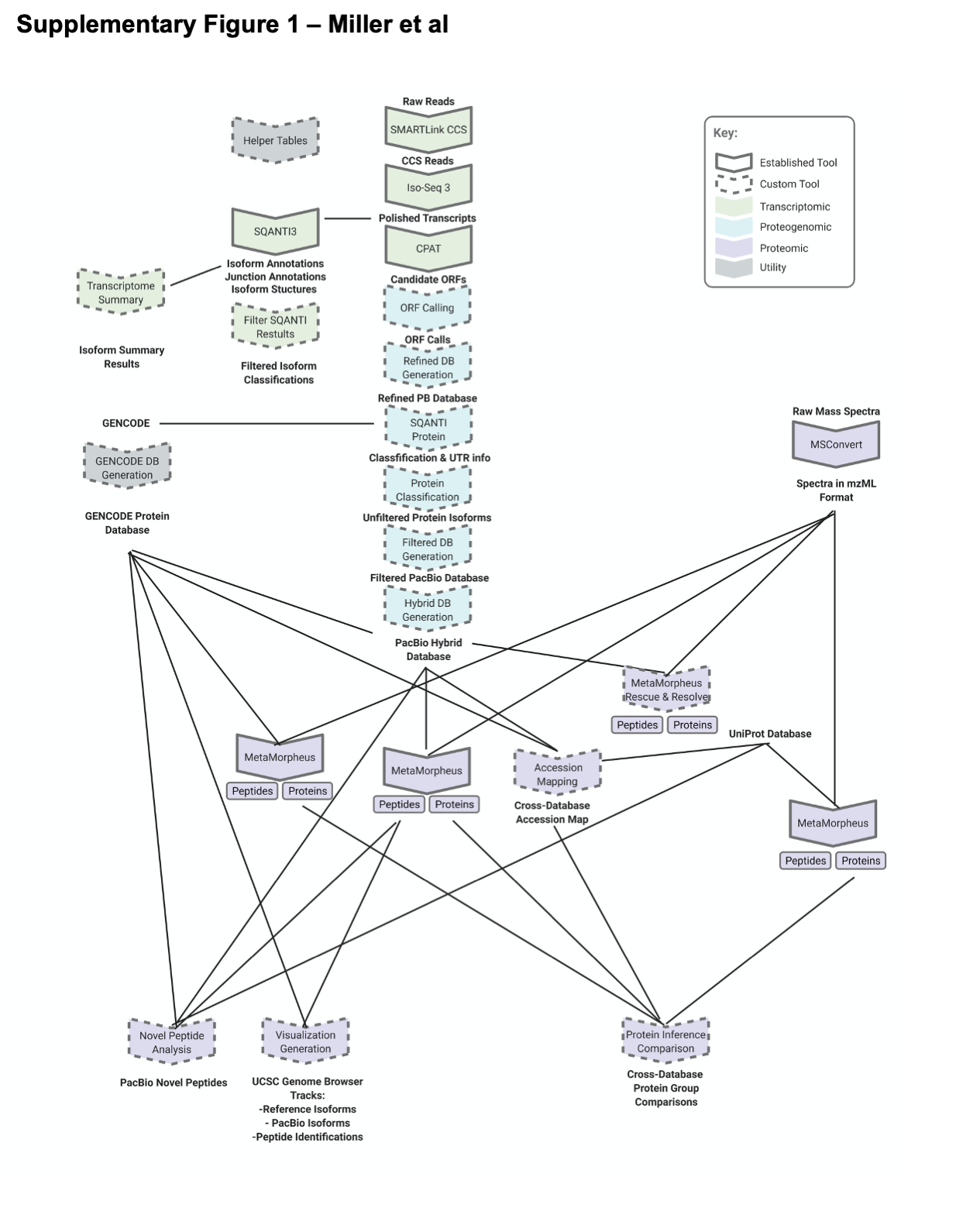


##### **Fig. S1: Detailed schematic of the Nextflow computational pipeline for long-read proteogenomics.** Computational pipeline for full-length protein database generation, database searching, and downstream data analysis and visualization. Complete details of the pipeline may be found at https://github.com/sheynkman-lab/Long-Read-Proteogenomics.

#####

##### **
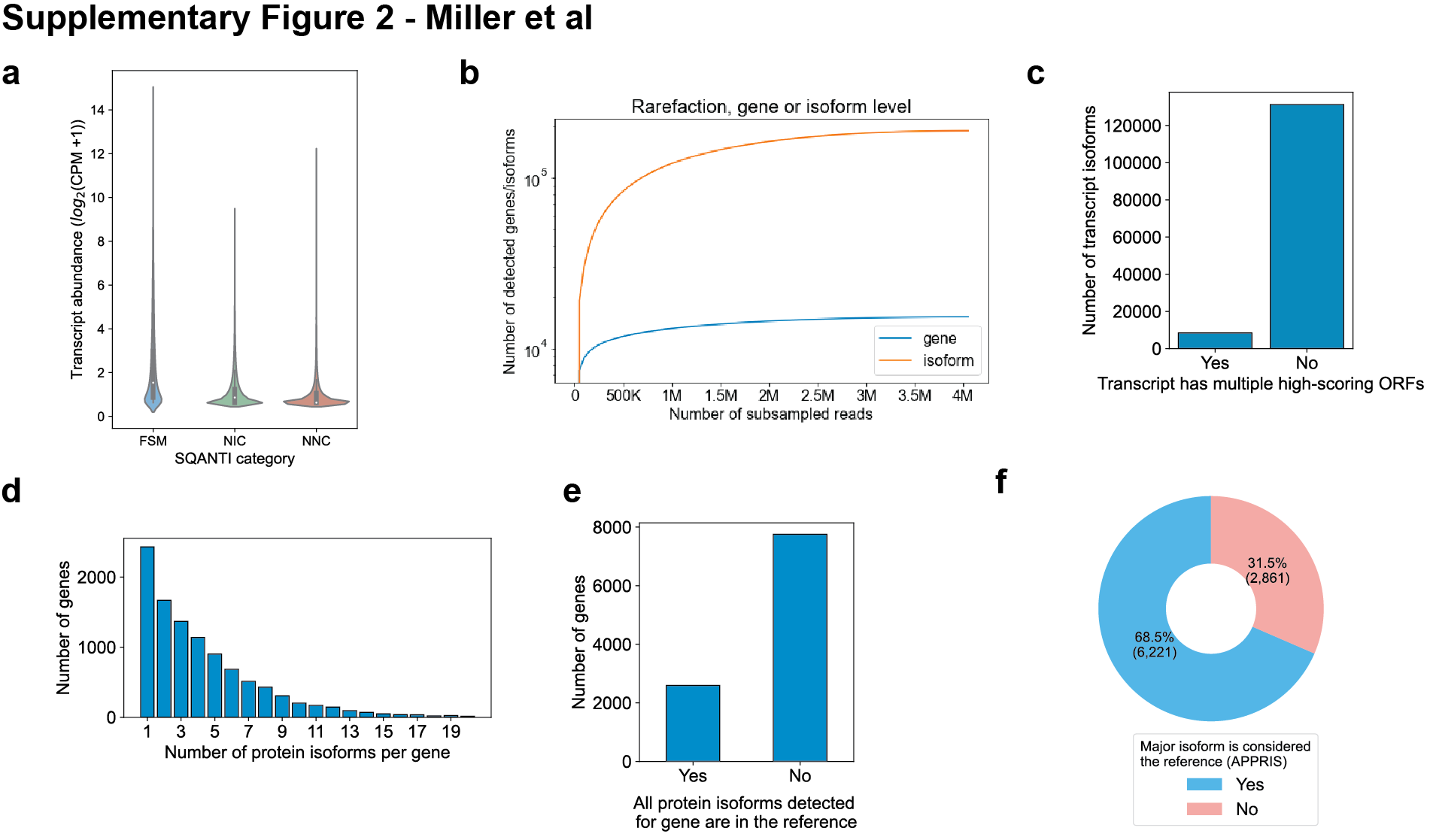
**

##### **Fig. S2:** **Generation and characterization of candidate protein isoform sequences from long-read RNA-seq data.** **a,** Transcript abundance distributions for known (FSM) versus novel transcript isoforms (NIC, NNC). **b,** Saturation-discovery curve to determine the relationship between number of full-length reads subsampled and total number of genes and isoforms detected. **c,** Count of transcript isoforms with more than one high scoring ORF (defined as CPAT score above 0.9). **d,** Distribution of the number of protein isoforms per gene. **e,** Bar chart showing the number of genes that contain all known protein isoforms. Data based on the filtered PacBio database (~45K protein isoforms). **f,** Fraction of genes for which the most abundant transcript underlying the protein isoform does not correspond to the APPRIS reference. ORF, open reading frame; CPAT, Coding-Potential Assessment Tool; FSM, full splice match; NIC, novel in catalog; NNC, novel not in catalog.


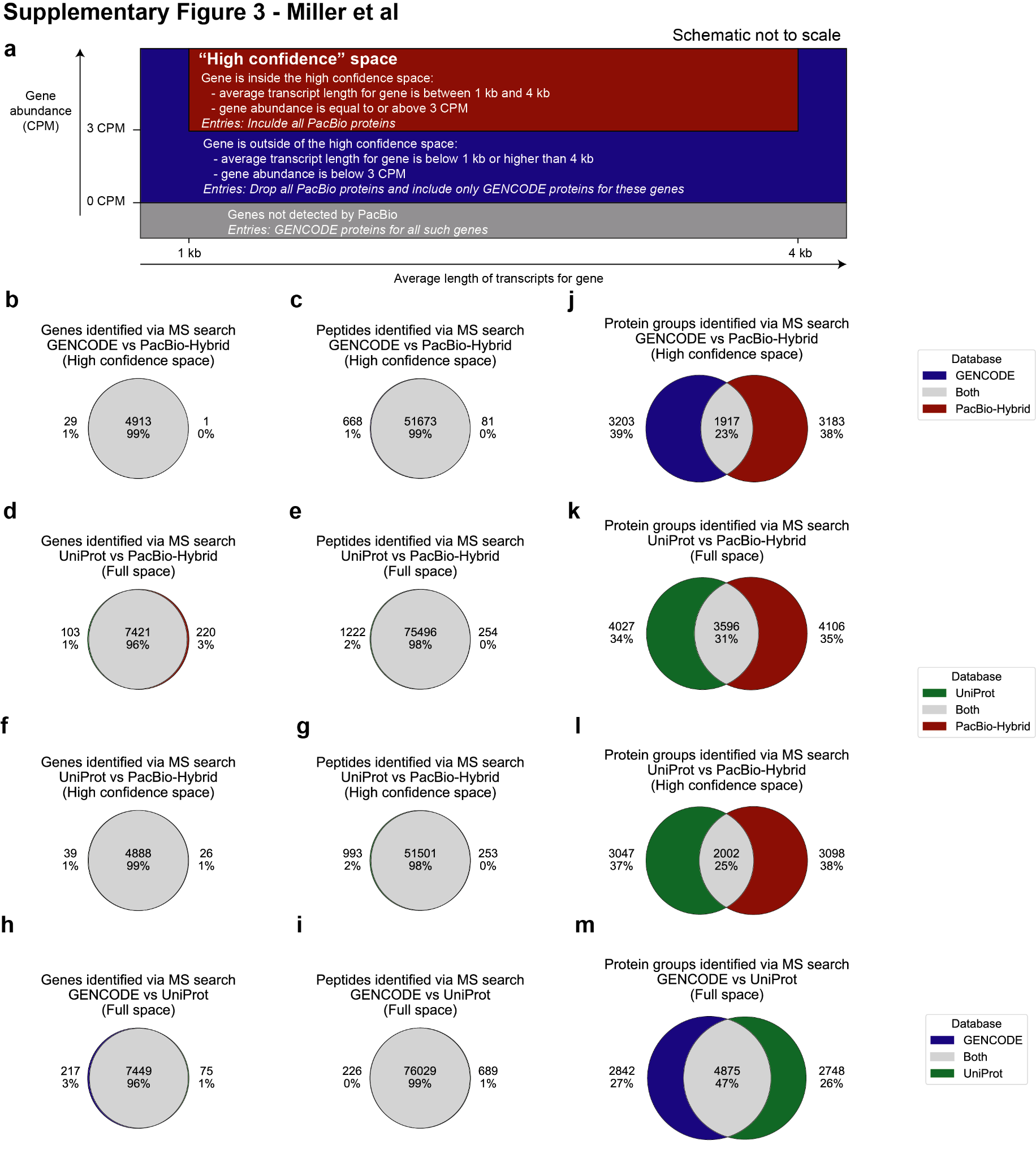


##### **Fig. S3: Comparison of MS-based proteomic coverage when using different protein databases for MS searching. a,** Schematic of the contents of the PacBio-Hybrid database (not to scale). **b-m** Overlap of gene, peptide, and protein group identifications when comparing GENCODE versus PacBio-Hybrid in the high confidence space (**b**, **c**, **j**), UniProt versus PacBio-Hybrid in the full gene space (**d**, **e**, **k**), UniProt versus PacBio-Hybrid in the high confidence gene space (**f**, **g**, **l**), and GENCODE versus UniProt in the full gene space (**h**, **i**, **m**).

#####
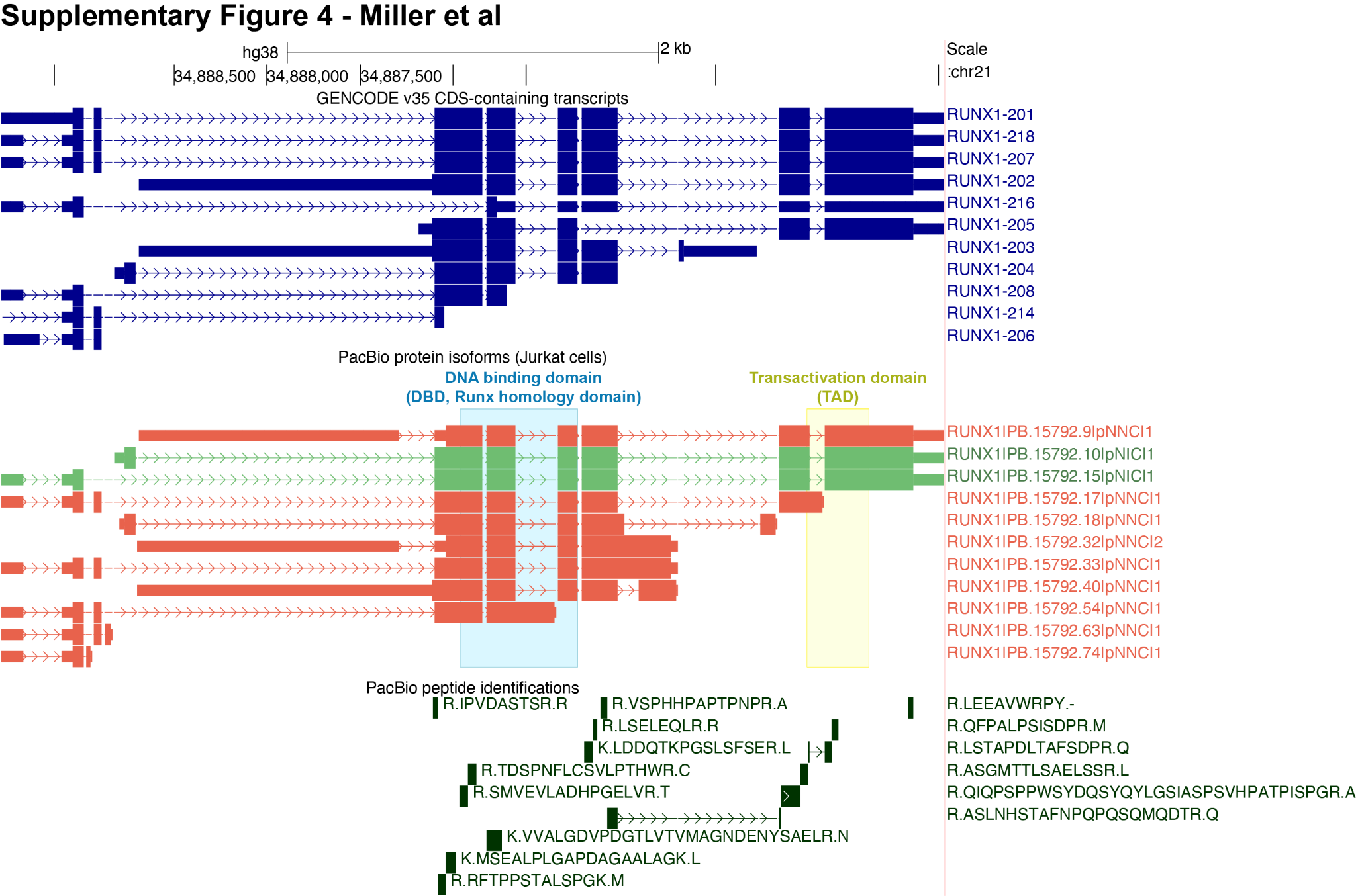


**Fig. S4: Novel isoforms detected for genes key to thymocyte tumor biology. a,** *RUNX1* isoforms in Jurkat cells are completely distinct from those in the GENCODE reference. Some *RUNX1* isoforms lack the transactivation domain and may act as a dominant negative. PB.15792.40 is one such isoform with a uniquely mapping peptide.

**
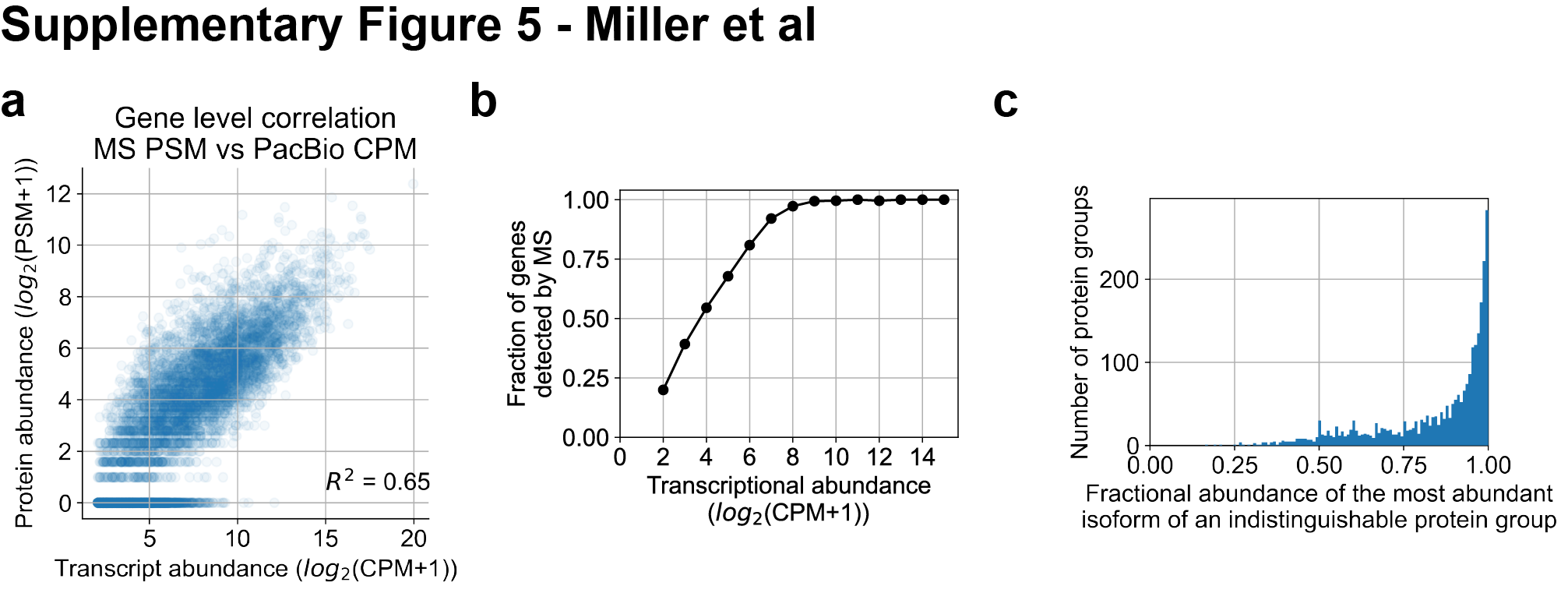
**

##### **Fig. S5: Relationship between RNA and protein estimated abundances. a,** Correlation between long-read transcriptional abundance and protein abundance. Results are grouped by gene. **b,** Fraction of genes detected by MS as a function of transcript abundance. **c,** Distribution of the fractional relative abundance of the most abundant protein isoform in each indistinguishable protein group.
