# Supplementary material for: Enhanced protein isoform characterization through long-read proteogenomics
Source: Genome Biol. 2022 Mar 3;23:69. doi: 10.1186/s13059-022-02624-y (PMC8892804; doi:10.1186/s13059-022-02624-y)
Supplement: Supplementary file 2 — Additional file 2: Notes S1-S5. Supplementary notes for the manuscript [31, 48, 71, 80–93]. [file 13059_2022_2624_MOESM2_ESM.docx]

# Supplementary Notes for Enhanced protein isoform characterization through long-read proteogenomics

### Note S1: Long-read transcriptome sequencing of a human cell line

We sequenced two cDNA libraries of the human Jurkat T-lymphocyte cell line each with SMRT Cell 8M on the PacBio Sequel II system and obtained a total of 5 million HiFi (CCS) reads with an average read length of 2.1 kbp. Following a standard Iso-Seq bioinformatics workflow (see **Methods**, <https://github.com/sheynkman-lab/Long-Read-Proteogenomics>), we classified and filtered the full-length transcript sequences, removing potential library artifacts.

Transcript isoform diversity is widespread in the sample. We identified 139,743 transcripts from 11,186 protein coding genes that exhibit a wide range of lengths and abundances (**Fig. S1.1**). Many genes express multiple isoforms, with some genes co-expressing up to a dozen or more isoforms, and 84% (8,367) of the genes exhibiting co-expression of more than two isoforms (**Fig. S1.2**).


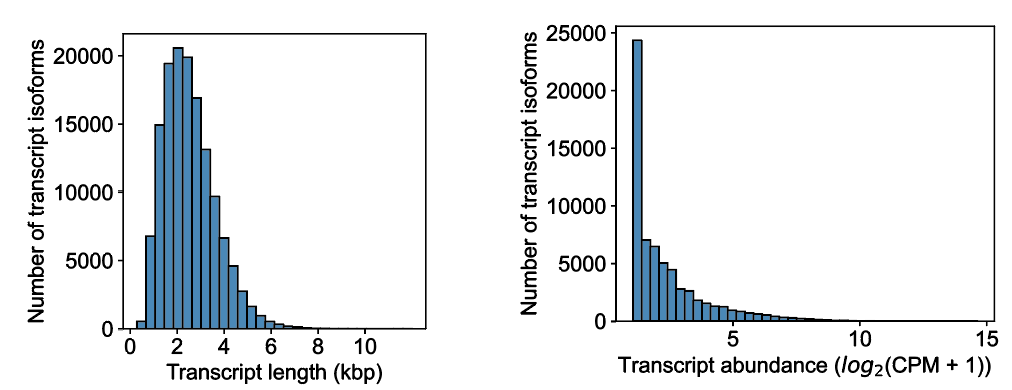


**Figure S1.1: Long-read transcriptome length and abundance distributions.** **(Left)** Distribution of transcript isoform lengths. **(Right)** Distribution of transcript isoform abundances. CPM, full-length read counts per million


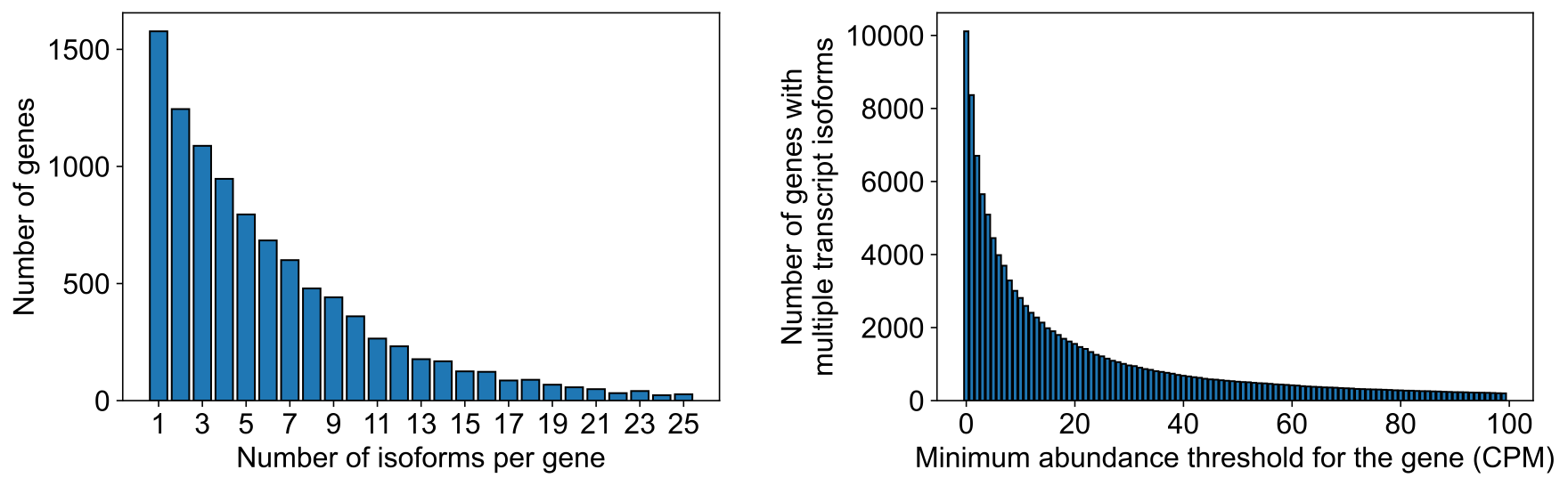


**Figure S1.2: Co-expression of multiple isoforms from the same gene.** **(Left)** Histogram of the number of distinct transcript isoforms per gene. **(Right)** Frequency of genes containing multiple isoforms, at different CPM abundance cut-offs. Only transcripts greater than 1 CPM were used for the data in these plots. CPM, full-length read counts per million.

For genes expressing multiple isoforms, we classified the corresponding isoforms as either major (i.e., most abundant isoform for a gene) or minor. Overall, minor isoforms tend to have lower abundance, but certain minor isoforms can still have robust expression and make up a large fraction of total gene expression (**Fig. S1.3**). For a substantial fraction of genes expressing multiple isoforms (38%, 3,888), the major isoform expressed in Jurkat cells was not the “reference” isoform (GENCODE APPRIS principal isoform[[31]](https://paperpile.com/c/12DD6P/W34RS), **Fig. S1.4**). Collectively, these results illustrate the widespread nature of alternative splicing and the need for empirically driven methods to characterize isoform diversity.


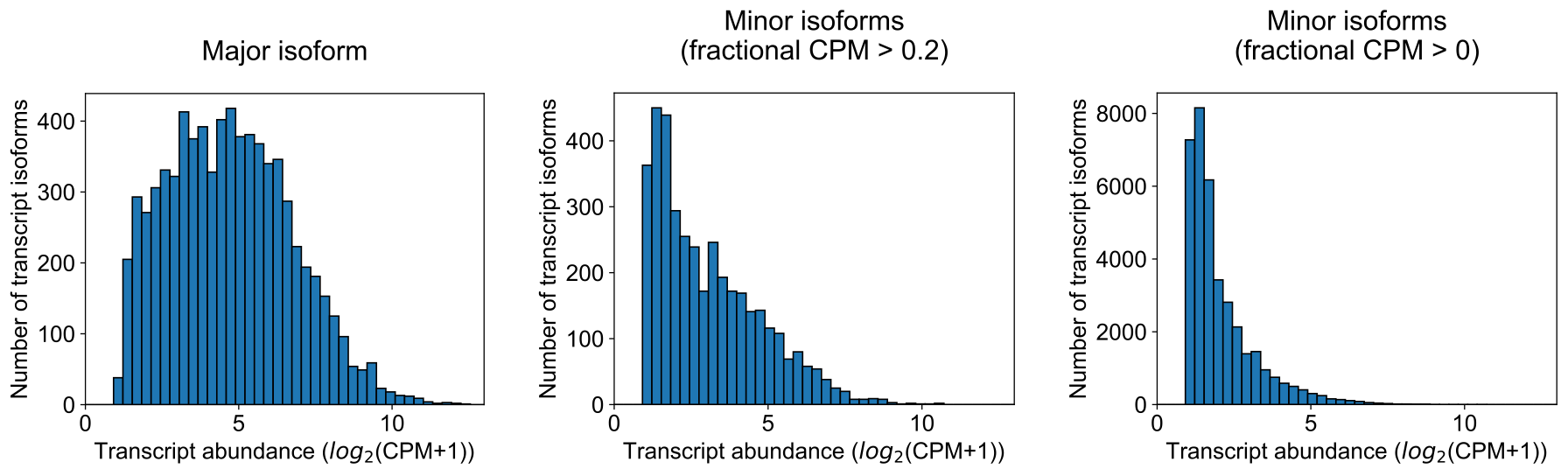


**Figure S1.3: Abundance distribution of major versus minor transcript isoforms. (Left)** Distribution of transcriptional abundance for major transcript isoforms. **(Middle)** Distribution of transcriptional abundance for minor transcript isoforms with a fractional abundance of more than 0.2. **(Right)** Histogram of transcriptional abundance for all minor transcript isoforms. Only transcripts greater than 1 CPM were used for the data in these plots. CPM, full-length read counts per million.


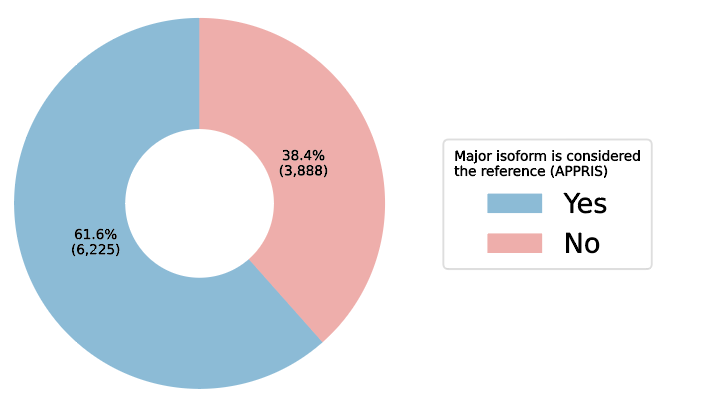


**Figure S1.4: Fraction of transcript isoforms in which the major isoform (highest isoform expressed for a gene, based on CPM values) does not match the GENCODE principle APPRIS transcript isoforms.**

Approximately 86% of the transcript isoforms were classified as “full-splice match” (FSM) or a novel category NIC or NNC (see **Main** text). The remaining 9,130 (14%) transcripts were classified as “incomplete splice match” (ISM) cases, which can result from partially degraded transcripts generated during sample and library preparation or, alternatively, represent *bona fide* novel alternative promoter or polyadenylation sites. As expected, minor isoforms tend to be novel at a higher rate than major isoforms (**Fig. S1.5**).


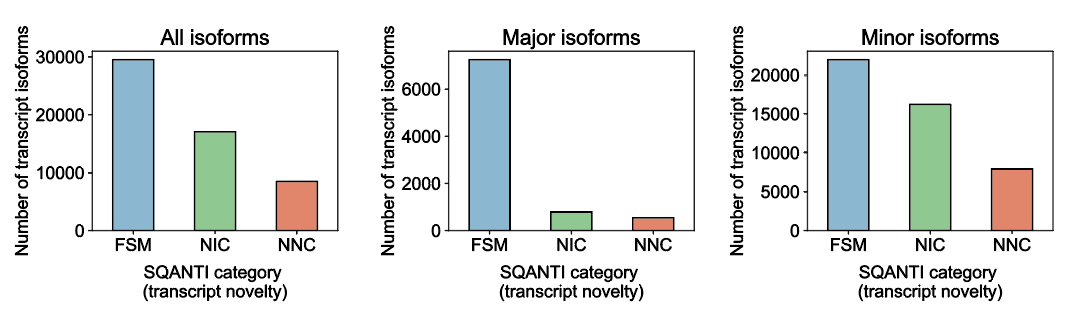


**Figure S1.5: Breakdown of transcript isoforms by their novelty category. (Left)** Number of transcript isoforms in each novelty classification category. **(Middle)** Breakdown of major (highest expressed for gene) transcript isoforms by novelty category. **(Right)** Breakdown of minor transcript isoforms by novelty category. Only transcripts greater than 1 CPM were used for the data in these plots.

### Note S2: ORF calling from long-read transcripts

For calling of ORFs from full-length transcript isoforms, we used the CPAT algorithm. Several ORF callers are available. We compared the identity of ORFs called using CPAT versus TransDecoder[[80]](https://paperpile.com/c/12DD6P/fAv0i) and GMST[[81]](https://paperpile.com/c/12DD6P/yzIsM). To run TransDecoder (version 5.5.0), a minimum ORF size was set to 50 nucleotides to mimic the parameters we used for CPAT. The single best ORF for each isoform was selected, per the `Transdecoder.Predict` parameter. To run GMST the same parameters as used in the SQANTI pipeline (GMST version 5.1) were used. We found that in a majority of cases, the same ORF is predicted; however, there are some differences in ORFs called.


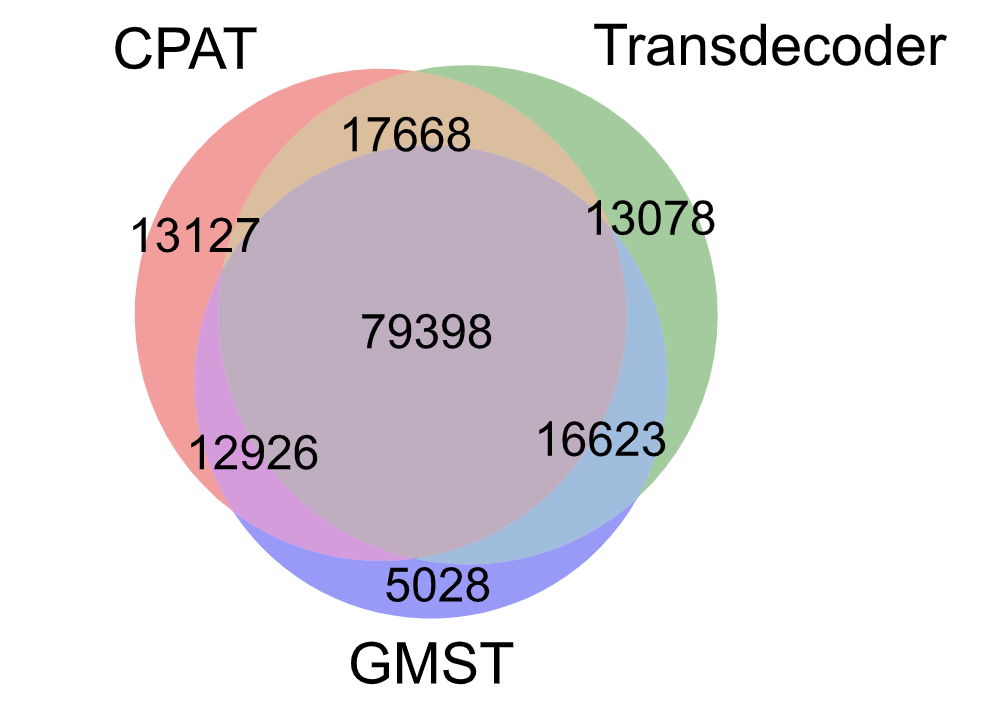


**Figure S2.1: Comparison of ORF callers in predicting ORFs from full-length transcripts (PacBio)**.

CPAT returns a coding score for each candidate ORF. Overall, the scores of the candidate ORFs form a bimodal distribution, and there is a clear distinction between high and low scoring ORFs, overall (**Fig. S2.2**).

In some cases, there are two or more ORFs that have a high coding score (from the CPAT algorithm). Generally speaking, the upstream-most ORF, containing an ATG closer to the 5’, was deemed more credible given the ribosomal scanning model of translation[[82]](https://paperpile.com/c/12DD6P/LA6r4). Therefore, we use this model as an assumption for ORF calling, in which higher weights are given to ORFs containing ATGs that are closer to the 5’ end of the transcript (**Fig. S2.3**). Implementation details about the ORF calling algorithm can be found in the `orf_calling` module in the Nextflow pipeline.


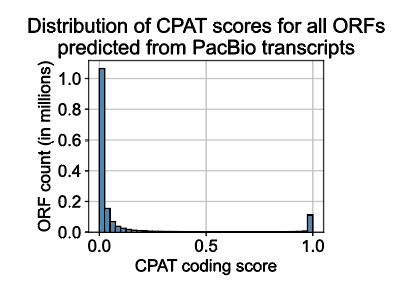


**Figure S2.2: Distribution of ORF scores from the CPAT algorithm**


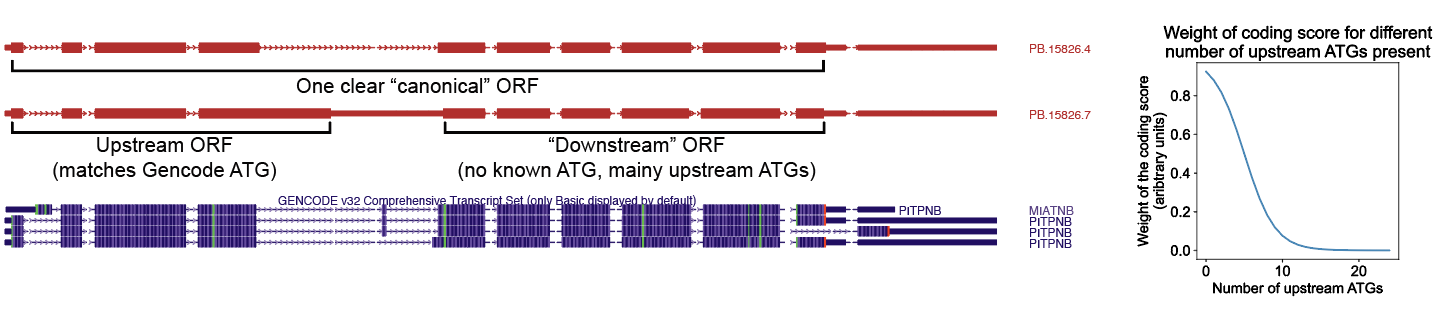


**Figure S2.3: (Left)** Example of two highly scoring ORFs from the same transcript called by CPAT for gene *PITPNB*. The top isoform PB.15826.4 contains one clear best ORF. The second isoform PB.15826.7 contains an extension in the 4^th^ exon, leading to a frameshift and premature termination codon. In such cases, the upstream-most ORF is the most plausible translated region. **(Right)** ORF score weighting based on the number of upstream ATGs.

In order to determine whether our ORF calling algorithm is able to recover ORFs annotated in GENCODE, we compared the GENCODE reference ORFs (ENSPs, i.e., GENCODE proteins) versus the *ab initio* CPAT-predicted ORFs. We found that of the 58,860 GENCODE transcripts, the CPAT-predicted ORF matched the ORF annotated by GENCODE in 55,324 or 94% of cases. For 3,536 or 6% of cases, the ORF was not an exact match (**Fig S2.4**). A majority of the ORFs that differ from the reference differ due to differences in the N-terminus, with 55% differing only in the ATG start location. Note that in our proteogenomics pipeline, as part of our ORF calling procedure, we heavily weigh the presence of a GENCODE ATG; therefore, a majority of these cases would be reverted to the GENCODE ORF within our pipeline. For 60 cases (0.1% of the dataset), only the C-terminus did not match, and a majority of such cases could be explained by selenocysteine recoding events, in which the stop codon is recoded to selenocysteine, thereby extending the protein C-terminus.


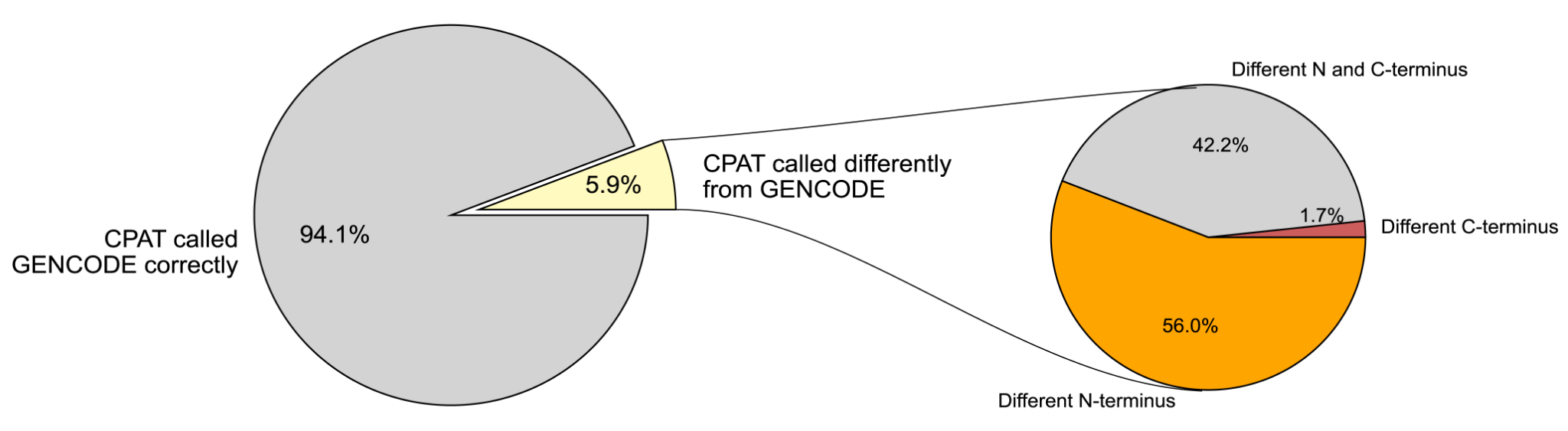


**Figure S2.4: Fraction of ORFs predicted from the GENCODE transcriptome using the modified CPAT ORF calling pipeline.** For predicted ORFs that did not exactly match GENCODE annotated ORFs, a breakdown of the part of the protein that differs (e.g., N-terminus) is indicated.

### Note S3: Determination of the high confidence protein database space based on long-read RNA-seq coverage of peptides

To determine factors underlying incomplete proteomic coverage using the PacBio database, we examined the properties of genes in which there were fewer peptides recovered when using the PacBio database than when using the GENCODE database. When searching the PacBio database (PacBio only), we detected 70,761 peptides and 7,068 genes, which corresponds to 90.7% of peptides and 92% of genes detected using the GENCODE database. Overall, lower peptide recovery was observed for genes with extreme transcript lengths (e.g., less than 1 kb, longer than 4 kb) or very low abundance (e.g., below ~3 CPM) (**Fig. S3.1**). The extremely short transcripts may not be sampled because the PacBio library preparation included a bead clean-up which removes short cDNAs. The longer transcripts are, in general, more difficult to convert to cDNA and sample for sequencing, although newer sequencing platforms may demonstrate less bias against lengths. Overall, as expected, the protein content of lower abundance genes or genes with extremes in lengths are not fully sampled in the long-read transcriptome dataset.


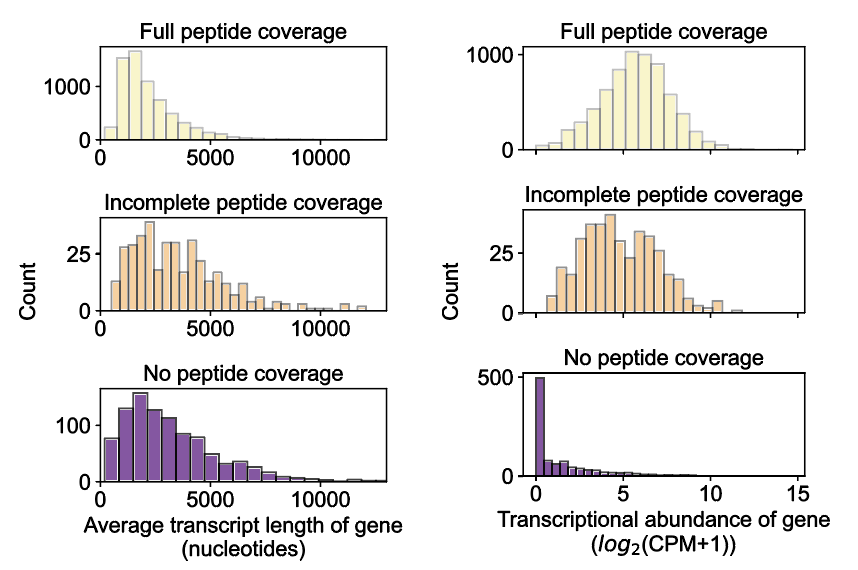


**Figure S3.1: Characterizing the length and abundance biases that contribute to lower proteomic coverage from PacBio-derived databases (Left)** Distribution of transcript lengths for genes with different extents of peptide coverage. **(Right)** Distribution of transcript abundances for genes with different extents of peptide coverage.

A majority of genes detected using the GENCODE database also returned 100% peptide coverage when using the PacBio database, suggesting long-read datasets are reaching a critical threshold of coverage capturing the full sequence content of protein-coding mRNA. In other words, given reasonable constraints (length 1-4 kb, abundance 3 CPM+), we achieved nearly 99% coverage of the known peptide sequence space (**Fig. S3.2**). For this set of high confidence genes, it is likely that all expressed protein isoforms are represented in the long-read data, including novel isoforms not represented in the reference database, which is advantageous for protein inference.

**
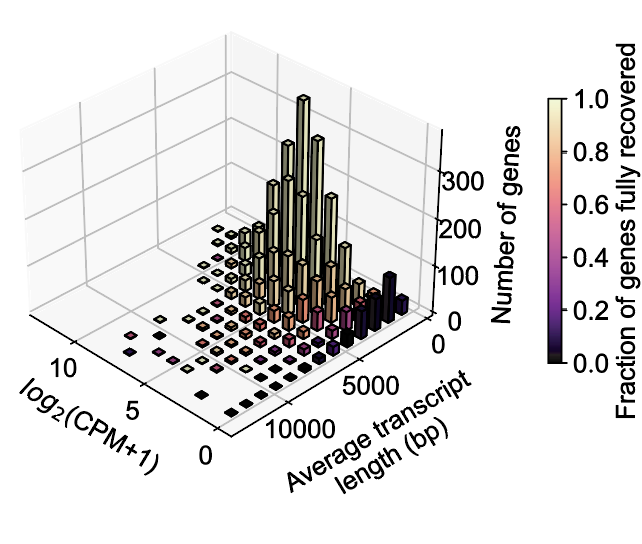
**

**Figure S3.2: Three-dimensional bar plot that displays the fraction of genes in which all peptides are recovered in the PacBio database, as a function of gene average transcript length and abundance.** CPM, full-length read counts per million.

### Note S4: Criteria for Novel Peptide Identification

The identification of novel peptides requires rigorous validation and stringent filtering criteria to ensure that the spectrum does in fact represent the novel peptide sequence. First, all novel peptide identifications must meet the basic filtering criteria for all peptide identifications, having a minimum MetaMorpheus score of 5 and being present at a global 1% FDR cutoff. The distribution of the FDR, or q-values, for the novel peptide identifications was compared to the FDR, or q-value, distribution of the canonical peptides (**Fig S4.1**). The median q-value for the novel peptides was lower than the median q-value for the canonical peptides with values of 0 and 2.1x10^-5^, respectively. Since the FDR, or q-value, is a global assessment of confidence for the entire set of peptide identifications, not a confidence metric for the specific peptide identification, the posterior error probability (PEP) of each novel peptide was also considered. All novel peptides had a PEP value less than 0.005. The distribution of novel peptide PEP values was compared to the distribution of PEP values for all canonical peptide identifications (**Fig S4.1**). The median PEP value for the novel peptides and canonical peptides were nearly identical, demonstrating the novel peptide identifications are as confident as the canonical peptide identifications.

In addition to the preliminary filtering and confidence evaluations of the novel peptide identifications, the Human Proteome Project MS data guidelines[[83]](https://paperpile.com/c/12DD6P/VAcD) for manual validation of data-dependent acquisition spectra were applied. These criteria include: 1) high mass accuracy (5 ppm precursor ion and 20 ppm product ion mass error), 2) clearly annotated spectrum that was scrutinized for missed and extra peaks (we used a rough threshold of 25% maximum of unassigned fragment ions above 10% relative abundance) and 3) peptide length (minimum of 9 amino acids).


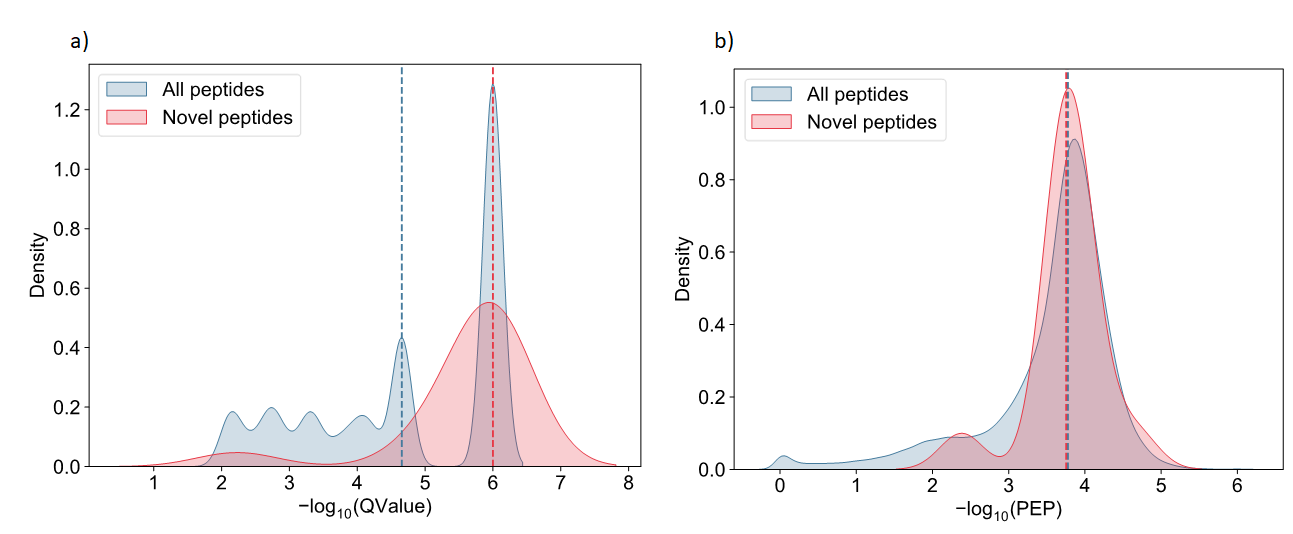


**Figure S4.1: Comparison of novel and canonical peptide distributions for (left) q-values and (right) PEP values.** The median value is represented by a dashed line.

Additional manual validation criteria were applied to each novel peptide identification. Mass errors of novel peptide hits were compared against the mass error of other confident spectral assignments from the same raw file to check for consistency. Extra weight was given for the presence of sequence-specific characteristics. For example, the spectra assignment was considered more confident if a highly charged peptide was longer, had a higher number of basic residues; or if y-ions ending in proline were higher intensity than other fragment ions. Precursor co-isolation can complicate spectral annotation by resulting in fragment ions from multiple peptide origins. This complexity was taken into account by noting the percentage of MS2 total ion count (TIC) accounted for by annotated fragment ions. This percentage is represented by the decimal digits of the MetaMorpheus score. TIC coverage of 20% or more was used as a rough threshold but not a hard criterion.

We also investigated whether any peptide sequences from contaminant proteins, GENCODE reference isoforms, single amino acid variant containing proteins or post-translationally modified proteins could provide better peptide assignments for the novel peptide spectra. Identifications were evaluated based on MetaMorpheus score, q-value and PEP. A contaminant protein database, included in MetaMorpheus, was searched alongside the PacBio-Hybrid database. No contaminant peptides were a match to the novel peptide spectra. In the search of the GENCODE reference database, no peptide identifications for the spectra in question were a better match than the novel peptides (**Additional File 6:** **Table S4**). To search for post-translationally modified peptides, Global Post-Translational Modification Discovery (GPTMD), with default settings, was performed with the GENCODE reference database [[71]](https://paperpile.com/c/12DD6P/b4fBH). The subsequent search found no modified peptides were a match for the novel peptide spectra. To investigate if variant containing peptides could better account for the spectra supporting the novel peptide identifications, a proteogenomic database generated by Spritz using Jurkat short-read RNA-seq data published by Cesnik et. al. [[84]](https://paperpile.com/c/12DD6P/k53wU) was searched. The results showed that no variant containing peptides were identified for the same spectra as the novel peptide assignments. Based on this stringent evaluation of novel peptide candidates, we are quite confident in the 14 novel peptide assignments that passed all criteria.

### Note S5: Rescue & Resolve algorithm abundance threshold optimization

The R&R algorithm requires selection of a transcript abundance threshold that is the basis for recovering a formerly eliminated protein identification. If the transcript abundance threshold is set too low, there is a higher probability of recovering protein isoforms that are not expressed (false positives). If the threshold is too high, there is a higher probability of failing to rescue protein isoforms present in the sample (false negatives).

We evaluated 10 different abundance threshold values (5, 10, 15, 20, 25, 30, 35, 40, 45 and 50 CPM) for the R&R algorithm. As expected, the lower the CPM abundance threshold, the more protein groups are rescued (**Fig. S5.1)**, however these larger values are not necessarily indicative of a higher true positive rate.

Protein inference results obtained from searching a higher coverage MS dataset (i.e., a multi-protease proteomics dataset) can be utilized as a validation group. We can compare the rescued protein groups from the 28-fraction trypsin-only set to the protein inference results derived from a high coverage multi-protease digest data set, which serves as a “ground” truth set, to obtain a percent of rescued protein groups validated (see **Note S6**). We found that as the abundance threshold increases, the percent of rescued groups validated in the multi-protease protein inference results increase (**Fig. S5.1**). Based on this analysis we decided to set a conservative threshold to rescue transcripts with an abundance of 25 CPM or higher. Additionally, based on observed relationships between transcript abundances for genes with and without peptide evidence (**Fig. S5.2**), with a CPM cutoff of 25 (log2 (CPM+1) = 4.6) only 9.7% of genes in the high confidence space have no peptide support.


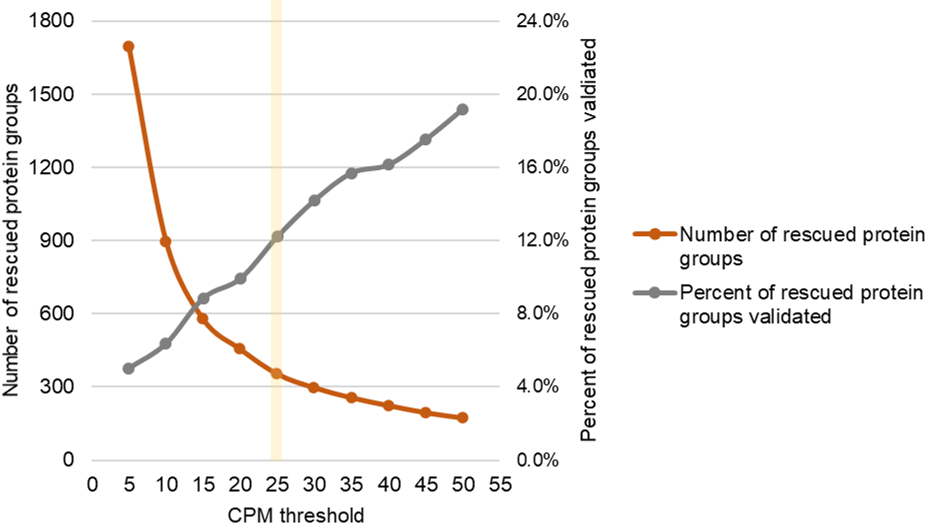


**Figure S5.1: Abundance threshold evaluation for the Rescue & Resolve algorithm.** The orange curve represents the number of protein groups rescued at 1% FDR for each CPM abundance evaluated. The grey curve represents the percent of rescued protein groups whose identity was validated in an independent multi-protease MS dataset.


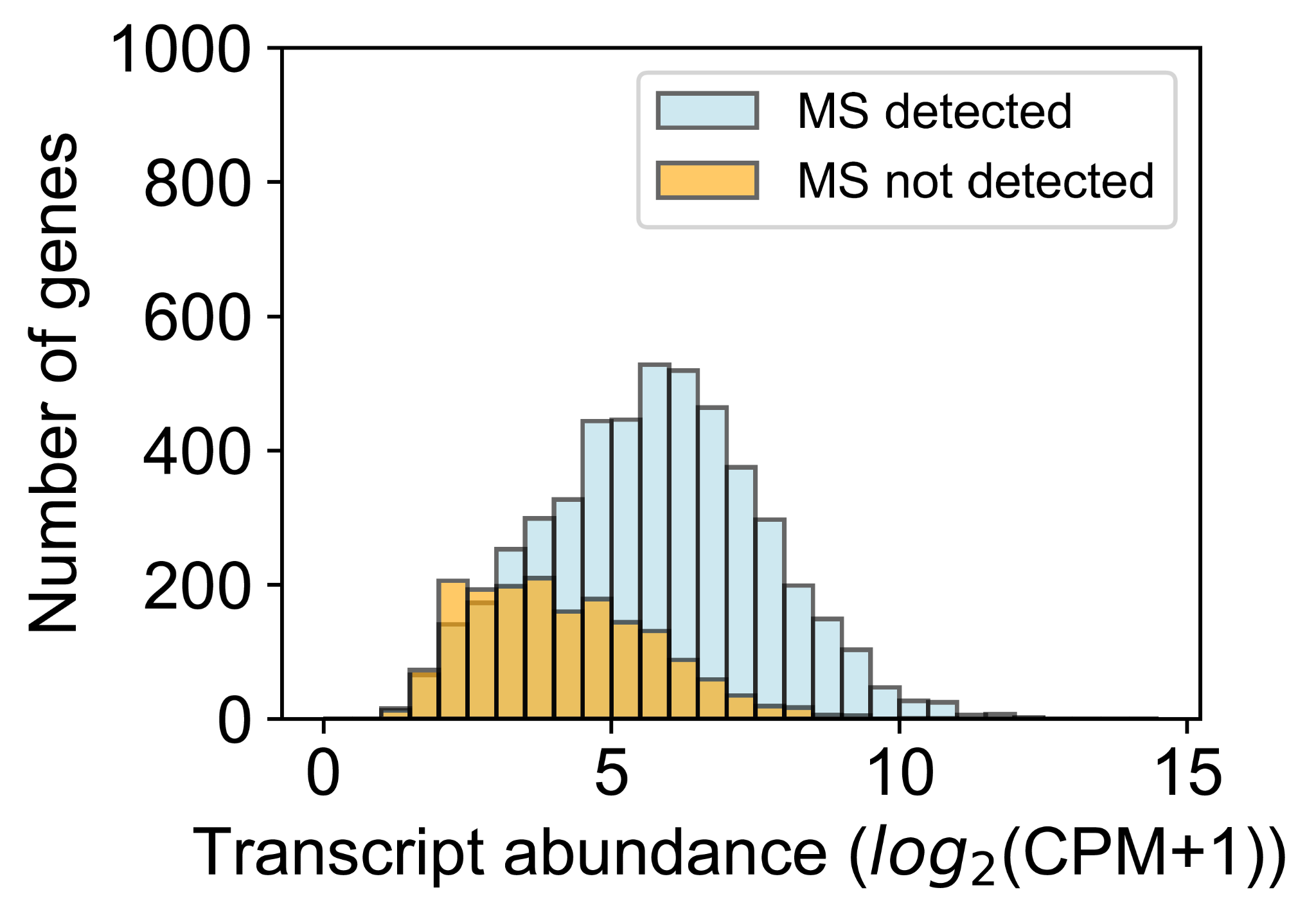


##### **Figure S5.2: Relationship between transcriptional abundance and MS detectability.** Distribution of genes either detected or not detected by MS as a function of cumulative transcriptional abundance.

### Note S6: Multi-protease validation for Rescue & Resolve results

We hypothesize the R&R algorithm enables a more precise representation of sample isoform diversity than can be achieved using a traditional protein inference approach. However, the experimental validation of protein isoforms inferred from MS data is an ongoing challenge[[85, 86]](https://paperpile.com/c/12DD6P/8LtJK+2WhLa). Existing analytical standards fail to appropriately model the complexity of endogenous isoforms[[87, 88]](https://paperpile.com/c/12DD6P/7RL7a+strxX), and the lack of knowledge regarding protein isoforms present in a sample (i.e., ground truth) is a problem in and of itself[[89-91]](https://paperpile.com/c/12DD6P/8tOga+xaPZb+9dyO6). In place of experimental validation, studies have established heuristic guidelines to compare protein inference results between different inference algorithms[[85, 92]](https://paperpile.com/c/12DD6P/8LtJK+JRtik). Here we have developed a computational strategy that leverages the improved accuracy and precision of multi-protease protein inference to validate both the “rescued” and “resolved” protein isoforms.

It is well established that the use of multiple proteases improves proteomic results relative to those of a single protease (typically trypsin)[[48, 93]](https://paperpile.com/c/12DD6P/qO0RK+9vfZk). Large portions of the proteome are inaccessible to any single protease, and the combination of peptide identifications from orthogonal proteolytic digests not only improves the number of protein identifications but also the percent protein sequence coverage obtained[[48, 93]](https://paperpile.com/c/12DD6P/qO0RK+9vfZk). The MetaMorpheus MS search software contains a multi-protease protein inference algorithm which enables all peptide identifications from several proteolytic digests to be considered in combination, and provides more accurate protein inference results than what is achieved by other protein inference algorithms, or by analysis of any single proteases data alone[[48]](https://paperpile.com/c/12DD6P/qO0RK).

For validation of “rescued” and “resolved” protein isoforms, an independently generated multi-protease dataset was used. Spectra from the analysis of six fractionated proteolytic digests (see **Methods**) were searched against the PacBio-Hybrid database using MetaMorpheus, and the multi-protease protein inference algorithm was employed[[48]](https://paperpile.com/c/12DD6P/qO0RK). The multi-protease protein inference results are considered to be more comprehensive, and reflective of the sample’s proteome compared to what can be achieved with trypsin alone. The use of orthogonal proteases provides higher, and more distinctive coverage of protein isoforms. One product of this is the identification of more unique peptides, which can confidently identify protein isoforms. Although the multi-protease protein inference results are not a perfect model of the isoforms expressed in the sample, for the purpose of our validation strategy, we will consider the results as a “ground truth” dataset.

For the purposes of validation, we determined if the protein isoforms that were “rescued” or “resolved” by “Rescue & Resolve” algorithm were isoforms that were identified in the multi-protease protein inference analysis. If a “rescued” or “resolved” protein isoform was identified—in the multi-protease analysis—as a single protein isoform, not as part of a multi-isoform protein group, the identification of the protein isoform in question was considered to be confirmed, or “validated”. These validated “rescued” or “resolved” isoforms, if identified in the multi-protease protein inference results, had sufficient peptide level evidence, such as an isoform-specific peptide, due to the identification of additional non-tryptic peptides derived from orthogonal proteases to support their confident identification. The percent of “rescued” and “resolved” protein isoforms whose presence were confirmed, or “validated”, in the multi-protease protein inference results can be calculated. This percent validation rate, when compared to expected rates, indicates how well the “rescue” and “resolve” portions of the “R&R” algorithm do at increasing the number of true positive protein isoform identifications.

Since the multi-protease protein inference results are still incomplete and subject to error due to incomplete peptide coverage of the proteome, the percent validated means very little on its own. To assess the significance of the percent of “rescued” or “resolved” isoforms validated, the experimentally determined value can be compared against the validation rates expected at random. Such values can be computed by calculating the percent of protein isoforms validated for a pool of randomly “rescued” or “resolved" protein isoforms.

For the evaluation of the “rescue” portion of the “Rescue & Resolve” algorithm we compared the rate of validation between the “rescued” isoforms and randomly selected isoforms (background null). We “rescued” 355 isoforms based on additional transcriptional evidence, from a pool of 15,700 isoforms that represent all the protein isoforms that could possibly be “rescued”. The same number of protein isoforms that were rescued in the experimental results (N=355), were randomly selected, agnostic of transcriptional abundance, from the pool of 15,700 protein isoforms that were discarded in the protein inference process. Once the randomly “rescued” isoforms have been selected, we determined if such protein isoforms were identified in the multi-protease protein inference results, and the percent of isoforms validated was calculated just as was done for the experimental results. This process of randomly selecting 355 protein isoforms to “rescue” and determining the validation rate was repeated for a total of 1,000 permutations to generate a null distribution of validation rates against which the experimentally obtained results were compared, and statistical significance with a *p*-value <0.0001 was determined.

For the evaluation of the “resolve” portion of the “Rescue & Resolve” algorithm, a random protein isoform was selected as the dominant isoform, or “resolved” isoform, for each of the 1,434 protein groups that were resolved to a single, dominant, protein isoform in the experimental results. The randomly “resolved” isoforms were compared to the protein isoforms identified in the multi-protease protein inference results and the percent of isoforms validated was determined. This process of randomly selecting a single isoform for each resolvable protein group was repeated for a total of 1,000 permutations to generate a null distribution of validation rates against which the experimentally obtained results were compared and the statistical significance with a *p*-value <0.0001 was determined. For this permutation analysis, the chance of randomly selecting the dominant protein isoform that was resolved experimentally was much higher due to a smaller pool of candidates for random selection, which included a mixture of isoforms that were “resolved” (deemed expressed) mixed in with isoforms which were discarded (deemed not expressed, based on transcriptional evidence). Because of this an additional experiment was conducted, in which all the minor protein isoforms that were removed during the resolution of protein groups were compared against the obtained multi-protease protein inference results. This provides a percent validation rate for all of the isoforms removed during the experimental resolution of protein groups. This value was also compared to the null distribution for statistical significance with a *p*-value <0.0001.
