# Supplementary material for: Enhanced protein isoform characterization through long-read proteogenomics
Source: Genome Biol. 2022 Mar 3;23:69. doi: 10.1186/s13059-022-02624-y (PMC8892804; doi:10.1186/s13059-022-02624-y)
Supplement: Supplementary file 9 — Additional file 9. Review history. [file 13059_2022_2624_MOESM9_ESM.docx]

**Review History**

**First round of review**

**Reviewer 1**

**Are you able to assess all statistics in the manuscript, including the appropriateness of statistical tests used?**

Yes.

**Comments to author:**

The detection of translated protein isoform using mass spectrometry remains challenging. Here the authors explored the feasibility of using long-read PacBio sequencing to improve protein isoform inference in shotgun proteomics. A major result here is that full length isoform transcripts in Jurkat cells can significantly differ from GENCODE reference transcripts, which will have important implications for isoform inference and proteogenomics studies. A protein inference scheme and software pipeline is also provided for use. Overall this looks like a promising study that will be of interest to other researchers employing proteogenomics methods, but I have some comments regarding the implementation and benchmarking in their current form.

Major comments 
1. Although the title and central message of the paper suggest that long read sequencing enhances isoform identification, the results appear to only showcase that it is *feasible* to use long read sequencing to identify isoforms. Was there any attempt to compare with common existing workflows using short read sequencing to show practical improvement in isoform detection? This could be done for instance using public Jurkat cell Illumina sequencing data and current in silico translation software packages like CustomProDB and others.

2. Based on some of the examples given, e.g., LNPK-201 vs. LNPK-212; and MECP2-205 vs. MCEP-201 (pg.12 line 25) it is hard to see immediately what benefits do the proteogenomic and ORF finding workflow bring. It appears that the PacBio data mainly show that among two LNPK GENCODE transcripts (LNPK-201 vs. LNPK-212), the 201 isoform is more likely to be truly expressed; and that likewise among MECP2-205 and MECP2-201, 205 is expressed but not 201.
a. What would be the outcome if the authors simply searched the MS data with the GENCODE database then annotated the result based on PacBio read count data?
b. Secondly, did the authors investigate whether short-read sequencing data followed by transcript assembly would have been able to tell apart the difference between LNPK-201 and LNPK-212?

3. The authors provide a new inference algorithm "Rescue & Resolve" here, but the exact contribution of the algorithm to improving protein isofrom identification is not clearly described in the manuscript. One would assume that the use of long read sequencing data and full-length transcript assembly by itself already resolved many of the most challenging inference problems since fewer isoform transcripts are present in the database. Does the algorithm also work with the more common short read assembled databases?

4. Related to the above, the manuscript should better clarify what the Rescue & Resolve algorithm does. As best as I could tell, it seems be that whenever there is a peptide identified to a protein group containing two transcripts and if the long read abundance of one of those two transcripts is responsible for over 90% of the transcript reads then the algorithm assumes the higher abundance transcript is uniquely found and disregards the lesser isoform.
a. What current protein inference algorithm is R&R being compared against?
b. Logically it would appear to me that one cannot assume that the isoform peptides come from only the higher abundance isoform. For instance, 10% of the peptides might feasibly come from the lower protein isoforms with 10% read abundance. Conversely even if two isoforms are found in the PacBio data with >30% relative abundance, they could have different translation potential and only one could make a stable protein product so without empirical evidence of a unique peptide one couldn't just assume both are translated. Isn't this the reason to perform proteo(geno)mics experiments to begin with, since one cannot assume the transcript is translated?
c. Existing software like MaxQuant already report total protein groups identified, so the present method would seem to not so much as improve protein IDs than to provide a way to annotate redundant protein groups. In this case one might argue whether the algorithm is needed beyond simply applying a read count criterion when creating the protein database.
d. If the lesser transcript is at all found within the long-read sequencing data, it would seem to me that true protein isoform inference would require isoform assembly at the protein level through probabilistic modeling. I would suggest more clearly describing R&R as a heuristic filter as the authors mentioned in page 20, rather than an inference algorithm throughout the manuscript.
e. Please also clarify what the permutation test on page 17 is testing for. What is being permuted, e.g., are the labels being swapped randomly for the relative read proportion within a protein group, or do the authors redraw from the distribution of the entire population of proteins/isoforms etc.
f. The performance of the inference is hard to verify currently and this should be made more clear. The multi-protease dataset doesn't necessarily provide a gold standard as more abundant transcripts are more likely to be found in the multi-protease mass spec data due to their detectability. The more important question here is whether the "resolved" transcript that gets removed is truly absent and how might one validate that and establish ground truth. Is there a difference in inference performance based on total transcript abundance since presumably for those genes even the transcript with lower read proportion would be more likely detectable?

5. Page 12 states that "protein isoform groups distinct to the protein inference results account for 382 cases of differing protein group composition.
a. I would suggest rewording this sentence for clarity. At the moment it is difficult to understand what the authors mean. Do they mean there are 382 instances where the incorporation of PacBio data led to a resolution of unresolved protein groups in GENCODE?
b. How many of these are found through unique isoform peptides not mappable to GENCODE, and how do these results correspond to the later section (page 14) that found 16 novel peptides?

6. In Figure 3d, how many novel isoforms are in the 69% genes within 'partial overlap' category, and what is the percentage of genes in belong to the "Superset" and "Distinct" isoforms from PacBio data categories? From the descriptions in the manuscript one might have assumed that there many pNNC events containing novel isoforms not in catalog (pg. 8 line 45) and hence presumably contain novel coding exons, but the protein ID results show that the peptide IDs are largely the same between GENCODE and PacBio. Does that mean none of those 20000+ pNNC events code for proteins?

7. As the authors pointed out, "Stringent validation criteria must be applied for novel peptide identifications" (page 14 line 19) but the criteria for such validation are not well defined in the manuscript and only manual examination is mentioned. Manual examination by trained experts can of course give some indication of how well the PSM is in terms of number of matched and unmatched peaks and peak series, but one would assume it does not give an indication of how good the peptide-spectrum match in question is when compared to other potential sequence matches in the databases.
a. Please clarify what were the predefined criteria for manual validation and whether any blinding was involved.
b. How do the q values/FDR of these matches compared to the q value distributions of canonical peptide PSMs?
c. Since FDR in proteomics typically refers to a property of the entire set of admitted results, did the authors look at the local FDR or posterior error probability of the individual peptide spectrum matches?
d. Were post processing algorithms like Percolator or Peptide Prophet used to authenticate and/or re-rank the PSMs?
e. What were the isoform PSM spectra matched to in a regular database search and how did their corresponding q values compared to searching with the Proteogenomics database?
f. Did the authors examine whether the spectra could be explained by other means like SAAV, contaminant proteins from other species, or unknown PTMs (as discussed in Neshvizhskii Nat Methods 2014 and elsewhere)?
g. Some of the peptides (GELDGELR and ELAGEAR) are relatively short and so carry higher chance of misidentification. I would strongly recommend the authors adhere to the HPP guidelines of at least 9 amino acids for "extraordinary detection claims" of novel peptides (Deutsch et al. J Proteome Res 2016) and disregard them from consideration.

8. I was somewhat surprised that only a few unique novel peptides were detected (16 peptides), this seems to run counter to the results of some existing short-read sequencing based proteogenomics studies.
a. What is the depth of the mass spec experiments and the predicted percentage of peptides that were mappable to non-canonical isoforms, regardless of whether they exist in GENCODE?
b. Currently the manuscript only mentions in passing "Many of these proteomic variations are difficult to identify from short-read RNA-seq datasets". As already suggested above, I would recommend the authors actually compare with a current proteogenomics workflow, i.e., using a database generated by short-read sequencing, to contrast the number isoform peptides identifiable in the mass spec data, which would allow the authors to determine whether there is improvement over existing workflows or alternatively attempt a "post-mortem" analysis of where some of the isoforms are lost.

9. The description on page 15 would seem to fall short of a timely overview of the existing start of the art in protein inference algorithms. ProteinProphet for example allows sister peptide features to be turned off and hence a unique peptide should lead to a unique protein IDs, whereas other inference algorithms such as implemented in Percolator/Fido would directly assess the probability that a protein can emit an observed PSM.

10. How much of the non-overlap between GENCODE and PacBio transcripts can be attributable to the limitations of the CPAT ORF prediction algorithm, for example not able to find alternative N termini or translation starts?

Minor comments:

11. Figure 1 looks somewhat confusing to this reviewer. In Figure panels 1b-1f, one assumes the authors meant for the blue and red boxes to refer to Isoform sequences in reference database vs. isoform sequences in sample-specific databases, as the actual protein isoforms in a sample may differ from both. Some of the "consequence" of using reference vs. sample-specific database should be better clarified, for example, because sequencing sensitivity and specificity are not perfect, when the sample-specific database is a subset of the reference database the identified specific isoform may be a false positive.

12. Given the major finding here is the differences between GENCODe and PacBio-hybrid transcripts rather than identification of novel peptide, one wonders if "enhancing protein isoform inference using long-read sequencing data" would make for a more accurate title for the manuscript.

**Reviewer 2**

**Are you able to assess all statistics in the manuscript, including the appropriateness of statistical tests used?**

Yes

**Comments to author:**

This manuscript "Enhanced protein isoform characterization through long-read proteogenomics" reports a new method for the incorporation of long-read DNA sequencing for transcriptome and proteome analysis. This is an important problem. The approach is novel and carefully developed. The manuscript is well written, but can be improved to clarify the performance of the method.


1) The manuscript uses PacBio sequencing of Jurkat cells. It is generally agreed and established from prior studies that long-read sequencing is superior than short-read (Illumina) sequencing for transcript and isoform detection. However, it is not clear whether the current data of Jurkat cells are sufficiently sampled to detect most expressed transcripts. The data can be sub-sampled to show the dependency of unique transcript detection on sequencing converge. This would be important to demonstrate adequate sensitivity.

2) It is known that PacBio sequencing has a not insignificant base error rate, which is higher than current Illumina sequencing. If matched Illumina cDNA sequencing data is available, a direct comparison to estimate the sequencing error rate and its impact on the mass spectral matching should be done: in other words, does the current method underestimate alternative isoforms. This may be inferred from the analysis of mass spectral sequence tags or spectra identified de novo to determine the influence of transcript sequencing error rate.

3) ORF prediction is a key element of the method. The choice of CPAT seems reasonable, but a direct comparison with TransDecoder or another suitable algorithm is warranted.

4) The manuscript compares the results of mass spectral searches against PacBio-derived database and a database containing PacBio-identified transcripts and GENCODE transcripts. How many proteins and isoforms are identified by searching PacBio-derived database exclusively? How does this relate to question #1.

5) For the novel transcript isoforms detected using the method, how many have mass spectral evidence? Of the isoforms that lack mass spectral evidence, how many do so because of the absence of theoretically suitable peptide sequences (lack of enzyme digestion site, lack of charge, etc) versus the number that are theoretically expected, but not detected?

6) Is there either transcript evidence with or without mass spectral support for genes that are not currently annotated?

7) How many transcripts have features, such as exon-exon junctions or chimeric sequences, emanating from somatic DNA mutations, such as translocations, deletions etc, that are common in Jurkat cells?

**Authors Response**

**Point-by-point responses to the reviewers’ comments:**

*We thank all the reviewers for their valuable and constructive feedback. Please see our response to the comments below.*

*(Note that all page numbers being referred to are based on the newly revised manuscript that includes tracked changes.)*

**Reviewer #1 Comments:**

The detection of translated protein isoform using mass spectrometry remains challenging. Here the authors explored the feasibility of using long-read PacBio sequencing to improve protein isoform inference in shotgun proteomics. A major result here is that full length isoform transcripts in Jurkat cells can significantly differ from GENCODE reference transcripts, which will have important implications for isoform inference and proteogenomics studies. A protein inference scheme and software pipeline is also provided for use. Overall this looks like a promising study that will be of interest to other researchers employing proteogenomics methods, but I have some comments regarding the implementation and benchmarking in their current form.

***Response:*** *We thank the reviewer for the positive comments.*

*Major comments:*

1. Although the title and central message of the paper suggest that long read sequencing enhances isoform identification, the results appear to only showcase that it is *feasible* to use long read sequencing to identify isoforms. Was there any attempt to compare with common existing workflows using short read sequencing to show practical improvement in isoform detection? This could be done for instance using public Jurkat cell Illumina sequencing data and current in silico translation software packages like CustomProDB and others.

***Response:***

*The reviewer poses a very good question, which we considered over the course of the project. We found that due to the differences in outputs of short-read RNA-seq versus long-read RNA-seq, many different types of comparisons can be done. From short-read RNA-seq data, several types of databases can be constructed, such as a variant database (most commonly used in the field) containing single amino acid variants. However, we determined that the most relevant short-read-based proteogenomics pipelines to compare our workflow against would be one that constructed a splice-related database.*

*In consideration of the reviewer's response, we investigated CustomProDB which uses aligned but not assembled short read RNA-sequencing reads. We ran CustomProDB (Bioconductor version: Release (3.14)) and prepared our annotations by running PrepareAnnotationEnsembl. Specifically, we used the command:*

*PrepareAnnotationEnsembl(mart=ensembl, annotation_path=annotation_path, splice_matrix=TRUE, dbsnp=NULL, transcript_ids=transcript_ids, COSMIC=FALSE).*

*This annotation preparation provided us with, amongst other things, six files for use in all subsequent analyses:*

1. *exon_anno.RData*
2. *ids.RData*
3. *procodingseq.RData*
4. *proteinseq.Rdata*
5. *splicemax.RData*
6. *txdb.sqlite*

*CustomProDB enables the creation of three different types of protein databases: 1) A reduced database in which includes a subset of reference isoforms which have evidence for expression, 2) A variant database that includes Single Amino Acid Variant (SAAV) entries, and, most relevantly, 3) A database of pairs of exons (i.e., junctions).*

*The third database, the splice-junction database, is the only database product of CustomProDB which utilizes short-read sequencing information in an effort to enable the identification of novel protein isoforms. This database is constructed from the identification of previously unannotated structures such as novel exons, alternative splicing and gene fusions. A BED file is used that contains the splice junctions from the RNA-seq data. A function JunctionType classifies all the junctions into six categories.*

1. *Connect two known exon (sub-label: known junction, alternative splicing, gene fusion)*
2. *Connect one known exon and one region overlap with known exon*
3. *Connect one known exon and one non-exon region*
4. *Connect two regions both overlapped with known exons*
5. *Connect one known exon and one non-exon region*
6. *Connect two regions both overlapped with known exons*
7. *Connect one region overlap with known exon and one non-exon region*
8. *Connect two non-exon regions.*

*All putative novel junctions are three-frame translated into peptides with the function OutputNovelJun. This is done with the reference genome. This is likely the most relevant database to compare with the databases we construct.*

*That being said, the resulting database produced by the OutputNovelJun is a peptide fragment database, whereas with our workflow, we uncover and report not just novel junctions, but novel full length transcript isoforms, 5’ to 3’.*

*To verify this, we ran the junction on available test data and output the junction file, which shows short polypeptides associated with alternatively spliced sites. A script documenting the analysis steps is included in the Revision Submissions (entitled ` CustomProDB_Analysis_Script_Reviewer#1_Response.txt `). An example output is provided below:*

*>JUNC00101237_chr9:86584295-86585077|14|ORF1 |Junpos:24-25|-|NA|NA|connect two non-exon region*

*RRSDHYHYRNTGPDTECTVFAAEQCEAVFWKVFLRLVKN*

*>JUNC00041587_chr17:7579940-7590695|29|ORF1 |Junpos:23-23|-|NM_001126112|NM_001126112|connect a known exon and a region overlap with known exon*

*PGTLCVRAGSVLSTTVTRFPGLAARLPSGSLPWRSRSQILASSP*

*Comparing this output to the output from our Long Read Proteogenomics workflow based upon translation of the full length RNA sequences*

*>pb|PB.5982.3|fullname GN=FYN*

*MGCVQCKDKEATKLTEERDGSLNQSSGYRYGTDPTPQHYPSFGVTSIPNYNNFHAAGGQG*

*LTVFGGVNSSSHTGTLRTRGGTGVTLFVALYDYEARTEDDLSFHKGEKFQILNSSEGDWW*

*EARSLTTGETGYIPSNYVAPVDSIQAEEWYFGKLGRKDAERQLLSFGNPRGTFLIRESET*

*TKGAYSLSIRDWDDMKGDHVKHYKIRKLDNGGYYITTRAQFETLQQLVQHYSEKADGLCF*

*NLTVIASSCTPQTSGLAKDAWEVARRSLCLEKKLGQGCFAEVWLGTWNGNTKVAIKTLKP*

*GTMSPESFLEEAQIMKKLKHDKLVQLYAVVSEEPIYIVTEYMNKGSLLDFLKDGEGRALK*

*LPNLVDMAAQVAAGMAYIERMNYIHRDLRSANILVGNGLICKIADFGLARLIEDNEYTAR*

*QGAKFPIKWTAPEAALYGRFTIKSDVWSFGILLTELVTKGRVPYPGMNNREVLEQVERGY*

*RMPCPQDCPISLHELMIHCWKKDPEERPTFEYLQSFLEDYFTATEPQYQPGENL*

*>pb|PB.5982.8|fullname GN=FYN*

*MGCVQCKDKEATKLTEERDGSLNQSSGYRYGTDPTPQHYPSFGVTSIPNYNNFHAAGGQG*

*LTVFGGVNSSSHTGTLRTRGGTGVTLFVALYDYEARTEDDLSFHKGEKFQILNSSEGDWW*

*EARSLTTGETGYIPSNYVAPVDSIQAEEWYFGKLGRKDAERQLLSFGNPRGTFLIRESET*

*TKGAYSLSIRDWDDMKGDHVKHYKIRKLDNGGYYITTRAQFETLQQLVQHYSERAAGLCC*

*RLVVPCHKGMPRLTDLSVKTKDVWEIPRESLQLIKRLGNGQFGEVWMGTWNGNTKVAIKT*

*LKPGTMSPESFLEEAQIMKKLKHDKLVQLYAVVSEEPIYIVTEYMNKGSLLDFLKDGEGR*

*ALKLPNLVDMAAQVAAGMAYIERMNYIHRDLRSANILVGNGLICKIADFGLARLIEDNEY*

*TARQGAKFPIKWTAPEAALYGRFTIKSDVWSFGILLTELVTKGRVPYPGMNNREVLEQVE*

*RGYRMPCPQDCPISLHELMIHCWKKDPEERPTFEYLQSFLEDYFTATEPQYQPGENL*

*>gc|LIME1-202| GN=LIME1*

*MGLPVSWAPPALWVLGCCALLLSLWALCTACRRPEDAVAPRKRARRQRARLQGSATAAEA*

*SLLRRTHLCSLSKSDTRLHELHRGPRSSRALRPASMDLLRPHWLEV*

*>gc|LIME1-206| GN=LIME1*

*MGLPVSWAPPALWVLGCCALLLSLWALCTACRRPEDAVAPRKRARRQRARLQGSATAAEA*

*VSARLSRGPGWGPQGTDQPSSPPVPTEADPPLLPQQVGHQTARAAPGPAQQQGPAACQHG*

*SPAPTLAGGVQGHHRTAGSPLCLPTPGAAPGSAGSCSHRRVRWPRGHLFQRGAGGPSRGQ*

*PGGQPCGGRVCPRPEAQRDPSQSPRATAGED*

*CustomProDB succeeds in including variation information and novel splice junction information but does not assemble these features into full length transcript isoforms prior to translation. We seriously considered and discussed in-depth the possible ways of comparison but could not determine an adequate approach for comparing isoform identification abilities. We concluded that due to a lack of an external gold standard, any comparison would be challenging and potentially biased, since only with long-read RNA-seq one can obtain the full connection between all exons, which is needed to unambiguously predict the full-length protein isoform. Current efforts through consortia such as the Long-read RNA-seq Genome Annotation Assessment Project (LRGASP) include ongoing studies for a more extensive comparison between short-read and long-read results, including an approach for validation and assessment of sensitivity and precision, and we look forward to such work to inform the next generation of our pipeline.*

2. Based on some of the examples given, e.g., LNPK-201 vs. LNPK-212; and MECP2-205 vs. MCEP-201 (pg.12 line 25) it is hard to see immediately what benefits do the proteogenomic and ORF finding workflow bring. It appears that the PacBio data mainly show that among two LNPK GENCODE transcripts (LNPK-201 vs. LNPK-212), the 201 isoform is more likely to be truly expressed; and that likewise among MECP2-205 and MECP2-201, 205 is expressed but not 201.

***Response:*** *We thank the reviewer for this comment. The examples of LNPK and MECP2 were selected to represent two different scenarios where, because of differing isoform composition of the PacBio-Hybrid and GENCODE reference databases, the resulting protein groups inferred were composed of different protein isoforms. Just as isoform models can be compared across databases, the isoform composition of the inferred protein groups can also be compared and categorized into the same groups: a) match, b) subset, c) superset, d) partial overlap and e) distinct.*

*Protein group isoform composition is inferred based on the peptides identified. The first part of the protein inference algorithm is to map an identified peptide back to all possible protein isoforms that the peptide could have possibly originated from, based on the database searched. This means that the isoform composition of the protein database can have a profound effect on the protein inference results, especially for the many situations where no isoform-specific peptides were identified. For most protein groups compared across the PacBio-Hybrid and GENCODE protein inference results, the peptide identifications were identical. Therefore, the differences in inferred protein isoforms can be directly tied back to the different isoform models within the protein databases.*

*LNPK represents a subset scenario, where based on the PacBio sequencing information, we know that only one reference isoform is expressed in the sample, LNPK-201. Therefore, the proteogenomic database only consists of that single isoform entry for the gene LNPK. This contrasts to the reference where there are six different isoforms present for the gene LNPK. On an isoform model level, the PacBio database represents a subset of the isoform present in the reference. This relationship has an effect on the results of the downstream proteomic analysis as well. Although the peptide identifications for proteins from the LNPK gene are identical regardless of whether the PacBio-Hybrid or GENCODE reference database are searched, the protein isoforms inferred differ based on the database composition. When using the GENCODE reference database, an ambiguous protein group composed of LNPK-201 and LNPK-212 is generated because, based on the peptide evidence and the isoform model present in the database, we are unable to differentiate between them. However, when the PacBio-Hybrid database is searched, only one protein isoform is present in the database, and therefore inferred, LNPK-201. Therefore, the protein group inferred with the PacBio-Hybrid database also represents a subset of the protein group inferred from the GENCODE reference database. We were able to reduce the ambiguity of the protein inference results, because our PacBio-Hybrid database, within the high confidence space, is only composed of protein isoforms backed by transcriptional support.*

*MECP2 represents a partial overlap scenario, where based on PacBio sequencing information, we know that there is only one reference isoform expressed in the sample, but the possibility of multiple novel isoforms. The isoform models of the PacBio-Hybrid database and GENCODE reference database partially overlap sharing only MECP2-205. The PacBio-Hybrid database’s isoform model for the gene MCEP2 has an additional 3 novel protein isoforms whose transcripts were identified by PacBio sequencing. The GENCODE reference database’s isoform model has 9 other reference isoforms. Again, all peptides were identified in both the PacBio-Hybrid and GENCODE reference database searches. For the GENCODE reference database search, these peptides could have originated from either MECP2-205 or MECP2-201, creating an ambiguous protein group. In the PacBio-Hybrid sequencing results, there was no transcriptional evidence for the protein isoform MECP2-201. For the PacBio-Hybrid database, MECP2-205 or one of the novel protein isoforms, PB.16836.37, could explain the peptides identified. Therefore, an ambiguous protein group composed of MECP2-205 and PB.16836.37 were identified. The protein groups when compared against each other represent a partial overlap scenario because one inferred protein isoform is shared, MECP2-205, and each protein group also has another, not shared protein isoform.*

*These are just two examples of the many differences in inferred protein groups that are a direct result of the differences of the isoform models in the PacBio-Hybrid and GENCODE reference databases. We suppose that the PacBio-Hybrid database enables the inference of protein groups that are more reflective of the sample’s proteome because the isoforms are inferred by both proteomic and transcriptomic data. Of course, comprehensive validation of such results is an ongoing challenge to our lab and the field, and we will continue to work towards addressing validation approaches in follow-up studies. We hope that our open-source pipeline and freely accessible datasets and initial workflow will be of value to the field.*

*In an effort to make the impact of these examples more clear in the Results section (page 12-13):*

*“Based purely on MS peptide evidence, there is ambiguity in terms of whether the isoform LNPK-201 or LNPK-212 is expressed, but the PacBio transcript evidence indicates LNPK-201 is the main isoform likely to be expressed in the cell line.”*

*“Using the GENCODE database as reference, MECP2-205 and MECP2-201 form a single protein isoform group and are indistinguishable based on the peptide evidence. However, when using the PacBio-Hybrid database, there was no transcriptional support for MECP2-201 is not expressed and. Instead, MECP2-205 forms a protein isoform group with the novel PacBio-derived isoform PB.16836.37.”*

**a.** What would be the outcome if the authors simply searched the MS data with the GENCODE database then annotated the result based on PacBio read count data?

***Response:*** *If the GENCODE database was searched, and the PacBio sequencing data was only used to provide transcriptional abundance information, we suppose that the search results obtained on the protein level would be very different and in some cases may be less accurate or not as reflective of the sample’s true proteomic composition. There are only 4,503 protein groups that are identical between the protein inference results of the GENCODE database search and the PacBio-Hybrid database search. For the other approximately 3,200 protein groups, different answers are obtained due to discordances in the protein isoform database compositions. For some of these cases, where a reference database isoform is absent in the PacBio-Hybrid database due to lack of long-read transcriptional support, the results could be recapitulated. However, there are many isoforms that are present only in the PacBio-Hybrid database, and protein groups containing these isoforms would not be able to be recreated using the reviewers suggested strategy. This would account for 839 protein groups that are categorized as partially overlapping with the GENCODE protein inference results and 329 of the protein groups that are unique to the PacBio-Hybrid database’s protein inference results. In total these protein groups account for approximately 15% of the protein groups identified at 1% protein FDR. This is not an insignificant value, and demonstrates the importance of using long-read sequencing to generate a sample-specific database, instead of solely informing the search of a reference protein database.*

b. Secondly, did the authors investigate whether short-read sequencing data followed by transcript assembly would have been able to tell apart the difference between LNPK-201 and LNPK-212?

*Response: We appreciate this comment. To investigate the ability of short-read sequencing data to come to the same conclusion regarding the delineation of reference isoform expression that was obtained from long-read sequencing, we analyzed Jurkat RNA-seq data (GSE45428), with Kallisto (PMID: 27043002) using as a reference the GENCODE transcriptome (version 35). For the case of LNPK-201 and LNPK-212, the determined transcriptional abundance for these isoforms via Kallisto was 8.88 TPM and 1.45 TPM, respectively. Based on this, if the short-read sequencing, via alignment, was used to construct a sample specific database, both isoforms would be present. For both short and long-read sequencing, the same isoform was deemed to be the most abundant.*

*This question piqued our curiosity, and we expanded our analysis to all of 673 protein groups that were composed of a subset of the isoforms in the PacBio-Hybrid protein inference results than were present in the GENCODE protein inference results. We wanted to investigate what proportion of these differences would also be recapitulated using short-read sequencing. For this analysis, we decided to select the single most abundant isoform for each group. For 321 protein groups, or 47%, selecting the most abundant isoform did not provide the same answer as what is achieved using long-read sequencing. This may be because the use of the reference database (GENCODE) does not fully reflect the isoforms expressed in the sample. It may also indicate platform (short- or long-read based) or technical biases.*

3. The authors provide a new inference algorithm "Rescue & Resolve" here, but the exact contribution of the algorithm to improving protein isoform identification is not clearly described in the manuscript. One would assume that the use of long read sequencing data and full-length transcript assembly by itself already resolved many of the most challenging inference problems since fewer isoform transcripts are present in the database. Does the algorithm also work with the more common short read assembled databases?

***Response:*** *This comment is valuable as it has pointed out places in which we could be more explicit in stating the improvements in protein isoform identification based on the implementation of the “Rescue & Resolve” algorithm. We have added and revised text in the manuscript to address this valuable feedback.*

*We added text to the Results (page 18):*

*“Collectively, the “rescued” protein isoforms represented a 6.5% increase in the number of PacBio-derived protein isoforms identified at 1% FDR, compared to what is obtained without the “R&R” algorithm, using MetaMorpheus’ traditional parsimonious approach.”*

*We added text to the Results (page 19):*

*“We found that in 1,434 cases, one isoform comprises more than 90% of the transcript abundance, suggesting that a single dominant isoform could comprise the group. For these dominant isoform-containing protein groups, the ambiguity of which protein isoform is present within the sample was resolved, and a single protein isoform was considered to be identified, increasing the precision of the protein inference results obtained. Notably, not all protein groups can, or should be resolved to a single isoform. There are cases where multiple protein isoforms are co-expressed and the peptide evidence is not comprehensive enough to be able to sufficiently distinguish them. It is important to maintain protein group ambiguity when necessary and valid.”*

*The reviewer also asks an important question regarding the utility of the algorithm with short-read assembled databases. The Rescue & Resolve algorithm would work with a short-read derived sample specific database and accompanying transcriptional abundance estimates. However, there is the potential for increased error with the use of short-read RNA-seq data. The process of estimating transcriptional abundance from RNA fragment abundances requires an inference-like process of its own, and there is error associated with it that would be carried through to the estimate of the isoforms transcriptional abundance. Accumulation of this specific source of error is avoided when long read sequencing is utilized because the measurements come directly from the observation of full, intact transcripts. To address the reviewer’s question we have added text to the discussion section of the manuscript, stating that the algorithm could be applied to short-read assembled databases.*

*We added text to the Discussion (page 22):*

*“Although the Rescue & Resolve algorithm was developed for use with long-read sequencing information, the algorithm could also be applied to proteogenomic databases and transcriptional abundance information derived from short-read sequencing approaches.”*

4. Related to the above, the manuscript should better clarify what the Rescue & Resolve algorithm does. As best as I could tell, it seems be that whenever there is a peptide identified to a protein group containing two transcripts and if the long read abundance of one of those two transcripts is responsible for over 90% of the transcript reads then the algorithm assumes the higher abundance transcript is uniquely found and disregards the lesser isoform.

***Response:*** *We appreciate this comment and insight from the reviewer regarding the clarity of our description of the Rescue & Resolve algorithm.This feedback has prompted a major re-write of the results section describing the Rescue & Resolve algorithm in an attempt to better clarify the logic and process of this heuristically-driven algorithm. In the comment above, the reviewer describes the “resolve” portion of the algorithm but not the “rescue” portion.*

*Changes in the manuscript can be observed on pages 16 to 19. The main blocks of added and revised text can be found below:*

*“As previously mentioned, the parsimonious protein inference process makes decisions throughout the algorithm to discard, or eliminate, protein isoforms from consideration for identification, because they lack the same level of peptide evidence that competing isoforms possess. During this process, protein isoforms that are actually present in the sample could be eliminated, generating false negatives. The “rescue” portion of our “R&R” algorithm defines two cases in which a protein isoform could be “rescued” from elimination (Fig. 6a). The first case occurs when a protein isoform’s mapped peptides are a subset of the peptides mapped to another protein isoform (Case 1, Fig. 6a). In this scenario, the parsimonious algorithm would determine that the protein isoform which accounts for the most peptides is the simplest answer, and therefore more likely to be correct by the principle of Occam’s razor. The protein isoform that accounts only for a subset of the peptides observed is eliminated from consideration for identification. The second case occurs when a protein isoform’s mapped peptides are subsumable to (i.e., can be explained by) two or more protein isoforms which have additional peptide evidence (Case 2, Fig. 6a). In this scenario, there is a protein isoform for which all of its peptide evidence can be explained by the existence of multiple protein isoforms that all have more peptide identifications supporting their existence. Again, as in Case 1, the parsimonious approach dictates that it is simpler, and therefore more likely, that the protein isoforms with additional peptide support are the sole contributors to the peptides being identified. The subsumable protein isoform is then eliminated from consideration for identification. In the “rescue” portion of our R&R algorithm, during the parsimonious process, protein isoforms that were eliminated due to scenarios such as Case 1 and Case 2, are identified, and set aside as potential false negatives that can be “rescued” from elimination. To determine whether or not a protein isoform should be “rescued” or eliminated, the long-read transcriptional abundance information obtained for each isoform is leveraged as an additional source of data. Since RNA abundance is at least moderately correlated with protein expression[25,52] (R-squared=0.65, Supplementary Fig. 5a), a high abundance transcript would have a higher probability, than a low abundance transcript, of generating the corresponding protein which was observed in our dataset (Supplementary Fig, 5b). In the R&R algorithm, protein isoforms are only rescued from elimination if their transcriptional abundance is greater than a user-specified abundance threshold. We selected a conservative transcript abundance threshold of 25 CPM (see Supplemental Note 45 for parameter optimization details). The impact of the “rescue” portion of the “Rescue & Resolve” algorithm on the protein inference results obtained were compared to those obtained with the traditional parsimonious protein inference algorithm within MetaMorpheus (details regarding MetaMorpheus’s inference algorithm can be found at https://github.com/smith-chem-wisc/MetaMorpheus/wiki/Protein-Parsimony-&-Grouping-(Protein-Inference)).”*

*“The “resolve” portion of the R&R algorithm addresses a third scenario which can arise during protein inference, (Case 3, Fig. 6a), where the parsimonious process generates ambiguity through a protein group which contains two or more indistinguishable protein isoforms (based on equivalent peptide evidence). Ambiguous protein groups can be composed of three different classes of isoforms categorized by their relative transcriptional abundance: 1) dominant (a “resolved” isoform), 2) minor or 3) co-expressed. The “resolve” portion of the algorithm provides the opportunity to “resolve” these ambiguous protein groups to a single, dominant isoform, or provide support for the co-expression of multiple protein isoforms based on relative transcriptional abundance of each isoform within the group. For instances of Case 3, the relative transcriptional abundances underlying the predicted protein isoforms could indicate likelihood of expression.”*

a. What current protein inference algorithm is R&R being compared against?

***Response:*** *R&R algorithm is being compared against the traditional parsimonious protein inference algorithm inside of MetaMorpheus that is used by default.*

*We have added text in the Results to explicitly state this (page 17):*

*“The impact of the “rescue” portion of the “Rescue & Resolve” algorithm on the protein inference results obtained were compared to those obtained with the traditional parsimonious protein inference algorithm within MetaMorpheus (details regarding MetaMorpheus’s inference algorithm can be found at https://github.com/smith-chem-wisc/MetaMorpheus/wiki/Protein-Parsimony-&-Grouping-(Protein-Inference)).”*

*Additionally, we have revised text, underlined, in the Results (page 18):*

*“Collectively, the “rescued” protein isoforms represented a 6.5% increase in the number of PacBio-derived protein isoforms identified at 1% FDR, compared to what is obtained without the “R&R” algorithm, using MetaMorpheus’ traditional parsimonious approach”*

b. Logically it would appear to me that one cannot assume that the isoform peptides come from only the higher abundance isoform. For instance, 10% of the peptides might feasibly come from the lower protein isoforms with 10% read abundance. Conversely even if two isoforms are found in the PacBio data with >30% relative abundance, they could have different translation potential and only one could make a stable protein product so without empirical evidence of a unique peptide one couldn't just assume both are translated. Isn't this the reason to perform proteo(geno)mics experiments to begin with, since one cannot assume the transcript is translated?

***Response:*** *This is an excellent point. There will be cases in which the low abundance isoform will indeed be expressed, and also cases where co-expression on the protein level does not happen regardless of transcriptional evidence for co-expression. However, proteomic sensitivity and coverage is not complete enough to definitively state that if isoform-informative peptides are not observed then the protein product is not present. The strength of proteogenomics lies in the fact that you can integrate information from the transcriptome and proteome together to generate a more comprehensive view of the proteome than what can be obtained with either alone. We show here in our study, Supplementary Figure 5b, that the greater the transcriptional abundance of an isoform, the more likely it is to be translated into protein and observed via proteomics. This may not always hold true as there are exceptions in translational efficiency, protein degradation and RNA degradation, but the assumption can be applied on a proteome-wide scale, as is done here in this work.*

*Due to the points above, we do not “resolve” protein groups automatically, but provide the user with fractional isoform abundance information so they are empowered to select the abundance cut-off that they are comfortable with. To make this point clear, we added clarification in the Results, underlined text (page 19):*

*“Our algorithm provides the relative transcript abundance measures for protein isoforms within a group, enabling the opportunity to resolve isoform identifications based on underlying transcript support, which is fully at the discretion of the user (Supplementary Fig. 5c).”*

*To call attention to the point of differential translation efficiencies between isoforms of the same gene, we added text in the Discussion to make clear this limitation (page 23):*

*“First, for the “Rescue and Resolve” approach, we assume at least a moderate degree of RNA-protein correlation. Although isoforms from the same gene should not greatly differ in their transcript-protein correlation, several studies have reported isoform-specific mRNA translation[63,64] suggesting that alternative splicing can generate transcripts with distinct cis-regulatory landscapes. Therefore, caution must be taken for any given protein isoform, including follow-up confirmation of expression in vivo.”*

*References 63 and 64 correspond to:*

*Sterne-Weiler T, Martinez-Nunez RT, Howard JM, Cvitovik I, Katzman S, Tariq MA, Pourmand N, Sanford JR. Frac-seq reveals isoform-specific recruitment to polyribosomes. Genome Res. 2013 Oct;23(10):1615-23. doi: 10.1101/gr.148585.112. Epub 2013 Jun 19. PMID: 23783272; PMCID: PMC3787259.*

*Floor SN, Doudna JA. Tunable protein synthesis by transcript isoforms in human cells. Elife. 2016 Jan 6;5:e10921. doi: 10.7554/eLife.10921. PMID: 26735365; PMCID: PMC4764583*.

**c.** Existing software like MaxQuant already report total protein groups identified, so the present method would seem to not so much as improve protein IDs than to provide a way to annotate redundant protein groups. In this case one might argue whether the algorithm is needed beyond simply applying a read count criterion when creating the protein database.

***Response:*** *We thank the reviewer for their comment. However, we respectfully disagree on the point that the inclusion of transcriptomic information would only serve to “annotate redundant protein groups”. MetaMorpheus, like MaxQuant, provides protein group identifications on its own. The addition of the Rescue & Resolve algorithm increases the number of protein groups identified via the “Rescue” portion of the algorithm (see page 18) and decreases the ambiguity of protein group assignments through the “Resolve” portion of the algorithm. This heuristic-based algorithm does not just annotate redundant protein groups but actually affects the number of protein groups identified at 1% protein FDR. By increasing the number of protein groups, and reducing overall ambiguity of protein inference results, the “Rescue & Resolve” algorithm improves the protein identifications obtained.*

*To demonstrate the impact of CPM filtered databases on the proteomic results obtained, 6 filtered databases were constructed containing PacBio-derived isoforms greater than or equal to the CPM threshold applied (1 CPM, 2 CPM, 3 CPM, 4 CPM, 5 CPM and 10 CPM). These databases were then searched using MetaMorpheus and the results compared to the search results of the original PacBio-Hybrid database. The results of this analysis are displayed below in three plots. Restricting the database by CPM results in a reduction of peptide identifications at each threshold. This is because there are peptides in the sample whose sequences are only represented in protein isoforms that were eliminated due to the applied read count criterion. Losing peptide identifications causes downstream effects on the protein level results of the analysis. Peptides serve as input for protein inference, and having only a subset of the peptides identified with the full database results in fewer protein group identifications. Not only are there fewer protein groups, but the identity (or isoforms that compose the identified protein groups) also are different because of fewer overall peptide identifications, and the loss of protein isoform options for which shared peptides could originate. Filtering the protein database by read count does not produce the same results that are obtained when the R&R algorithm is utilized.*

**d**. If the lesser transcript is at all found within the long-read sequencing data, it would seem to me that true protein isoform inference would require isoform assembly at the protein level through probabilistic modeling. I would suggest more clearly describing R&R as a heuristic filter as the authors mentioned in page 20, rather than an inference algorithm throughout the manuscript.

***Response:*** *We thank the reviewer for their thoughts. We agree that we should be clear about the heuristic nature of R&R. However, we believe that, though simple, the Rescue & Resolve approach can still be classified as an algorithm. An algorithm is defined, by Oxford Languages, as “a process or set of rules to be followed in calculations or other problem-solving operations, especially by a computer”. By this definition, we believe the “Rescue & Resolve” approach is an algorithm. There are heuristic components to the algorithm, which is not uncommon in parsimonious protein inference algorithms where heuristics play a stronger role. The “resolve” portion of the algorithm can be viewed more as a heuristic filter, but the rescue component of the algorithm involves more than just filtering, as determinations of why an isoform was discarded from the parsimonious process is made, and whether they are candidates for rescuing. To address the reviewer’s comment, we have added throughout the manuscript the distinction that the “Rescue & Resolve” approach is a heuristic-based algorithm. These additions can be found on pages 5, 16 and 22.*

*In the discussion section, we discuss future plans to create a probabilistic protein inference algorithm incorporating long-read abundance information. Based on the reviewer’s comment, we have revised this text to further emphasize this future direction.*

*We have revised the text, underlined, in the Discussion (page 22):*

*“Another improvement to the pipeline will be an evolution of the heuristically-driven “Rescue & Resolve'' approach. We plan to develop a probabilistic protein inference algorithm in which transcriptional abundance values are incorporated into a rigorous statistical framework for the inference of protein isoforms[43,60].”*

**e.** Please also clarify what the permutation test on page 17 is testing for. What is being permuted, e.g., are the labels being swapped randomly for the relative read proportion within a protein group, or do the authors redraw from the distribution of the entire population of proteins/isoforms etc.

***Response:*** *We thank the reviewer for their feedback. The original manuscript was not clear on these points. A more detailed description of the permutation analysis can be found in what was previously Supplemental Note 5, but is now Supplemental Note 6. Based on the reviewer’s comment, we further expanded on the description of the permutation analysis.*

*We revised the text in the supplemental note (pages 13-16):*

*“For validation of “rescued” and “resolved” protein isoforms, an independently generated multi-protease dataset was used. Spectra from the analysis of six fractionated proteolytic digests (see Methods) were searched against the PacBio-Hybrid database using MetaMorpheus, and the multi-protease protein inference algorithm was employed[16]. The multi-protease protein inference results are considered to be more comprehensive, and reflective of the sample’s proteome compared to what can be achieved with trypsin alone. The use of orthogonal proteases provides higher, and more distinctive coverage of protein isoforms. One product of this is the identification of more unique peptides, which can confidently identify protein isoforms. Although the multi-protease protein inference results are not a perfect model of the isoforms expressed in the sample, for the purpose of our validation strategy, we will consider the results as a “ground truth” dataset.*

*For the purposes of validation, we determined if the protein isoforms that were “rescued” or “resolved” by “Rescue & Resolve” algorithm were isoforms that were identified in the multi-protease protein inference analysis. If a “rescued” or “resolved” protein isoform was identified—in the multi-protease analysis—as a single protein isoform, not as part of a multi-isoform protein group, the identification of the protein isoform in question was considered to be confirmed, or “validated”. These validated “rescued” or “resolved” isoforms, if identified in the multi-protease protein inference results, had sufficient peptide level evidence, such as an isoform-specific peptide, due to the identification of additional non-tryptic peptides derived from orthogonal proteases to support their confident identification. The percent of “rescued” and “resolved” protein isoforms whose presence were confirmed, or “validated”, in the multi-protease protein inference results can be calculated. This percent validation rate, when compared to expected rates, indicates how well the “rescue” and “resolve” portions of the “R&R” algorithm do at increasing the number of true positive protein isoform identifications.*

*Since the multi-protease protein inference results are still incomplete and subject to error due to incomplete peptide coverage of the proteome, the percent validated means very little on its own. To assess the significance of the percent of “rescued” or “resolved” isoforms validated, the experimentally determined value can be compared against the validation rates expected at random. Such values can be computed by calculating the percent of protein isoforms validated for a pool of randomly “rescued” or “resolved" protein isoforms.*

*For the evaluation of the “rescue” portion of the “Rescue & Resolve” algorithm we compared the rate of validation between the “rescued” isoforms and randomly selected isoforms (background null). We “rescued” 355 isoforms based on additional transcriptional evidence, from a pool of 15,700 isoforms that represent all the protein isoforms that could possibly be “rescued”. The same number of protein isoforms that were rescued in the experimental results (N=355), were randomly selected, agnostic of transcriptional abundance, from the pool of 15,700 protein isoforms that were discarded in the protein inference process. Once the randomly “rescued” isoforms have been selected, we determined if such protein isoforms were identified in the multi-protease protein inference results, and the percent of isoforms validated was calculated just as was done for the experimental results. This process of randomly selecting 355 protein isoforms to “rescue” and determining the validation rate was repeated for a total of 1,000 permutations to generate a null distribution of validation rates against which the experimentally obtained results were compared, and statistical significance with a p-value <0.0001 was determined.*

*For the evaluation of the “resolve” portion of the “Rescue & Resolve” algorithm, a random protein isoform was selected as the dominant isoform, or “resolved” isoform, for each of the 1,434 protein groups that were resolved to a single, dominant, protein isoform in the experimental results. The randomly “resolved” isoforms were compared to the protein isoforms identified in the multi-protease protein inference results and the percent of isoforms validated was determined. This process of randomly selecting a single isoform for each resolvable protein group was repeated for a total of 1,000 permutations to generate a null distribution of validation rates against which the experimentally obtained results were compared and the statistical significance with a p-value <0.0001 was determined. For this permutation analysis, the chance of randomly selecting the dominant protein isoform that was resolved experimentally was much higher due to a smaller pool of candidates for random selection, which included a mixture of isoforms that were “resolved” (deemed expressed) mixed in with isoforms which were discarded (deemed not expressed, based on transcriptional evidence). Because of this an additional experiment was conducted, in which all the minor protein isoforms that were removed during the resolution of protein groups were compared against the obtained multi-protease protein inference results. This provides a percent validation rate for all of the isoforms removed during the experimental resolution of protein groups. This value was also compared to the null distribution for statistical significance with a p-value <0.0001.”*

*Within the main result section we have added additional references to Supplemental Note 6 on page 18 and 19.*

**f.** The performance of the inference is hard to verify currently and this should be made more clear. The multi-protease dataset doesn't necessarily provide a gold standard as more abundant transcripts are more likely to be found in the multi-protease mass spec data due to their detectability. The more important question here is whether the "resolved" transcript that gets removed is truly absent and how might one validate that and establish ground truth. Is there a difference in inference performance based on total transcript abundance since presumably for those genes even the transcript with lower read proportion would be more likely detectable?

***Response:*** *This is an excellent point, and we agree that validation of inference results is exceedingly challenging, for us and the field. What used to be Supplementary Note 5 , and is now Supplementary Note 6, has text which states the difficulty of validating protein inference results. The text of interest can be found on page 13 of the Supplemental Notes and has been pasted below for convenience.*

*“We hypothesize the R&R algorithm enables a more precise representation of sample isoform diversity than can be achieved using a traditional protein inference approach. However, the experimental validation of protein isoforms inferred from MS data is an ongoing challenge[8,9]. Existing analytical standards fail to appropriately model the complexity of endogenous isoforms[10,11], and the lack of knowledge regarding protein isoforms present in a sample (i.e., ground truth) is a problem in and of itself[12–14]. In place of experimental validation, studies have established heuristic guidelines to compare protein inference results between different inference algorithms[8,15]. Here we have developed a computational strategy that leverages the improved accuracy and precision of multi-protease protein inference to validate both the “rescued” and “resolved” protein isoforms.”*

*We added text to the Results (page 18):*

*“Validation of protein inference approaches is exceedingly difficult, in that we do not know the true composition of the sample, and standard protein mixtures lack the complexity necessary to model the human proteome. This is especially true in the case of modeling human isoform diversity where the “Rescue & Resolve” algorithm is most beneficial.”*

*The consideration of the multi-protease data as “ground truth” is also further elaborated in Supplementary Note 6. Although this is not a perfect validation system, we believe that it is a valid method for computationally validating the “Rescue & Resolve” algorithm.*

*We have revised and added text in Supplementary Note 6 (page 13-14):*

*“The multi-protease protein inference results are considered to be more comprehensive, and reflective of the sample’s proteome compared to what can be achieved with trypsin alone. The use of orthogonal proteases provides higher, and more distinctive coverage of protein isoforms. One product of this is the identification of more unique peptides, which can confidently identify protein isoforms. Although the multi-protease protein inference results are not a perfect model of the isoforms expressed in the sample, for the purpose of our validation strategy, we will consider the results as a “ground truth” dataset.”*

*We also note that the current description of how the multi-protease protein inference results are used to confirm the results of the “Rescue & Resolve” algorithm in Supplemental Note 6 may be unclear.*

*We revised the text in Supplemental Note 6 (page 14):*

*“For the purposes of validation, we determined if the protein isoforms that were “rescued” or “resolved” by “Rescue & Resolve” algorithm were isoforms that were identified in the multi-protease protein inference analysis. If a “rescued” or “resolved” protein isoform was identified—in the multi-protease analysis—as a single protein isoform, not as part of a multi-isoform protein group, the identification of the protein isoform in question was considered to be confirmed, or “validated”. These validated “rescued” or “resolved” isoforms, if identified in the multi-protease protein inference results, had sufficient peptide level evidence, such as an isoform-specific peptide, due to the identification of additional non-tryptic peptides derived from orthogonal proteases to support their confident identification. The percent of “rescued” and “resolved” protein isoforms whose presence were confirmed, or “validated”, in the multi-protease protein inference results can be calculated. This percent validation rate, when compared to expected rates, indicates how well the “rescue” and “resolve” portions of the “R&R” algorithm do at increasing the number of true positive protein isoform identifications.*

*Since the multi-protease protein inference results are still incomplete and subject to error due to incomplete peptide coverage of the proteome, the percent validated means very little on its own. To assess the significance of the percent of “rescued” or “resolved” isoforms validated, the experimentally determined value can be compared against the validation rates expected at random. Such values can be computed by calculating the percent of protein isoforms validated for a pool of randomly “rescued” or “resolved" protein isoforms.”*

*To address the question whether “the "resolved" transcript that gets removed is truly absent and how might one validate that?”, we performed an additional analysis, where we took all isoforms that were removed based on resolving protein groups, and looked for their identification in the multi-protease protein inference results. Only 0.7% of these isoforms were identified in the multi-protease protein inference results, which is significantly lower than the validation rate of the random results, and the validation rate of the resolved isoforms.*

*We have added this information to Figure 6e and added the following text to the Results (page 20):*

*“ We also investigated the validation rate of the protein isoforms that were removed from the protein groups, to determine if their removal was justified. We observed that only 0.7% of the removed isoforms were confirmed to be expressed in the multi-protease data. This is much less than the average fraction of “resolved” proteins validated from the distribution of the randomized control and the validation rate of the experimentally “resolved” protein isoforms (Fig. 6e).”*

*We have also modified Figure 6e to show the validation of the removed isoforms:*

*It is very difficult to validate the absence of an isoform based on the incomplete nature of proteomic data; however, this computational approach shows that the “resolve” portion of the “Rescue & Resolve” algorithm performs well in the context of isoforms that can be generally identified via bottom-up proteomics. To address the reviewer’s final question, we examined the 16 isoforms that were identified as a single isoform protein group in the multi-protease protein inference results to see if there was a bias in validation for isoforms with high transcriptional abundance, despite being a small fraction of the abundance for the whole protein group. We found that only one isoform had an abundance value over 10 CPM and that 13 of the 16 isoforms had CPM below 2. There does not appear to be any difference in inference performance based on total transcript abundance.*

**5**. Page 12 states that "protein isoform groups distinct to the protein inference results account for 382 cases of differing protein group composition.

a. I would suggest rewording this sentence for clarity. At the moment it is difficult to understand what the authors mean. Do they mean there are 382 instances where the incorporation of PacBio data led to a resolution of unresolved protein groups in GENCODE?

***Response:*** *To address the reviewer’s concern, we have rewritten the sentence of concern as well as the previous sentence.*

*The revised prose is found in the Results (page 13):*

*“A third scenario, accounting for 382 cases (12%), occurs when all of the protein isoforms for a protein group in the PacBio-Hybrid analysis are absent from any protein groups within the GENCODE reference database analysis. This results in a protein group that is entirely distinct to the PacBio-Hybrid protein inference results. An example, of this can be found in Figure 4f, where the PacBio-derived database lists a single isoform which is not found in the reference database, representing a case of an entirely distinct isoform model.”*

**b.** How many of these are found through unique isoform peptides not mappable to GENCODE, and how do these results correspond to the later section (page 14) that found 16 novel peptides?

***Response:*** *We thank the reviewer for this question. Of the 382 protein groups “distinct” to PacBio analysis, 193 or 50.5% of them have at least one unique peptide that maps to the protein group. A unique peptide in this context is a peptide that can only result from the digestion of a single protein isoform from the analyzed database. In total there are 581 unique peptides that map to these 193 protein groups. Many of these peptides are found in the GENCODE database as well, mapping to different or multiple protein isoforms. However, there are 47 unique peptides that are not found in an in-silico digestion of the GENCODE protein database. Not all of these peptides meet our stringent criteria to be considered confidently identified novel peptides. Six of the 47 unique peptides meet our criteria for novel peptides, and are included in our 14 identified novel peptides. It is important to note that the other 8 novel peptides are not unique to a single isoform and are considered to be shared peptides for the purpose of protein inference.*

**6.** In Figure 3d, how many novel isoforms are in the 69% genes within 'partial overlap' category, and what is the percentage of genes in belong to the "Superset" and "Distinct" isoforms from PacBio data categories? From the descriptions in the manuscript one might have assumed that there many pNNC events containing novel isoforms not in catalog (pg. 8 line 45) and hence presumably contain novel coding exons, but the protein ID results show that the peptide IDs are largely the same between GENCODE and PacBio. Does that mean none of those 20000+ pNNC events code for proteins?

***Response:*** *We thank the reviewer for their questions regarding the differences in isoform models between the PacBio-derived database and GENCODE reference database.*

*To address the first question, as to how many novel isoforms belong to the 69% of genes categorized as “partial overlap”, and the percentage of genes that belong to the “Superset” and “Distinct” categories, we added the following sentences to the Results (page 10):*

*“A total of 19,838 novel isoforms belong to genes in the “Partial Overlap” category. The other database-sample discordance categories which contain novel PacBio isoforms, “Superset” and “Distinct”, account for 8.9% and 3.1% of the genes in the database, respectively.”*

*Regarding the second question, we have the following response. There are 21,095 ORF calls categorized as pNNC, but not all of these isoforms make it into the final PacBio-Hybrid database due to quality filtering. In the protein database used for proteomic analysis, there are 16,384 protein isoform entries that are characterized as pNNC. Novel pNNC protein isoforms do have isoform-specific regions that result from events such as the presence of novel coding exons, however, they also have large regions that overlap with other protein isoforms. Due to the nature of proteomic sequence coverage and sensitivity, it is more likely that we observe shared peptides whose abundance may be contributed to by multiple protein isoforms than unique peptides from isoform-specific regions. This can explain why peptide identifications are largely the same when comparing the analysis of the GENCODE reference database to the PacBio-Hybrid database. However, in our proteomic data we do have evidence that several pNNC protein isoforms are expressed within the sample. First, we can investigate all the protein isoform mapping possibilities for all peptides identified in the PacBio-Hybrid analysis. We found that 75.8% (12,423) of pNNC isoforms present in the database have peptides that were identified that could map to them, prior to trimming based on protein inference results. Following protein inference, there are a total of 3,180 pNNC protein isoforms (19.4% of those in the database) which compose 1,841 protein groups that are identified at 1% protein FDR. Many of these isoforms are identified solely by the presence of shared peptides, but 126 pNNC isoforms were identified with unique peptide support, making their expression even more certain.*

**7.** As the authors pointed out, "Stringent validation criteria must be applied for novel peptide identifications" (page 14 line 19) but the criteria for such validation are not well defined in the manuscript and only manual examination is mentioned. Manual examination by trained experts can of course give some indication of how well the PSM is in terms of number of matched and unmatched peaks and peak series, but one would assume it does not give an indication of how good the peptide-spectrum match in question is when compared to other potential sequence matches in the databases.

**a.** Please clarify what were the predefined criteria for manual validation and whether any blinding was involved.

***Response:*** *This comment is appreciated as it has called attention to our need to be explicit and transparent in our manual annotation analysis.*

*Novel peptide identifications must have met the MS search algorithm criteria applied to all peptides. In addition, Human Proteome Project MS data guidelines (J Proteome Res. 2019 Dec 6; 18(12): 4108–4116) for data-dependent acquisition spectra were applied: high mass accuracy (5 ppm precursor ion and 20 ppm product ion mass error), clearly annotated spectrum that was scrutinized for missed and extra peaks (we used a rough threshold of 25% maximum of unassigned fragment ions above 10% relative abundance) and peptide length (minimum of 9 amino acids). Also, mass errors of novel peptide hits were checked for consistency with other valid hits from the same raw file. Extra weight was given for the presence of sequence-specific characteristics (if a highly charged peptide was longer or had higher number of basic residues; if y-ions ending in proline were higher intensity than other fragment ions). Precursor co-isolation can complicate annotation by resulting in fragment ions from multiple peptide origins. This complexity was taken into account by noting the percentage of MS2 total ion count (TIC) accounted for by annotated fragment ions. This percentage is represented by the decimal digits of the MetaMorpheus score. TIC coverage of 20% or more was used as a rough threshold but not a hard criterion. Finally, we confirmed the novel peptide assignment had better MetaMorpheus, q-value, and PEP scores than any GENCODE reference hits for the same spectrum. We have created a new supplemental note (now Supplementary Note 4) where all criteria for novel peptide validation is explicitly stated.*

*To address the need for explicit delineation of novel peptide annotation criteria, we have created a completely new Supplementary Note 4 (page 8-10):*

*“The identification of novel peptides requires rigorous validation and stringent filtering criteria to ensure that the spectrum does in fact represent the novel peptide sequence. First, all novel peptide identifications must meet the basic filtering criteria for all peptide identifications, having a minimum MetaMorpheus score of 5 and being present at a global 1% FDR cutoff. The distribution of the FDR, or q-values, for the novel peptide identifications was compared to the FDR, or q-value, distribution of the canonical peptides (Fig S4.1). The median q-value for the novel peptides was lower than the median q-value for the canonical peptides with values of 0 and 2.1x10-5, respectively. Since the FDR, or q-value, is a global assessment of confidence for the entire set of peptide identifications, not a confidence metric for the specific peptide identification, the posterior error probability (PEP) of each novel peptide was also considered. All novel peptides had a PEP value less than 0.005. The distribution of novel peptide PEP values was compared to the distribution of PEP values for all canonical peptide identifications (Fig S4.1). The median PEP value for the novel peptides and canonical peptides were nearly identical, demonstrating the novel peptide identifications are as confident as the canonical peptide identifications.*

*In addition to the preliminary filtering and confidence evaluations of the novel peptide identifications, the Human Proteome Project MS data guidelines[5] for manual validation of data-dependent acquisition spectra were applied. These criteria include: 1) high mass accuracy (5 ppm precursor ion and 20 ppm product ion mass error), 2) clearly annotated spectrum that was scrutinized for missed and extra peaks (we used a rough threshold of 25% maximum of unassigned fragment ions above 10% relative abundance) and 3) peptide length (minimum of 9 amino acids).*

*Additional manual validation criteria were applied to each novel peptide identification. Mass errors of novel peptide hits were compared against the mass error of other confident spectral assignments from the same raw file to check for consistency. Extra weight was given for the presence of sequence-specific characteristics. For example, the spectra assignment was considered more confident if a highly charged peptide was longer, had a higher number of basic residues; or if y-ions ending in proline were higher intensity than other fragment ions. Precursor co-isolation can complicate spectral annotation by resulting in fragment ions from multiple peptide origins. This complexity was taken into account by noting the percentage of MS2 total ion count (TIC) accounted for by annotated fragment ions. This percentage is represented by the decimal digits of the MetaMorpheus score. TIC coverage of 20% or more was used as a rough threshold but not a hard criterion.”*

**b.** How do the q values/FDR of these matches compared to the q value distributions of canonical peptide PSMs?

***Response:*** *We thank the reviewer for their question regarding the q-value of novel peptides compared to canonical matches. To address this, we generated a plot comparing the distribution of q-values for novel peptides to those of canonical peptides. The plot can be found below. The distribution of the q-values of the novel peptides is better than that observed for all other canonical peptides. The median q-value for the novel peptides (red dashed line) was 0, whereas the median q-value for the canonical peptides (blue dashed line) was 2.1E-5.*

*To expand on the reviewers comment we also generated a plot comparing the distribution of PEP values for the novel and canonical peptides. As can be observed in the plot below, the median PEP values, as represented by the dashed lines, are nearly identical.*

*These analyses further support the strength of our novel peptide identifications. These plots have been added to Supplementary Note 4 as Figure S4.1.*

*We also added text to Supplementary Note 4 (pages 8-9):*

*“The distribution of the FDR, or q-values, for the novel peptide identifications was compared to the FDR, or q-value, distribution of the canonical peptides (Fig S4.1). The median q-value for the novel peptides was lower than the median q-value for the canonical peptides with values of 0 and 2.1x10-5, respectively. Since the FDR, or q-value, is a global assessment of confidence for the entire set of peptide identifications, not a confidence metric for the specific peptide identification, the posterior error probability (PEP) of each novel peptide was also considered. All novel peptides had a PEP value less than 0.005. The distribution of novel peptide PEP values was compared to the distribution of PEP values for all canonical peptide identifications (Fig S4.1). The median PEP value for the novel peptides and canonical peptides were nearly identical, demonstrating the novel peptide identifications are as confident as the canonical peptide identifications.”*

**c.** Since FDR in proteomics typically refers to a property of the entire set of admitted results, did the authors look at the local FDR or posterior error probability of the individual peptide spectrum matches?

***Response:*** *MetaMorpheus includes a PEP value for each individual peptide spectrum match (PMID: 33683901). All of the PEP values for the novel peptides are less than 4.14 e-3, with 13 out of 14 less than 2.4 e-4. The PEP value for each novel peptide has been added to Supplemental Table 4.*

**d.** Were post processing algorithms like Percolator or Peptide Prophet used to authenticate and/or re-rank the PSMs?

***Response:*** *No post-processing algorithms were used to re-rank PSMs. However, as mentioned in the previous response MetaMorpheus calculates a PEP value for each PSM, it also calculates a q-value based on this PEP. When we re-rank the PSMs by this PEP q-value we observe that all of the novel peptides remain at 1% FDR. The long-read proteogenomics pipeline is flexible, and semi-supervised learning tools such as percolator and mokapot could be easily incorporated into the proteomic analysis portion of the pipeline.*

*We have added text to the Discussion (page 22):*

*“For example, the proteomic analysis portion of the pipeline could be expanded to include a semi-supervised learning post-search program such as percolator[55] or mokapot[56].”*

**e.** What were the isoform PSM spectra matched to in a regular database search and how did their corresponding q values compared to searching with the Proteogenomics database?

***Response:*** *Spectra were searched against the GENCODE reference database and scan numbers that matched PSMs for novel hits were scrutinized for MetaMorpheus Score, q-value, and PEP score. In all cases the novel peptide PSM provided superior scores versus the GENCODE hits. Supplemental Table 4 was edited in the following manner: 1) four columns were added to reflect additional validation scrutiny: “PEP score”, “Meets peptide length minimum of 9AA (from HPP Data Guidelines v3.0.0”, “Checked neXtProt for uniqueness (Bioinformatics 33(21):3471-72)” and “Did search against GENCODE result in better scoring hit for this MS/MS scan?” and 2) two columns were added to provide raw spectrum information: “raw file name (in Peptide Atlas PASS00215 repository)” and “MS/MS spectrum scan number”.*

*This analysis was also included in the newly added Supplementary Note 4 (page 10):*

*“We also investigated whether any peptide sequences from contaminant proteins, GENCODE reference isoforms, single amino acid variant containing proteins or post-translationally modified proteins could provide better peptide assignments for the novel peptide spectra. Identifications were evaluated based on MetaMorpheus score, q-value and PEP. A contaminant protein database, included in MetaMorpheus, was searched alongside the PacBio-Hybrid database. No contaminant peptides were a match to the novel peptide spectra. In the search of the GENCODE reference database, no peptide identifications for the spectra in question were a better match than the novel peptides (Supplementary Table 4).”*

**f.** Did the authors examine whether the spectra could be explained by other means like SAAV, contaminant proteins from other species, or unknown PTMs (as discussed in Neshvizhskii Nat Methods 2014 and elsewhere)?

***Response:*** *We thank the reviewer for this astute comment regarding alternative origin sources for the novel peptides. All proteomic searches performed within the pipeline using MetaMorpheus also searches against a curated contaminant protein database, as described on page 30-31 in the manuscript. Using this contaminant database there are no proteins from other species that provide equivalent, or superior matches for the novel peptide spectra in question. To address the possibility of post-translationally modified canonical peptides matching to the novel peptide spectra, we performed GPTMD using MetaMorpheus (PMID: 28248113 and PMID: 29578715) followed by a search to identify post-translationally modified peptides. No modified peptides matched to the spectra for the novel peptides at 1% FDR. We also searched the proteogenomic database generated by Spritz utilized in the following publication (PMID: 32967423), to identify whether peptides resulting from SNVs, MNVs, small indels, frameshifts, or other types of variations are better matches for the novel peptide spectra.*

*We have added text to the newly created Supplementary Note 4 describing the results of the PTM and variant analyses on page 10:*

*“We also investigated whether any peptide sequences from contaminant proteins, GENCODE reference isoforms, single amino acid variant containing proteins or post-translationally modified proteins could provide better peptide assignments for the novel peptide spectra. Identifications were evaluated based on MetaMorpheus score, q-value and PEP. A contaminant protein database, included in MetaMorpheus, was searched alongside the PacBio-Hybrid database. No contaminant peptides were a match to the novel peptide spectra. In the search of the GENCODE reference database, no peptide identifications for the spectra in question were a better match than the novel peptides (Supplementary Table 4). To search for post-translationally modified peptides, Global Post-Translational Modification Discovery (GPTMD), with default settings, was performed with the GENCODE reference database [6]. The subsequent search found no modified peptides were a match for the novel peptide spectra. To investigate if variant containing peptides could better account for the spectra supporting the novel peptide identifications, a proteogenomic database generated by Spritz using Jurkat short-read RNA-seq data published by Cesnik et. al. [7] was searched. The results showed that no variant containing peptides were identified for the same spectra as the novel peptide assignments. Based on this stringent evaluation of novel peptide candidates, we are quite confident in the 14 novel peptide assignments that passed all criteria.“*

**g.** Some of the peptides (GELDGELR and ELAGEAR) are relatively short and so carry higher chance of misidentification. I would strongly recommend the authors adhere to the HPP guidelines of at least 9 amino acids for "extraordinary detection claims" of novel peptides (Deutsch et al. J Proteome Res 2016) and disregard them from consideration.

***Response:*** *We agree with the reviewer’s suggestion to adhere to the HPP guidelines of at least 9 amino acids for claims of novel peptides and have edited our manuscript and Supplemental Table 4 to remove the two novel peptides that were less than 9 amino acids long.*

**8.** I was somewhat surprised that only a few unique novel peptides were detected (16 peptides), this seems to run counter to the results of some existing short-read sequencing based proteogenomics studies.

***Response:*** *The reviewer brings up a good point, and we believe that several factors are at play in producing the somewhat lower number of novel peptides as compared to other proteogenomic studies.*

*First, we employed stringent filtering (i.e., manual annotation) to ensure that all the novel peptide identifications passed only the most conservative thresholds. Novel peptide identifications reported in past proteogenomics, particularly when the field was at its infancy, are considered to more likely exhibit an elevated false positive rate (PMID: 25357241, PMID: 20620248). Therefore, we decided to apply conservative thresholds in our dataset out of an abundance of caution.*

*Second, sample-specific isoforms were not included for all genes, as 6,653 of the ~20K human genes contained PacBio transcripts (“PacBio-Hybrd” database). It is possible that for some of the lower abundance genes, we might have detected novel peptides corresponding to lower abundance novel isoforms.*

*Third, we ensured that the novel peptides were not found in either GENCODE or UniProt. GENCODE and UniProt differ in the isoforms annotated, and we found some peptides that were novel in terms of comparison to GENCODE, but not to both GENCODE and UniProt.*

*And, fourth, Jurkat cells are a well-studied cell line and splicing variations it expresses may be more likely included in reference protein databases than rarer cell types. This comment is more speculative in nature, but we would suspect we’d find many more novel isoforms in rare cell populations or poorly studied disease-associated specimens.*

*Lastly, it is difficult to confirm the expected number of novel peptides for any given sample, but large-scale proteogenomics studies in healthy and cancer tissues may give some indication of expected coverage. For example, in a seminal CPTAC study, a range of 24 to 64 novel spliced peptides were detected per tumor sample, with a median of 41 across the whole dataset (PMID: 27251275). These samples represent a more complex biological source, including many cell types represented in a breast cancer tumor, and we suspect a higher number of novel peptides would be found. Furthermore, the authors of the study used RefSeq annotations, which tend to be more conservative and comparisons against it may lead to higher number of novel peptides. Note that the number of variant peptides (arising from genetic variants such as single nucleotide polymorphisms) is far higher, with typically a median of ~600 to 700 variant peptides identified per cell line in this same study.*

**a.** What is the depth of the mass spec experiments and the predicted percentage of peptides that were mappable to non-canonical isoforms, regardless of whether they exist in GENCODE?

***Response:*** *The proteomic dataset, as mentioned in the manuscript on page 11 and page 26, is heavily fractionated with high pH offline producing 28 fractions, which greatly increases the depth of coverage obtained relative to what could be achieved without fractionation. We feel that the depth of coverage achieved was relatively high given the mass spectrometers available at the time of data acquisition. The mass spectrometer used to collect the data was a Thermo Velos Orbitrap, so advances in MS technologies have led to release of newer instruments with increased duty cycle, resolution, and overall achievable depth of coverage of the proteome, such as the Thermo Fusion Lumos Tribrid or the Thermo Eclipse Tribrid.*

*To answer the reviewers question we performed three different analyses based on different interpretations of what a non-canonical isoform is. The three different definitions are as follows: 1) all novel isoforms (pNNC or pNIC); 2) all pFSMs or GENCODE accessions that are not the APPRIS reference (analogous to UniProt canonical protein isoforms); and 3) all isoforms that are not the APPRIS reference (non-APPRIS pFSMs and GENCODE accessions as well as pNIC and pNNC). The percent of the 75,750 peptides identified at 1% FDR that were mappable to non-canonical isoforms for each of the definitions are summarized below:*

1. *43,564 peptides (58%) were mappable to novel protein isoforms (pNNC or pNIC).*
2. *40,914 peptides (54%) were mappable to non-APPRIS pFSMs and GENCODE protein isoforms.*
3. *63,143 peptides (83%) were mappable to all isoforms that are not APPRIS ( non-APPRIS pFSMs and GENCODE accessions as well as pNIC and pNNC).*

**b.** Currently the manuscript only mentions in passing "Many of these proteomic variations are difficult to identify from short-read RNA-seq datasets". As already suggested above, I would recommend the authors actually compare with a current proteogenomics workflow, i.e., using a database generated by short-read sequencing, to contrast the number isoform peptides identifiable in the mass spec data, which would allow the authors to determine whether there is improvement over existing workflows or alternatively attempt a "post-mortem" analysis of where some of the isoforms are lost.

***Response:*** *We agree with the reviewer that to make the suggestion that novel peptide identification is improved over short-read RNA-seq datasets, we should have provided results indicating this is the case. Anecdotally, we have observed such issues; but these case-by-case observations were not backed up through a systematic analysis. In the task of detection of “local” novel events (e.g., splice junctions, novel exons; not full-length isoforms), short and long-read RNA-seq datasets are likely highly comparable, but it is very difficult to compare full-length isoform structures and the relationship to shared and unique peptides.*

*Accordingly, we removed several sentences from the Results (page 14):*

*“Many of these proteomic variations are difficult to identify from short-read RNA-seq datasets. For example, intron retention events are typically filtered from short-read data as presumably incompletely spliced products, and short transcript fragments make the confident delineation of ORF boundaries difficult.”*

**9.** The description on page 15 would seem to fall short of a timely overview of the existing start of the art in protein inference algorithms. ProteinProphet for example allows sister peptide features to be turned off and hence a unique peptide should lead to a unique protein IDs, whereas other inference algorithms such as implemented in Percolator/Fido would directly assess the probability that a protein can emit an observed PSM.

***Response:*** *We agree that the text in question does not provide an overview of all existing protein inference algorithms. We intended to introduce one of the models used for protein inference, the parsimonious model, and described some of its limitations, especially in the context of protein isoform inference. However, as the reviewer points out, the original manuscript text was too restrictive in describing only the parsimonious inference approach without at least a passing mention of probabilistic and other potential methods in the field.*

*We added text to the Results (page 15), and have included additional references to reviews and recent publications describing other protein inference algorithms:*

*“In order to infer the presence of protein isoforms, most protein inference algorithms employ a probabilistic or parsimonious approach. Probabilistic protein inference algorithms seek to estimate the probability that a given protein isoform is in the sample on the basis of the peptides observed[42–45]. Parsimonious protein inference algorithms are more heuristically driven and follow Occam’s razor, which attempts to define the smallest number of protein isoforms that “covers” the set of identified peptides[9,45–49].*

*Parsimonious algorithms are commonly used in the MS proteomics field as part of search software platforms like Andromeda/MaxQuant and MetaMorpheus. However, this approach can lead to elimination of bona fide protein isoforms that lack sufficient peptide support relative to other isoforms (Figure 6a)[50]. Alternative isoforms are particularly susceptible, because their isoform-specific regions comprise a small fraction of the proteome and suffer from a negative detection bias in traditional MS-based proteomics workflows using tryptic digestion[51].”*

*References which were added (#50 and #51):*

*Searle BC. Scaffold: a bioinformatic tool for validating MS/MS-based proteomic studies. Proteomics. 2010 Mar;10(6):1265-9. doi: 10.1002/pmic.200900437. PMID: 20077414.*

*Wang X, Codreanu SG, Wen B, Li K, Chambers MC, Liebler DC, Zhang B. Detection of Proteome Diversity Resulted from Alternative Splicing is Limited by Trypsin Cleavage Specificity. Mol Cell Proteomics. 2018 Mar;17(3):422-430. doi: 10.1074/mcp.RA117.000155. Epub 2017 Dec 8. PMID: 29222161; PMCID: PMC5836368.*

**10.** How much of the non-overlap between GENCODE and PacBio transcripts can be attributable to the limitations of the CPAT ORF prediction algorithm, for example not able to find alternative N termini or translation starts?

***Response:*** *This is a good question and prompted by this question, we realized that it would be prudent to include in the manuscript some information about the extent to which ORF prediction from reference (i.e., GENCODE) transcripts would lead to the same protein isoform predicted.*

*As the reviewer describes, novel protein isoforms could arise from minute changes on the selection of start ATG (i.e., initiator codon). The CPAT ORF calling has a high rate of recapitulating GENCODE proteins; however, we acknowledge that the success rate is not 100%. Overall, it is challenging to predict ab initio any non-canonical ATG start sites, downstream ATG start sites (e.g., when the first ATG is not optimal or lacks a Kozak consensus), or variations in the stop codon, such as stop read-through or recoding events such as selenocysteines. Though capturing such non-canonical ORF boundaries is beyond the scope of the paper, it is an important limitation of our approach. Therefore, we made edits to call more attention to the issue.*

*We added text to Supplementary Note 2 (page 5-6):*

*“In order to determine whether our ORF calling algorithm is able to recover ORFs annotated in GENCODE, we compared the GENCODE reference ORFs (ENSPs, i.e., GENCODE proteins) versus the ab initio CPAT-predicted ORFs. We found that of the 58,860 GENCODE transcripts, the CPAT-predicted ORF matched the ORF annotated by GENCODE in 55,324 or 94% of cases. For 3,536 or 6% of cases, the ORF was not an exact match (Fig S2.4). A majority of the ORFs that differ from the reference differ due to differences in the N-terminus, with 55% differing only in the ATG start location. Note that in our proteogenomics pipeline, as part of our ORF calling procedure, we heavily weigh the presence of a GENCODE ATG; therefore, a majority of these cases would be reverted to the GENCODE ORF within our pipeline. For 60 cases (0.1% of the dataset), only the C-terminus did not match, and a majority of such cases could be explained by selenocysteine recoding events, in which the stop codon is recoded to selenocysteine, thereby extending the protein C-terminus.”*

*We added a figure to Supplementary Note 2 (page 6):*

***Minor comments:***

**11.** Figure 1 looks somewhat confusing to this reviewer. In Figure panels 1b-1f, one assumes the authors meant for the blue and red boxes to refer to Isoform sequences in reference database vs. isoform sequences in sample-specific databases, as the actual protein isoforms in a sample may differ from both. Some of the "consequence" of using reference vs. sample-specific database should be better clarified, for example, because sequencing sensitivity and specificity are not perfect, when the sample-specific database is a subset of the reference database the identified specific isoform may be a false positive.

***Response:*** *The reviewer’s feedback on Figure 1 is appreciated. Our intention was for the blue boxed isoforms to represent isoforms in a reference database and the red boxed isoforms to represent bona fide isoforms expressed in a sample, not a sample-specific database.*

*In response to the reviewer’s comment we have changed the location of the key for panels b-f from the bottom to the top, to make clear what the boxes stand for when the reader is interpreting the figure. A copy of the revised figure panels can be found below.*

*To clarify the definition of isoforms in the blue versus red boxes, we have added text to Figure 1, underlined:*

*“****b****, The assumption of MS-based proteomics search algorithms is that the reference and sample isoforms match. Isoforms in the light blue boxes represent those annotated in a reference database. Isoforms in the light pink boxes represent isoforms that are actually expressed in a sample (which is unknowable using current technologies).”*

*To address the reviewer’s concern regarding the “consequences” of using the reference vs sample-specific database we have added text to the manuscript Background (page 4). The revised text can be found below:*

*“Transcript sequencing can be used to generate a sample-specific candidate protein database., which is more reflective of the isoform diversity in the sample than the reference database, but still has limitations due to the sensitivity and specificity of sequencing technologies. Presently, such efforts to generate sample-specific databases have been dominated by using short-read RNA-seq[11–20] which suffers from the inability to sequence full-length transcripts and can only deliver partial protein models[21,22](Fig. 1g).”*

**12.** Given the major finding here is the differences between GENCODe and PacBio-hybrid transcripts rather than identification of novel peptide, one wonders if "enhancing protein isoform inference using long-read sequencing data" would make for a more accurate title for the manuscript.

***Response:*** *We thank the reviewer for their suggestion. We believe that although the manuscript deals with the problem of isoform inference, there is much more than the protein inference process that it describes. Emphasizing inference alone in the title may cause the important work in this paper such as the development of a protein isoform classification schema, and the open access and extensible long-read proteogenomics software pipeline to be overlooked. Due to these reasons, we have decided to leave the title of the manuscript as is.*

**Reviewer #2 Comments:**

This manuscript "Enhanced protein isoform characterization through long-read proteogenomics" reports a new method for the incorporation of long-read DNA sequencing for transcriptome and proteome analysis. This is an important problem. The approach is novel and carefully developed. The manuscript is well written, but can be improved to clarify the performance of the method.

***Response:*** *We thank the reviewer for the positive comments.*

***1)*** The manuscript uses PacBio sequencing of Jurkat cells. It is generally agreed and established from prior studies that long-read sequencing is superior than short-read (Illumina) sequencing for transcript and isoform detection. However, it is not clear whether the current data of Jurkat cells are sufficiently sampled to detect most expressed transcripts. The data can be sub-sampled to show the dependency of unique transcript detection on sequencing converge. This would be important to demonstrate adequate sensitivity.

***Response:*** *This is an excellent suggestion. Understanding of the extent to which the long-read RNA-seq data can fully sample the transcriptome space, and the point at which saturation is achieved is both interesting and practical knowledge for “long-read proteogenomics”.*

*To explore the relationship between sequencing depth and isoform/gene detection, we generated saturation-discovery curves (see below) in which we incrementally subsampled from the full-length reads and determined the number of isoforms/genes recovered. For the subsampling, we randomly drew a subset of reads and the number of unique genes or transcript isoforms detected was determined. For each sampling depth, 100 iterations of sampling was done and the average number of unique genes or isoforms observed was computed. The saturation-discovery curve is done using the `subsample.py` and `subsample_with_category.py` scripts in* [*https://github.com/Magdoll/cDNA_Cupcake*](https://github.com/Magdoll/cDNA_Cupcake)*.*

*Similar to previous studies (PMID: 32393825,* [*https://www.biorxiv.org/content/10.1101/672931v2*](https://www.biorxiv.org/content/10.1101/672931v2)*), we found that the number of genes plateaus after roughly 1 million full-length reads sampled. The number of isoforms gradually increases between 0 and 3.5 million reads, and appears to begin plateauing at 4 million reads, although additional datasets should be collected to confirm this trend.*

|   *Rarefaction curve - Vertical axis is log10 scale* |   *Rarefaction curve* |
| --- | --- |

*We added text, underlined here, to the Results (page 6), as well as a new panel in Supplementary Figure 2, renumbering the subsequent figure elements accordingly:*

*“In 13.93% (1,274) of genes, the most abundant transcript isoform is novel. Note that for this study, transcript nucleotide sequences were derived from the reference genome (genome-corrected mode in SQANTI3), therefore genetic variations are not captured in the current version of our pipeline (see Discussion). To determine the sampling sensitivity of the transcriptome, we generated saturation-discovery curves and confirmed that the number of unique genes and isoforms detected reaches a plateau (Supplementary Fig. 2b). Overall, these results illustrate the widespread nature of alternative splicing and the need for empirically driven methods to characterize isoform diversity in human samples.”*

**2)** It is known that PacBio sequencing has a not insignificant base error rate, which is higher than current Illumina sequencing. If matched Illumina cDNA sequencing data is available, a direct comparison to estimate the sequencing error rate and its impact on the mass spectral matching should be done: in other words, does the current method underestimate alternative isoforms. This may be inferred from the analysis of mass spectral sequence tags or spectra identified de novo to determine the influence of transcript sequencing error rate.

***Response:*** *The reviewer is correct that the base error rate for PacBio sequencing is high, typically 10-20% per base pair. However, for transcriptome sequencing (“Iso-Seq” as described by PacBio), the data represents circular consensus sequenced (CCS) reads, which had an average error less than 0.1-1% (Phred score 20-30, described in PMID: 34508117, PMID: 25150838, and other publications).*

*For a majority of our reads, the error rate is of comparable accuracy to Illumina sequencing. With that said, even for an error rate of less than 0.1% is actually not accurate enough for the purpose of predicting full length ORFs. This is because a single insertion or deletion error could potentially shift the frame of translation and create an artifactually truncated ORF.*

*Therefore, out of an abundance of caution, we decided to use genome-corrected sequences. This was accomplished by aligning the clustered transcript isoform sequences to the reference genome (hg38) and—based on the exon-intron structures—extraction and concatenation of the genome-based exonic sequences to reconstitute a “corrected” transcript sequence. These steps were performed within SQANTI3 using minimap2 and custom scripts within SQANTI. Because we used genome-corrected sequences, the rate of sequencing errors propagating to the database is likely low. However, because we used genome-corrected sequences, all sample-specific genetic variants are masked, and variant peptides that differ from the reference genome (hg38) cannot be detected in our method.*

*We are currently working on a manuscript to efficiently extract genetic variant information directly from long-read data (PacBio, ONT) for proteogenomic search. After submission of our manuscript, a report published in the Journal of Proteome Research described an approach to call genetic variants from Nanopore reads and use of the database in combination with an open MS search approach to detect variant peptides (PMID: 33998808).*

*The masking of variant sequences may have been unclear in the text; therefore, we have made edits, underlined, in the manuscript to make this more clear (Results, page 7):*

*“Note that for this study, transcript nucleotide sequences were derived from the reference genome (genome-corrected mode in SQANTI3); therefore, genetic variations are not captured in the current version of our pipeline (see Discussion).”*

**3)** ORF prediction is a key element of the method. The choice of CPAT seems reasonable, but a direct comparison with TransDecoder or another suitable algorithm is warranted.

***Response:*** *Like the reviewer, we considered comparing different ORF calling algorithms. We compared CPAT with TransDecoder (PMID: 23845962) and GMST (PMID: 25870408). We found that the majority of ORFs called were in common between all three ORF callers, although each algorithm did produce a distinct set of ORFs, as expected, based on different assumptions and scoring metrics of each algorithm.*

*We added text to Supplementary Note 2 (page 4) that shows the overlap:*

*“For calling of ORFs from full-length transcript isoforms, we used the CPAT algorithm. Several ORF callers are available. We compared the identity of ORFs called using CPAT versus TransDecoder[2] and GMST[3]. To run TransDecoder (version 5.5.0), a minimum ORF size was set to 50 nucleotides to mimic the parameters we used for CPAT. The single best ORF for each isoform was selected, per the `Transdecoder.Predict` parameter. To run GMST the same parameters as used in the SQANTI pipeline (GMST version 5.1) were used. We found that in a majority of cases, the same ORF is predicted; however, there are some differences in ORFs called.”*

*In addition, we added the figure and legend below:*

*Figure S2.1: Comparison of ORF callers in predicting ORFs from full-length transcripts (PacBio).*

***4)*** The manuscript compares the results of mass spectral searches against PacBio-derived database and a database containing PacBio-identified transcripts and GENCODE transcripts. How many proteins and isoforms are identified by searching PacBio-derived database exclusively? How does this relate to question #1.

*Response: When searching the PacBio-derived database exclusively, we detected 7305 protein groups from 7068 genes and a total of 70,761 peptides. We inadvertently omitted these values, and thank the reviewer for pointing this out.*

*We added text, underlined here, in Supplementary Note 3 page 6 :*

*“To determine factors underlying incomplete proteomic coverage using the PacBio database, we examined the properties of genes in which there were fewer peptides recovered when using the PacBio database than when using the GENCODE database. When searching the PacBio database (PacBio only), we detected 70,761 peptides and 7,068 genes, which corresponds to 90.7% of peptides and 92% of genes detected using the GENCODE database. Overall, lower peptide recovery was observed for genes with extreme transcript lengths (e.g., less than 1 kb, longer than 4 kb) or very low abundance (e.g., below ~3 CPM) (Fig. S3.1).”*

*Based on the rarefaction analysis, we find that the transcript isoform and gene detection rates start to plateau at the read coverage we obtained in our study. However, based on the proteomics results, we find that the sampling of the proteomic space is not 100%. However, based on analysis we did for question #1, we found that the gene and isoform detection rates start to plateau, which suggests that with collection of more data, that protein detection could improve. As described in Supplementary Note 3, we believe that lower gene abundance and longer lengths of gene products are more difficult to sample.*

**5)** For the novel transcript isoforms detected using the method, how many have mass spectral evidence? Of the isoforms that lack mass spectral evidence, how many do so because of the absence of theoretically suitable peptide sequences (lack of enzyme digestion site, lack of charge, etc) versus the number that are theoretically expected, but not detected?

*Response: The question of MS coverage for protein isoforms translated from novel transcripts is very interesting. A total of 36,046 novel transcript isoforms compose 22,997 PacBio-derived protein isoforms in the searched PacBio-Hybrid database. As stated in the manuscript on page 9, novel transcript isoforms can produce reference protein isoforms when the novel components of the transcript are present in the untranslated regions. Therefore of the 22,997 protein isoforms derived from novel transcript isoforms, 18,187 are novel protein isoforms (pNIC and pNNC) and 4,810 are reference protein isoforms (pFSM).*

*These protein isoforms can have different levels of mass spectral evidence. The best form of mass spectral evidence is an isoform-specific unique peptide. Shared peptides can also provide mass spectral evidence as they could be the digestion product of a protein isoform in question. Of the 4,810 reference protein isoforms (pFSMs) from novel transcripts, 485 (10%) have unique peptide support and 3305 (69%) have shared peptide mass spectral evidence. For the 18,187 novel protein isoforms, 139 (0.7%) have unique peptide support and 14,307 (79%) have shared peptide mass spectral evidence.*

*For the protein isoforms from novel transcripts which lack mass spectral evidence, the results of in silico digestion were investigated using the software tool ProteaseGuru (PMID: 33661641). In silico generated theoretical peptides are considered identifiable if they are between 6 and 40 amino acids in length, and if they have no missed cleavages. First, proteins were investigated for theoretically identifiable unique peptides.*

*For the 4,325 pFSMs from novel transcripts without unique peptide support, 2,251 (52%) of the isoforms do not have an identifiable theoretical unique peptide. For the 18,048 novel protein isoforms without unique peptide support, 7,169 (39%) of the isoforms do not have an identifiable theoretical unique peptide.*

*Next, the protein isoforms were investigated for any theoretically identifiable peptides. For the 1,505 pFSMs and 3,880 novel protein isoforms without any mass spectral evidence. For the 1,505 pFSMs without any peptide evidence, 19 (1.2%) isoforms do not have any identifiable theoretical peptides. For the 3,880 novel protein isoforms without unique peptide evidence, 31 (0.8%) isoforms do not have any identifiable theoretical peptides.*

*More protein isoforms from novel transcripts which lack mass spectral support have low transcriptional abundance (less than 2 CPM) compared to the abundance of protein isoforms from non-novel transcripts with mass spectral support. 68% of protein isoforms from novel transcripts without unique peptide support have low transcriptional abundance, whereas only 6% of protein isoforms from non-novel transcripts with unique peptide support do. This trend is repeated for protein isoforms from novel transcripts without any peptide support, with 67% having low transcriptional abundance, compared to 43% for protein isoforms from non-novel transcripts. As we discuss in the paper, we hypothesize that protein isoforms with low transcriptional abundance are less likely to be expressed as protein, or expressed at levels within the range of sensitivity of mass spectrometry. It is unsurprising that the detection of unique peptides is more heavily correlated to transcriptional abundance than the detection of any peptides. It has been observed that higher abundance proteins are more likely to have unique peptide identifications.*

**6**) Is there either transcript evidence with or without mass spectral support for genes that are not currently annotated?

***Response:*** *As the reviewer has indicated, our pipeline does not examine transcripts or ORFs from novel genes, though it would be quite interesting if they exist.*

*The definition of novel genes within the PacBio-based Iso-Seq and SQANTI pipeline can be a misnomer as “novel genes” are bioinformatically defined based on sequence cluster positions. Within the pipeline, “genes” represent clusters of overlapping reads which do not overlap adjacent clusters. In other words a “gene” as a unit is a non-overlapping cluster of reads. If these clusters overlap known genes (protein-coding or lncRNA, as defined in GENCODE), the cluster is considered a “known gene”, and if there is no overlap, it is considered a “novel gene”. In our experience, we find that “novel genes” tend to be antisense transcripts or short transcripts that reside in the 3’ UTR of existing genes, rather than bona fide novel genes. However, within the group of “novel genes”, it is possible that a truly novel gene may be lurking.*

*We explored the possibility of detecting peptides against the “novel genes” detected in this pipeline, as the reviewer raises an intriguing point that our pipeline may be able to discover unannotated genes. To this end, we generated a list of 3,105 proteins predicted from transcripts mapping to “novel genes” and appended the list to the PacBio-Hybrid database (35,119 known gene PacBio entries, 48,413 GENCODE entries, and 3,105 novel gene entries) to create a database, PacBio-Hybrid-NovelGenes. The proteins predicted were derived from ORFs called used the exact same workflow as that for generation of PacBio-Hybrid database. We searched the PacBio-Hybrid-NovelGenes against the Jurkat trypsin dataset and detected 35 peptides at 1% FDR that map to novel genes. We found that 4 of the 35 peptides are shared between a novel gene and a known protein-coding gene. Overall, the peptides correspond to 7 protein groups, each a single isoform, which passes a 1% protein-level FDR, which is applied post-protein inference. Overall, we found that the PEP and q-values for these isoforms were quite low. Upon manual examination, none of the isoforms corresponded to a bona fide novel gene, but were, rather, mapping to antisense and UTR-overlapping transcripts.*

*In conclusion, we did not find evidence for completely novel genes at the transcript or protein-level.*

*To include the possibility of detection of novel genes, we added brief text, underlined, in the Discussion (page 22):*

*“The applications of our computational pipeline could also include the analysis of novel genes or genetic variation that is detectable in long-read data...”*

**7)** How many transcripts have features, such as exon-exon junctions or chimeric sequences, emanating from somatic DNA mutations, such as translocations, deletions etc, that are common in Jurkat cells?

***Response:*** *We thank the reviewer for this intriguing question. In our manuscript, we focused our analysis on isoform-related variation at the transcript level, as detectable through a long-read RNA-seq approach, which could have included some variations that arise due to genomic rearrangements, fusions, or other mechanisms that can occur in karyotypically abnormal cancer lines such as the Jurkat cells we used as a model system. Jurkat cells are tetraploid and we certainly cannot discount the possibility that the isoforms we detected are the result of genome-level fusions or other mutations. We feel that an extensive characterization of genetic mutations in Jurkat cells is beyond the scope of the paper; however, we felt it necessary, as the reviewer pointed out, to make this limitation of the method clear.*

*Accordingly, we added text, underlined, to the Discussion (page 23-24):*

*“Though long-read proteogenomics and its application hold promise, limitations remain. First, for the “Rescue and Resolve” approach, we assume at least a moderate degree of RNA-protein correlation. Although isoforms from the same gene should not greatly differ in their transcript-protein correlation, several studies have reported isoform-specific mRNA translation[63,64] suggesting that alternative splicing can generate transcripts with distinct cis-regulatory landscapes. Therefore, caution must be taken for any given protein isoform, including follow-up confirmation of expression in vivo.”*

*We cited this study of relevance to the points made above:*

*Gioia L, Siddique A, Head SR, Salomon DR, Su AI. A genome-wide survey of mutations in the Jurkat cell line. BMC Genomics. 2018 May 8;19(1):334. doi: 10.1186/s12864-018-4718-6. PMID: 29739316; PMCID: PMC5941560.*

**Reviewer #3 Comments:**

The authors present a pipeline for identification of protein isoforms using a combination of long-read transcript sequencing with protein mass spectrometry data. Our understanding of protein isoforms is limited, and their identification has been difficult because of the limited coverage protein mass spectrometry. This manuscript provides a much improved method. The manuscript is clearly written and provides a detailed description of the method. I recommend the manuscript for publication as it will likely be widely used to understand the role of isoforms in health and disease.

***Response:*** *We thank the reviewer for the positive comments, and look forward to dissemination of our initial findings for potential expansion of this approach in the genomics and proteomics fields!*

***Additional edits to the manuscript:***

*For Figure 5, which depicts three examples of novel splicing events with peptide support, we determined that additional clarity regarding the source of peptide ESD (Figure 5c) was warranted. We added the following text in the figure legend for Figure 5:*

*“Note that only ESD passed strict manual annotation, but EVR, which passed a 1% FDR in the global MS search, supports the expression of the same terminal exon.”*

*In Figure 6, we noticed a typographical error in the numbering of figure c, d, and e. We have corrected the ordering.*

We have several minor grammatical edits throughout the text, which can be found in the document containing tracked changes.

**Second round of review**

**Reviewer 1**

The authors have addressed all my comments. I have no further questions.

**Reviewer 2**

I thank the authors for their thorough responses to my questions, as well as questions of Reviewer 1. The revised manuscript is much improved and should be a valuable contribution to the field.
